# Double-sided niche regulation in skin stem cell and cancer: mechanisms and clinical applications

**DOI:** 10.1186/s12943-025-02289-8

**Published:** 2025-05-21

**Authors:** Trang Thao Quoc Pham, Yung-Che Kuo, Wei-Ling Chang, Hao-Jui Weng, Yen-Hua Huang

**Affiliations:** 1 International Ph.D. Program in Cell Therapy and Regenerative Medicine, College of Medicine, Taipei Medical University, Taipei, 11031 Taiwan; 2 TMU Research Center for Cell Therapy and Regeneration Medicine, Taipei Medical University, 250 Wuxing Street, Taipei, 11031 Taiwan; 3 Department of Dermatology, Taipei Medical University-Shuang Ho Hospital, New Taipei City, 23561 Taiwan; 4 Department of Dermatology, School of Medicine, College of Medicine, Taipei Medical University, Taipei, 11031 Taiwan; 5 Graduate Institute of Clinical Medicine, College of Medicine, Taipei Medical University, Taipei, 11031 Taiwan; 6 Department of Biochemistry and Molecular Cell Biology, School of Medicine, College of Medicine, Taipei Medical University, Taipei, 11031 Taiwan; 7 Graduate Institute of Medical Sciences, College of Medicine, Taipei Medical University, Taipei, 11031 Taiwan; 8 Center for Reproductive Medicine, Taipei Medical University Hospital, Taipei Medical University, Taipei, 11031 Taiwan

**Keywords:** Niche regulation, Skin stem cell, Cancer stem cell, Drug resistance, Shh/Wnt/YAP/Notch signaling, Therapeutic targeting, Clinical trial

## Abstract

The niche microenvironment plays a crucial role in regulating the fate of normal skin stem cells (SSCs) and cancer stem cells (CSCs). Therapeutically targeting the CSC niche holds promise as an effective strategy; however, the dual effects of shared SSC niche signaling in CSCs have contributed to the aggressive characteristics of tumors and poor survival rates in skin cancer patients. The lack of a clear underlying mechanism has significantly hindered drug development for effective treatment. This article explores recent advances in understanding how niche factors regulate cell fate determination between skin stem cells and skin CSCs, along with their clinical implications. The dual roles of key components of the adhesive niche, including the dermo-epidermal junction and adherens junction, various cell types—especially immune cells and fibroblasts—as well as major signaling pathways such as Sonic hedgehog (Shh), Wingless-related integration site (Wnt)/β-catenin, YAP (Yes-associated protein)/TAZ (transcriptional coactivator with PDZ-binding motif), and Notch, are highlighted. Additionally, recent advances in clinical trials and drug development targeting these pathways are discussed. Overall, this review provides valuable insights into the complex interactions between skin cancer stem cells and their microenvironment, laying the groundwork for future research and clinical strategies.

## Introduction

The epidermis is a mechanically responsive tissue that possesses continuous regenerative potential, serving as a dynamic barrier against environmental challenges [1]. The integrity of the epidermis is sustained by the ongoing self-renewal of long-lived basal-layer skin stem cells (SSCs). Additionally, short-lived transient-amplifying cells (TACs), arising from the mitotic division of stem cells (SCs), detach from the basal layer and undergo terminal differentiation as they move toward the outermost surface. Maintaining a balanced epidermal development is crucial for preserving the protective barrier function and preventing excessive hyperproliferative disorders or skin neoplasms.

The global incidence of skin cancer, including both cutaneous melanoma and nonmelanoma skin cancer (NMSCs), is on the rise [2, 3]. Among these, melanoma is the most lethal, accounting for approximately 75% of skin cancer-related deaths [3] and ranked as the 22nd leading cause of cancer-related deaths worldwide, according to GLOBOCAN 2022 [4]. Melanoma is characterized by a high tendency for metastasis, treatment resistance, and genetic instability. Late-stage melanoma patients exhibit a poor chemotherapy response, with the 5-year survival rate for stage IV melanoma at just 22.5% [5]. In 2022, melanoma ranked as the 17th most common cancer worldwide, with approximately 331,722 new diagnoses and 58,667 fatalities [6]. By 2040, the global incidence of melanoma is projected to increase by 50%, with new cases reaching 510,000 and deaths rising by 68% to 96,000, compared to 2020 [7]. Notably, a significant proportion of melanoma cases (85.6%) and related deaths (67.2%) occurs in countries with a very high human development index [7].

Nonmelanoma skin cancers (NMSCs) primarily consist of two major types: basal cell carcinoma (BCC) and squamous cell carcinoma (SCC). BCC is the most common type, accounting for approximately 70% of cases, while SCC represents about 25% [8]. NMSCs continue to be a significant public health concern, with data indicating that the incidence of BCC has increased by 20% to 80%, and SCC incidence has risen by 3% to 10% annually over the past 30 years [9]. As of 2022, NMSCs accounted for 1,234,533 cancer cases and 69,416 fatalities, according to the GLOBOCAN report [6]. Skin SCC is a highly aggressive form of NMSC that contributed to 56,000 deaths in 2019, according to a global survey [2]. Invasive skin SCC can progress to advanced stages, with a dismal 10-year survival rate of less than 20% [10]. In 2019, there were 2,402,221 global cases of cutaneous SCC, and the death rate increased by 6.1% between 1990 and 2019 [11]. Projections for 2035 estimate an incidence of 3,637,626 cases, reflecting a 51.4% increase from 2019 [11].

The persistent self-renewal and expansion of cancer stem cells (CSCs) in aggressive skin cancer contribute to poor prognosis, significant treatment resistance, and advanced invasion, posing a major challenge in skin cancer treatment [12, 13]. Research has shown that variations in chemoresistance and virulence of melanoma are attributed to the presence of CSCs in tumors, including CD133 (Cluster of differentiation 133) [13], ABCB5 (ATP-binding cassette sub-family B member 5) [14], ALDH (Aldehyde dehydrogenase) [15], CD20 [16], CD271 [17], and SOX10 (SRY-Box transcription factor) [18]. Subpopulations of cells exhibiting CSC properties in SCCs and BCCs are implicated in tumor initiation, progression, and resistance [12, 19, 20].

The fate of both normal skin SSCs and CSCs is predominantly determined by the regulatory influence of the niche microenvironment [21–23]. Notably, certain components of the SSC niche, including core signaling pathways, contribute to the formation and maintenance of the CSC niche. In the normal SSC niche, pathways such as Sonic Hedgehog (Shh), Wingless-related integration site (Wnt)/β-catenin, Notch, and YAP (Yes-associated protein)/TAZ (Transcriptional co-activator with PDZ-binding motif) are vital for maintaining the self-renewal and differentiation of SSCs within tissues [24]. Regulated signaling, in conjunction with other niche components like the adhesion niche, dermal-epidermal interface, and cellular compartments, is essential for preserving SSC homeostasis by preventing uncontrolled proliferation and excessive differentiation [24–26]. In contrast, signaling dysregulation, in collaboration with oncogenic pathways such as phosphoinositide 3-kinase (PI3K)/AKT/mammalian target of rapamycin (mTOR) and Janus kinase (JAK)/ signal transducer and activator of transcription 3 (STAT3), characterizes the CSC niche, particularly in refractory skin cancers [25, 27–29]. The aberrant activation of key signaling pathways or their specific components has been shown to sustain the CSC population and contribute to alternative mechanisms of cancer resistance. In addition to the intrinsic resistance developed by CSCs, these factors complicate the effective elimination of skin cancers.

**Fig. 1:**
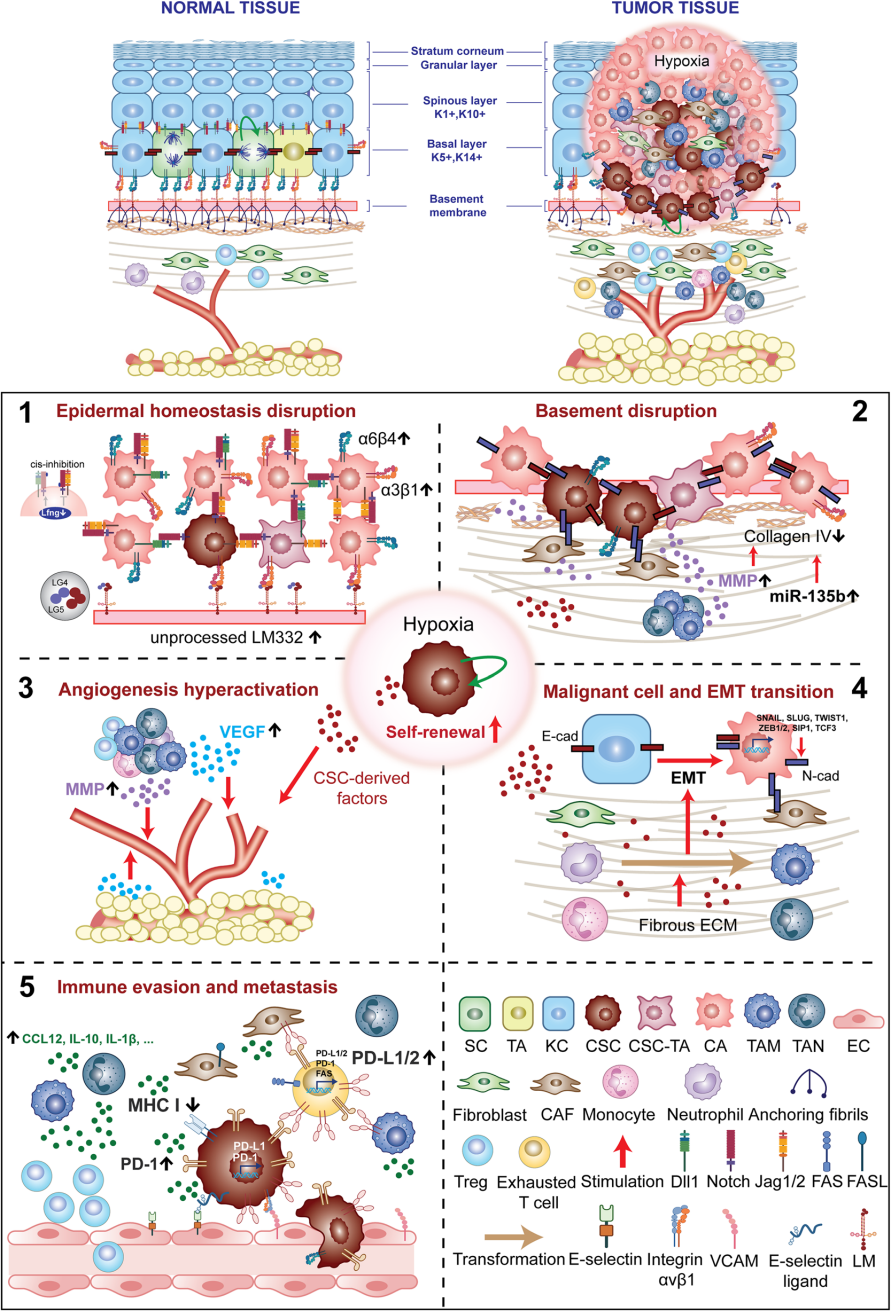
The niche for normal and cancer skin stem cells. The illustration delineates the distinguishing characteristics between niche factors supporting normal SSC and skin CSC. The intricate and heterogeneous nature of the skin CSC niche is depicted through five primary features. **1** Epidermal homeostasis is disrupted by the redistribution of integrins (α6β4 and α3β1), resulting in elevated suprabasal integrin levels, and dysregulated maturation of LM332, which presents as unprocessed LM332, remaining the LG4–5 domain and full length of the β3 chain, or its subunit γ2 fragments. The Notch signaling pathway can become dysfunctional, in which Notch is downregulated, leading to the release of its inhibitory effects on stem cell proliferation, thereby inducing skin tumorigenesis. However, Notch1 and its ligands, DLL1 and JAG1/2, are highly expressed in tumor cells during skin cancer metastasis. These opposing functions of Notch signaling may be associated with the ectopic presence of DLL1 in tumor cells, which are typically confined to the SSCs of the epidermal basal layer, and increased expression of JAG1/2. Additionally, alterations in the affinity of Notch1 receptor for its ligands could further contribute to this complexity. This may result from reduced LFNG expression, which reinforces DLL1-Notch cis-inhibition (*indicated by the light grey arrow*) and inhibits JAG-Notch cis-inhibition in the physiological SSC niche (*indicated by the light grey line*), in progressive tumors. **2** Disruption of basement membrane integrity occurs through uncoupling of integrin from unprocessed LM332, breakdown of cadherin-mediated adhesion during EMT, and impairment of collagen IV, possibly induced by MMPs or miRNA-135b. Elevated MMP activity is sustained by CSCs, dysfunctional ECM proteins, and malignant cellular compartments, including CAFs, TAMs and TANs. The enhanced stiffness of the dermal matrix prompts the aberrant expression of integrins, which contribute to sustaining the stemness of CSCs. **3** Factors secreted by CSCs and tumor-associated cells, particularly CAFs, TANs, and TAMs, include molecules like VEGF and MMPs, which promote angiogenesis. **4** The continuous conversion of fibroblasts, monocytes, and neutrophils into CAFs, TAMs, and TANs, respectively, is imperative for maintaining the CSC niche. Concurrently, factors secreted by CSCs, fibrous ECM, and hypoxic conditions perpetuate the malignant traits of fibroblasts, macrophages, and neutrophils. Additionally, the transition from E-cadherin to N-cadherin in adherens junctions, driven by the increased expression of *SNAIL*, *SLUG*, *TWIST1*, *ZEB1/2*, *SIP1*, and *TCF3*, as well as secreted factors from CSCs or tumor-associated cells like TGF-β, promotes metastasis and facilitates the recruitment of CAFs into the CSC niche. **5** The CSC niche sustains the survival of CSCs and tumor cells through multiple mechanisms. Cellular constituents within the CSC niche, including CAFs, TAMs, TANs, and Tregs, orchestrate an immunosuppressive TME, thereby inhibiting cytotoxic T cell function. Notably, soluble factors secreted by these immunosuppressive cells and CSCs, such as IL-10, CCL12, CXCR2 and IL-1β, promote Treg accumulation within the CSC niche and upregulate the expression of PD-1 and PD-L1 in both CSCs and tumor cells. CAFs can impair T cell function and increase T cell apoptosis by binding their FASL and PD-L1/2 to FAS and PD-1 receptors on T cells. CSCs further contribute to immune evasion by elevating PD-1 expression and downregulating MHC I/II expression on their surfaces. Endothelial cells (ECs) not only facilitate angiogenesis to nourish the CSC niche but also promote skin cancer metastasis through interactions with adhesive proteins on CSCs. *SC, Stem cell; TA, Transamplifying cell; KC, Keratinocytes; CSC-TA, Cancer stem cell-transamplifying cell; CA; Cancer cell;* *SSC, skin stem cell; CSC, cancer stem cell; LM332, Laminin 332; LG4-5, Laminin globular 4-5; DLL1, Delta-like ligand 1; JAG1/2, Jagged1/2; LFNG, Lunatic Fringe; EMT, Epithelial-mesenchymal transition; MMP, Matrix metalloproteinase, ECM, Extracellular matrix; CAF, Cancer-associated Fibroblast; TAMs, Tumor-associated Macrophage; TANs, Tumor-associated Neutrophil; VEGF, Vascular Endothelial Growth Factor; SNAIL, Snail Family Transcriptional Repressor 1; SLUG, Snail Family Transcriptional Repressor 2; TWIST1, Twist family bHLH transcription factor 1; ZEB1/2, Zinc finger E-box binding homeobox 1/2; SIP1, Smad interacting protein 1; TCF3, Transcription factor 3, EC, Endothelial cell; Treg, Regulatory T cells; FAS, Fas cell surface death receptor,; FASL, FAS ligand; MHC I, Major Histocompatibility Complex 1; PD-1, Programmed Cell Death Protein 1; PD-L1, Programmed cell death ligand 1; IL-10, Interleukin 10; CCL12, C–C Motif Chemokine Ligand 12; CXCR2, C-X-C Motif Chemokine Receptor 2; VCAM, Vascular Cell Adhesion Molecule; E-cad, E-cadherin; N-cad, N-cadherin.* Some figure components were created in BioRender. Pham, Q. (2025) https://BioRender.com/z83f047

**Fig. 2:**
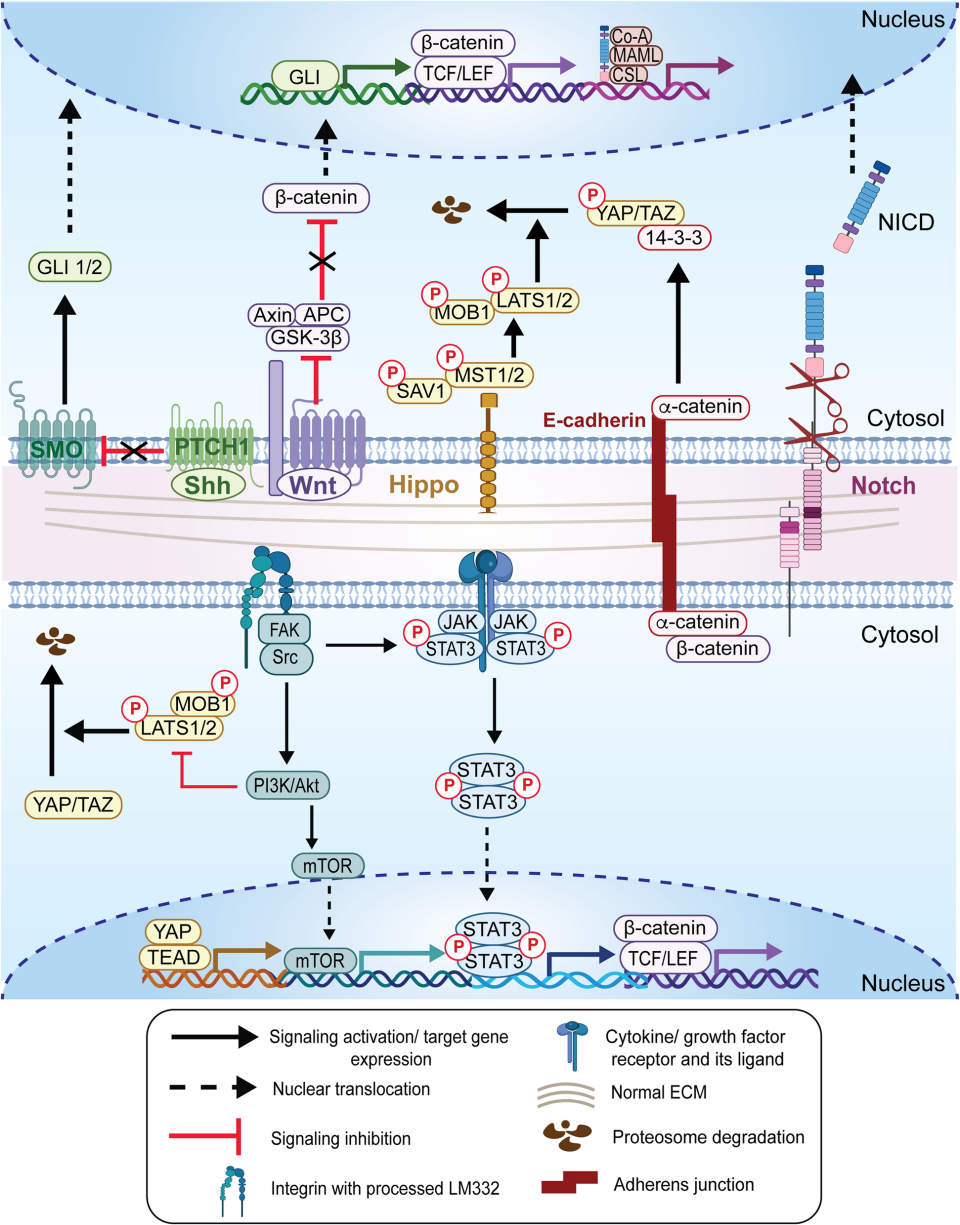
Core signaling pathways in skin stem cell niche. The schematic diagram emphasizes the core signaling pathways, demonstrating their role in orchestrating the physiological development of skin stem cells. Notably, Notch signaling promotes epidermal differentiation while also exerting negative control on Wnt and Shh signaling pathways to prevent unregulated epidermal proliferation. Under normal circumstances, the activity of YAP/TAZ is constrained by the Hippo pathway, accompanied by adherens junctions, which enhance the formation of the 14–3-3-YAP complex, leading to proteasomal degradation of YAP. Furthermore, α-catenin in adherens junctions binds to β-catenin, impeding its nuclear translocation of β-catenin, consequently inhibiting skin stem cell differentiation and maintaining the skin stem cell compartment. *SMO, smoothened; PTCH, Patched; Gli, Glioma-associated oncogene homolog; NICD, Notch intracellular domain; TCF/LEF, T-cell factor/lymphoid enhancer-binding factor; LATS1/2, Large tumor suppressor 1 and 2; MST1/2, Mammalian sterile20-like 1 and 2; YAP/TAZ, Yes-associated protein/Transcriptional coactivator with PDZ-binding motif; TEAD, TEA domain transcription factor; FAK, Focal adhesion kinase; ECM, Extracellular matrix; PI3K/AKT/mTOR, Phosphoinositide 3-kinase/AKT/Mammalian target of rapamycin; JAK/STAT3, Signal transducer and activator of transcription 3*. Some figure components were created in BioRender. Pham, Q. (2025) https://BioRender.com/z83f047

**Fig. 3:**
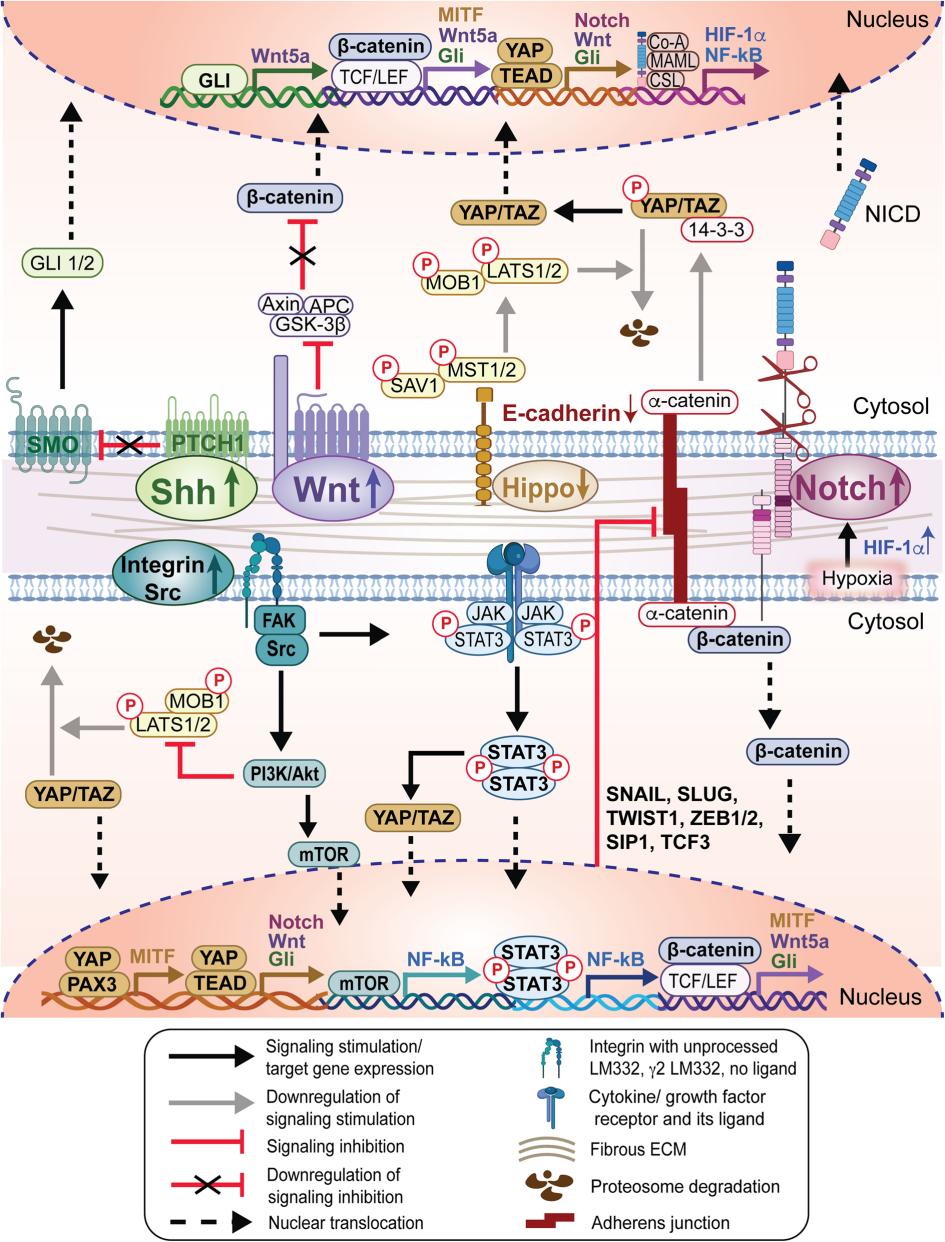
Signaling network in skin cancer stem cell niche. Overactivation of core signaling pathways, encompassing Shh, Wnt, YAP/ TAZ, and Notch, augments the expression of molecules associated with proliferation, thereby fostering the expansion and persistence of CSCs. Dysregulated expression of E-cadherin and α-catenin disrupts the cytoplasmic sequestration of β-catenin, potentiating Wnt signaling, while concurrently diminishing the association between 14–3-3 and YAP, thereby attenuating YAP proteasomal degradation. Stimulation of integrin-SRC signaling by stiff ECM promotes CSC proliferation by upregulating the PI3K/AKT/mTOR and JAK/STAT3 signaling pathways. Subsequently, these pathways facilitate the upregulation of YAP/TAZ and cell-cycle gene expression, disrupt adherens junctions, and prevent apoptosis in tumor cells. Within the CSC niche, modulation of inflammation, attenuation of Hippo signaling, and stimulation of Notch and JAK/STAT3 pathways potentiate the activity of YAP/TAZ. Hyperactivation of YAP/TAZ signaling induces cis-inhibition of Notch signaling, thereby impeding CSC differentiation. The maintenance of CSC quiescence by Notch signaling is reinforced by HIF-1α, which is stabilized within the hypoxic tumor microenvironment, along with regulatory crosstalk involving the Wnt/β-catenin, Shh, and YAP/TAZ signaling pathways. The migration and metastasis of skin cancers are facilitated by EMT, which is initiated by the switch from E-cadherin to N-cadherin. This shift is enhanced by factors secreted from CSCs and cellular compartments within the ECM, alongside the hyperactivation of key signaling pathways in the CSC niche. Notable pathways involved include Wnt/β-catenin, Notch, Shh/GLI, PTEN/PI3K, and JAK/STAT3 pathways. These pathways contribute to the upregulation of mesenchymal phenotype markers, such as *SNAIL*, *SLUG*, *TWIST1*, *ZEB1/2*, *SIP1*, and *TCF3*, facilitating the transition to a more invasive phenotype of skin tumors. *CSC, cancer stem cell; SMO, smoothened; PTCH, Patched; Gli, Glioma-associated oncogene homolog; NICD, Notch intracellular domain; TCF/LEF, T-cell factor/lymphoid enhancer-binding factor; LATS1/2, Large tumor suppressor 1 and 2; MST1/2, Mammalian sterile20-like 1 and 2; YAP/TAZ, Yes-associated protein/Transcriptional coactivator with PDZ-binding motif; TEAD, TEA domain transcription factor; FAK, Focal adhesion kinase; ECM, Extracellular matrix; PI3K/AKT/mTOR, Phosphoinositide 3-kinase/AKT/Mammalian target of rapamycin; JAK/STAT3, Signal transducer and activator of transcription 3; HIF, hypoxia-inducible factor; EMT, Epithelial-mesenchymal transition; SNAIL, Snail Family Transcriptional Repressor 1; SLUG, Snail Family Transcriptional Repressor 2; TWIST1, Twist family bHLH transcription factor 1; ZEB1/2, Zinc finger E-box binding homeobox 1/2; SIP1, Smad interacting protein 1; TCF3, Transcription factor 3; NF-κB, Nuclear factor-kappa B; MITF, Microphthalmia-associated transcription factor; PAX3, Paired box gene 3.* Some figure components were created in BioRender. Pham, Q. (2025) https://BioRender.com/z83f047

**Fig. 4:**
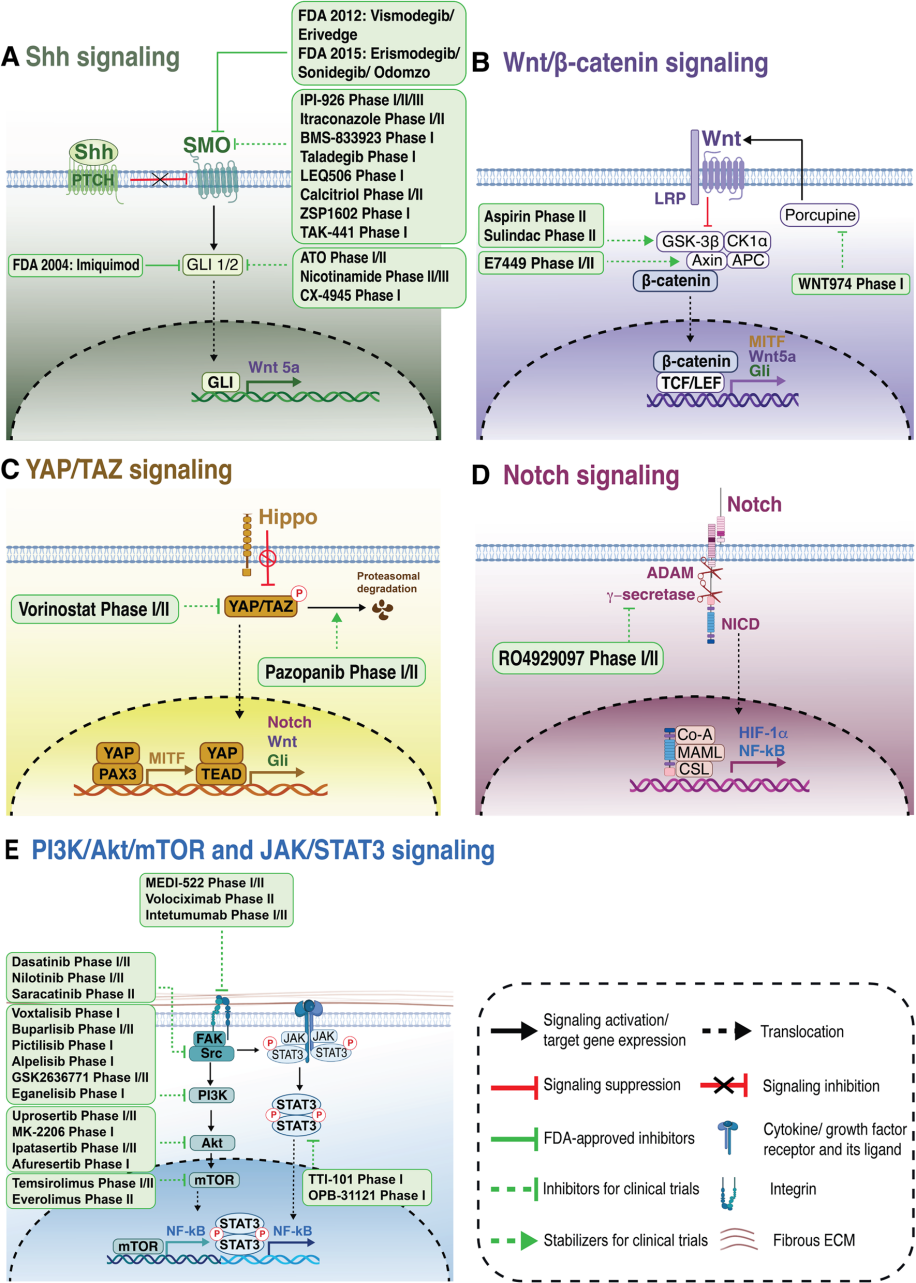
Drug targeting on skin cancer stem cell niche. The figure depicts the principal signaling pathways involved in the regulation of the skin cancer stem cell niche, alongside an overview of FDA-approved drugs and those currently undergoing clinical trials for the treatment of skin cancer. These therapeutic agents encompass: **A** inhibitors targeting SMO and GLI expression within the Shh signaling pathway; **B** suppressors of porcupine and promoters of β-catenin degradation complex activity within the Wnt/β-catenin signaling cascade; **C** inhibitors targeting YAP/TAZ signaling activity; **D** γ-secretase inhibitors targeting Notch signaling; and **E** inhibitors blocking integrin-related signaling pathways, such as FAK/Src, PI3K/AKT/mTOR and JAK/STAT3 signaling pathways, and those targeting integrins, which are typically absent in the skin stem cell niche. *FDA, Food and Drug Administration; SMO, Smoothened; PTCH, Patched; Gli, Glioma-associated oncogene homolog; NICD, Notch intracellular domain; TCF/LEF, T-cell factor/lymphoid enhancer-binding factor; HIF, hypoxia-inducible factor; YAP/TAZ, Yes-associated protein/Transcriptional coactivator with PDZ-binding motif; TEAD, TEA domain transcription factor; ECM, Extracellular matrix; FAK, Focal adhesion kinase; PI3K/AKT/mTOR, Phosphoinositide 3-kinase/AKT/Mammalian target of rapamycin; JAK/STAT3, Signal transducer and activator of transcription 3; NF-κB, Nuclear factor-kappa B; PAX3, Paired Box 3; MITF, Microphthalmia-Associated Transcription Factor*. Some figure components were created in BioRender. Pham, Q. (2025) https://BioRender.com/z83f047

Patients with aggressive skin tumors often face a poor prognosis, marked by significant treatment resistance and advanced invasion. However, the development of effective therapies for these tumors remains challenging. Targeting the CSC niche offers a promising strategy, particularly for refractory skin cancers. This review provides an overview of the latest updates on the niche regulation of skin SSCs (Figs. 1 and 2) and skin CSCs (Figs. 1 and 3), focusing on the underlying mechanisms and current clinical trials that target CSC niche signaling for the treatment of skin cancers, including SCCs, BCCs, and melanoma (Fig. 4 and Tables 1 and 2). The insights presented in this review will facilitate future research and advance clinical strategies, carrying significant implications for the treatment of skin cancer.

**Table 1:** Targeting signaling pathways associated with skin cancer stem cells

Pathway	Drug	Mechanism of action	Combined treatment	Conditions	Clinical trial phase	NCT Number
**Sonic Hedgehog pathway**	GDC-0449 (Vismodegib/Erivedge) Approved by FDA 2012	SMO inhibitor	-	Resectable, or treated (recurrent, or failed other chemotherapy) BCC	Phase I	NCT01631331
			Photodynamic therapy	Multiple BCCs		NCT02639117
			-	Metastatic or locally advanced BCC		NCT00607724
			-	Resectable BCC	Phase II	NCT01543581
			-	Metastatic or locally advanced BCC		NCT00833417
			-	New (not recurrent or previously treated) nodular BCC		NCT01201915
			-	Large and/or recurrent resectable BCC		NCT03035188
			-	BCC with surgery stage A, B or C		NCT02667574
			-	Multiple BCCs		NCT01815840
			-	Metastatic or locally advanced BCC		NCT01367665
			-	Clinically suspicious or locally advanced BCC		NCT01700049
			-	Locally advanced BCC		NCT01835626
			Topical aminolevulinic acid 20% (photosensitizing agents)	Multiple BCCs		NCT01556009
			FOLFOX (folinic acid [leucovorin], fluorouracil [anti-metabolite], and oxaliplatin [OX]), FOLFIRI (Folinic acid [leucovorin], fluorouracil [anti-metabolite], and irinotecan [TOP1 inhibitor]) and Bevacizumab (antiangiogenic agent)	Metastatic or locally advanced BCC		NCT00959647
			Pembrolizumab (PD-1 inhibitor)	Metastatic or unresectable BCC	Phase I/II	NCT02690948
			-	Locally advanced or recurrent BCC	Phase IV	NCT02436408
			-	Metastatic or locally advanced BCC	-	NCT01160250
			-	Advanced BCC		NCT02371967
			-	Locally advanced BCC		NCT02781389
			-	Locally advanced BCC		NCT02674009
			-	Metastatic or locally advanced BCC		NCT02438644
	LDE225 (Erismodegib/ Sonidegib/ Odomzo) Approved by FDA 2015	SMO inhibitor	-	Advanced BCC	Phase I	NCT01208831
			-	Advanced BCC		NCT00880308
			-	Locally advanced and metastatic BCC	Phase II	NCT01327053
			Imiquimod	Resectable and invasive BCC		NCT03534947
			-	Multiple BCCs		NCT00961896
			-	Multiple BCCs		NCT01350115
			-	Locally advanced or metastatic BCC (previously received a non-LDE225 Smo inhibitor)	-	NCT01529450
			Pembrolizumab (PD-1 inhibitor)	Unresectable or metastatic melanoma and stage IV SCC	Phase I	NCT04007744
	IPI-926 (Saridegib/ Patidegib)	SMO inhibitor	-	BCC	-	NCT01609179
			-	Advanced and/or metastatic BCC	Phase I	NCT00761696
			-	Sporadic nodular BCC	Phase II	NCT02828111
			-	Surgically eligible BCCs		NCT02762084
			-	Basal cell nevus syndrome	Phase III	NCT03703310
	Itraconazole	SMO inhibitor	-	BCC	Early phase I	NCT02120677
			-		Phase II	NCT01108094
	BMS-833923 (XL139)	SMO inhibitor	-	Advanced or metastatic BCC	Phase I	NCT00670189
	Taladegib (LY2940680)	SMO inhibitor	-	Advanced or metastatic BCC	Phase I	NCT01226485
	LEQ506	SMO inhibitor	-	Metastatic or locally advanced BCC	Phase I	NCT01106508
	Vitamin D3 (calcitriol)	SMO inhibitor	Temozolomide (alkylating agent)	Malignant melanoma	Phase I/II	NCT00301067
			Diclofenac (NSAID)	Nodular or superficial BCC	Phase II	NCT01358045
	ZSP1602	SMO inhibitor	-	Advanced BCC	Phase I	NCT03734913
	TAK-441	SMO inhibitor	-	Advanced BCC	Phase I	NCT01204073
	Arsenic trioxide	Gli inhibitor	-	BCC	Phase I/II	NCT01791894
	Nicotinamide		-	SCC, BCC	Phase II	NCT03769285
			-		Phase III	ACTRN12612000625875
	CX-4945 (CK2 inhibitor; silmitasertib)		-	Metastatic BCC or locally advanced BCC	Phase I	NCT03897036
	Imiquimod Approved by FDA 2004		Cryosurgery	Superficial and nodular BCC	-	NCT01212562
			-		Phase III	NCT00066872
			-			NCT00129519
			-			NCT00189241
			-			NCT00189306
			Cryosurgery			NCT01212549
			-		Phase IV	NCT00204555
			-			NCT00314756
			Interferon alpha-2β (Intron-A)			NCT00581425
**Wnt pathway**	LGK974 (WNT974)	Porcupine inhibitor	± PDR001 (PD-1 inhibitor)	Locally advanced or metastatic melanoma	Phase I	NCT01351103
	E7449	Axin stabilization	± Temozolomide (alkylating agent) or ± Carboplatin (alkylating agent) and Paclitaxel (mitotic inhibitor)	Advanced or metastatic melanoma	Phase I/II	NCT01618136
	Aspirin	β-catenin degradation by increasing phosphorylation of GSK3β	Pembrolizumab (PD-1 inhibitor) and Ipilimumab (CTLA-4 inhibitor)	Metastatic or unresectable melanoma	Phase II	NCT03396952
	Sulindac		Epirubicin (cytotoxic antibiotic)	Metastatic malignant melanoma	Phase II	NCT00755976
**Notch pathway**	RO4929097	γ-secretase inhibitor	Cisplatin (alkylating agent), Vinblastine (alkaloid agent), and Temozolomide (alkylating agent)	Recurrent or metastatic melanoma	Phase I/II	NCT01196416
**YAP/TAZ pathway**	Vorinostat (HDAC inhibitor)	*YAP* transcription suppressor	Doxorubicin (cytotoxic antibiotic)	Melanoma	Phase I	NCT00331955
			-	Advanced melanoma with BRAF V600 mutation and progression of disease, while on treatment with BRAFi or a combination of BRAF and MEK inhibitors	Phase I/II	NCT02836548
			-	Metastatic or unresectable melanoma	Phase II	NCT00121225
	Dasatinib	Src inhibitor	Dacarbazine (alkylating agent)	Stage IV or unresectable stage III melanoma	Phase I/II	NCT00597038
			-	Unresectable locally advanced or metastatic melanoma	Phase II	NCT00700882
			-	Unresectable locally advanced or metastatic melanoma	Phase II	NCT00436605
			Dendritic cell vaccines	Stage IV or unresectable stage IIIB/C melanoma		NCT01876212
			-	Unresectable or metastatic SCC	Phase II	NCT00563290
	Nilotinib		Dabrafenib (BRAF inhibitor) and Trametinib (MEK inhibitor)	Unresectable melanoma with BRAF V600 mutation and failed or have stable disease on any BRAFi/MEKi regimen	Phase I	NCT04903119
			-	Metastatic melanoma	Phase II	NCT00788775
			-	Unresectable locally advanced or metastatic melanoma with c-KIT mutation		NCT01395121
			Dacarbazine (alkylating agent)	Unresectable or metastatic melanoma with c-KIT mutation		NCT01028222
	Saracatinib		-	Metastatic melanoma	Phase II	NCT00669019
	Pazopanib	Inhibition of nuclear YAP translocation by enhancing proteasomal degradation	Alisertib (AURKA inhibitor)	Advanced melanoma	Phase I	NCT01639911
			-	Unresectable malignant melanoma	Phase II	NCT00861913
			Paclitaxel (mitotic inhibitor)	Stage IV or unresectable stage III melanoma		NCT01107665
**PI3K/AKT/ mTOR pathway**	Voxtalisib (SAR245409)	pan-PI3K mTOR inhibitor	Pimasertib (MEK inhibitor)	Locally advanced or metastatic melanoma	Phase I	NCT01390818
	Buparlisib (BKM120)	pan-PI3K inhibitor	Vemurafenib (BRAF inhibitor)	Unresectable stage III and stage IV melanoma with BRAFV600E/K mutation	Phase I/II	NCT01512251
			MEK162 (MEK inhibitor)	Advanced unresectable melanoma	Phase I	NCT01363232
			LGX818 (BRAF inhibitor) and MEK162 (MEK inhibitor)	Unresectable stage III or metastatic melanoma with BRAF V600 mutation	Phase II	NCT02159066
	Pictilisib (GDC-0941)	PI3K inhibitor	-	Locally advanced or metastatic melanoma	Phase I	NCT00876122
	Alpelisib (BYL719)	PI3Kα inhibitor	MEK162 (MEK inhibitor)	Advanced melanoma	Phase I	NCT01449058
	GSK2636771	PI3Kβ inhibitor	Pembrolizumab (PD-1 inhibitor)	Refractory, unresectable stage III or stage IV metastatic melanoma with PTEN loss	Phase I/II	NCT03131908
	Eganelisib (IPI-549)	PI3Kγ inhibitor	Nivolumab (PD-1 inhibitor)	Advanced and/or metastatic melanoma	Phase I	NCT02637531
	Temsirolimus (CCI-779)	mTOR inhibitor	HCQ (autophagy inhibitor)	Refractory, metastatic melanoma	Phase I	NCT00909831
			-	Metastatic melanoma	Phase II	NCT00022464
	Everolimus (RAD001)		Bevacizumab (antiangiogenic agent)	Unresectable stage IV melanoma, or recurrent melanoma with metastases	Phase II	NCT00591734
			Paclitaxel (mitotic inhibitor) and Carboplatin (alkylating agent)	Unresectable, stage IV melanoma		NCT01014351
			± Carboplatin (alkylating agent), Paclitaxel (mitotic inhibitor), and Bevacizumab (antiangiogenic agent)	Unresectable, stage IV melanoma		NCT00976573
			-	Stage IV melanoma	Phase II	NCT00098553
			Temozolomide (alkylating agent)	Stage IV melanoma and unresectable metastatic melanoma		NCT00521001
	Uprosertib (GSK 2141795)	Akt inhibitor	GSK1120212 (MEK inhibitor)	BRAF wild-type melanoma	Phase I	NCT01138085
			Trametinib (MEK inhibitor)	Unresectable stage III or stage IV melanoma	Phase II	NCT01941927
			Trametinib (MEK inhibitor) Dabrafenib (BRAF inhibitor)	Stage IIIC-IV melanoma	Phase I/II	NCT01902173
	MK-2206		Carboplatin (alkylating agent) and paclitaxel (mitotic inhibitor) or Docetaxel (mitotic inhibitor) or Erlotinib (EGFR inhibitor)	Locally advanced or metastatic melanoma	Phase I	NCT00848718
			HCQ (autophagy inhibitor)	Advanced melanoma		NCT01480154
	Ipatasertib (GDC-0068)		Atezolizumab (PD-L1 inhibitor)	Melanoma post progression on immune-checkpoint inhibitors	Phase I/II	NCT03673787
	Afuresertib (GSK2110183)		Trametinib (MEK inhibitor)	Melanoma	Phase I	NCT01476137
**JAK/STAT3 pathway**	TTI-101	STAT3 inhibitor	-	Locally advanced, inoperable, metastatic and/or treatment refractory melanoma	Phase I	NCT03195699
	OPB-31121		-	Advanced melanoma		NCT00657176
**Integrin**	MEDI-522 (Etaracizumab, Abegrin, or Vitaxin)	αvβ3 integrin inhibitor	-	Unresectable, stage IV or recurrent malignant melanoma	Phase I	NCT00111696
			± Dacarbazine (alkylating agent)	Unresectable, stage IV metastatic melanoma	Phase II	NCT00066196
	Volociximab	α5β1 inhibitor	Dacarbazine (alkylating agent)	Stage IV or unresectable stage III melanoma	Phase II	NCT00099970
	Intetumumab	αv integrin inhibitor	± Dacarbazine (alkylating agent)	Stage IV or unresectable stage III melanoma	Phase I/II	NCT00246012
The clinical trial information was accessed at US National Library of Medicine, *ClinicalTrials.gov*, https://clinicaltrials.gov with the National clinical trial number (NCT number) and https://www.anzctr.org.au with ONTRAC Australian New Zealand clinical trials registry number (ACTRN)						

*CK2 casein kinase, HCQ Hydroxychloroquine, EGFR Epidermal Growth Factor Receptor, PD-1 Programmed Cell Death Protein 1, CTLA-4 Cytotoxic T-Lymphocyte Antigen 4, MEK Mitogen-Activated Protein Kinase Kinase, AURKA Aurora kinase A, NSAID Non-Steroidal Anti-Inflammatory Drug, TOP1 Topoisomerase 1*

## The dermal–epidermal niche in SSC and CSC maintenance

SSCs, which primarily include interfollicular epidermal stem cells (IFESCs), hair follicle stem cells (HFSCs), and melanocyte stem cells (MSCs), are widely recognized for their roles in maintaining the skin epidermis through self-regeneration and terminal differentiation [1]. In the epidermal basal layer, IFESCs are located and function in regulating the regeneration of the interfollicular epidermis (IFE) [30]. HFSCs are crucial for driving HF growth and are predominantly found in the hair follicle (HF) bulge region. During the physiological anagen phase, the outer root sheath and sebaceous ducts are formed by differentiated keratinocytes originating from HFSCs. In wound healing, however, HFSCs contribute to maintaining the IFESC population [31–33]. Skin pigmentation is regulated by melanin, synthesized by melanocytes originating from MSCs in the IFE. MSCs in the lower bulge of the HF generate melanocytes, which are essential for hair shaft pigmentation [34]. Hair pigmentation is continuously maintained through the cyclical activation of HFSCs, which is regulated by paracrine signals from the bulge and dermal papilla (DP). This process is closely synchronized with the activation of MSCs [35].

**Table 2:**
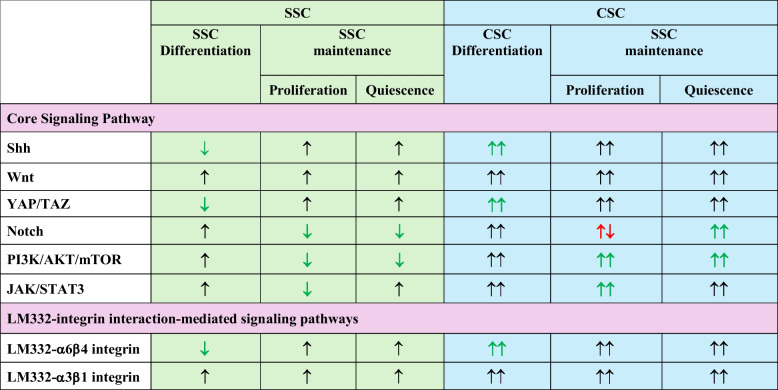
The dual roles of core signaling pathway in SSC and CSC niche

Key signaling pathways that regulate SSC differentiation, proliferation and stemness are essential for the maintenance and expansion of CSCs. Dysregulation and imbalance of these pathways are observed in the CSC niche, promoting cancer metastasis while sustaining the CSC population. Positive regulation of signaling pathways is represented by an upward-pointing arrow, while inhibition of these pathways, affecting SSC and CSC differentiation, proliferation or quiescence, is indicated by a downward-pointing arrow. A green arrow indicates the altered function of a signaling pathway in the SSC and CSC niche. A red arrow denotes the dual function of the signaling pathway, which can either activate or inhibit cellular processes

It has been demonstrated that the survival of SSCs relies heavily on the niche environment, which comprises the dermal-epidermal basement membrane (dermal-epidermal BM), cell–cell adhesive contacts, cellular compartments, and signaling pathways. Considering the critical roles of IFESCs and HFSCs in maintaining epidermal homeostasis and their involvement as key sources of common nonmelanoma skin cancers (SCC and BCC) [36, 37], as well as their regulation of melanoma cell origins in the normal SSC niche [35], this review will primarily focus on the IFESC and HFSC populations of SSCs. The term “SSCs” will be discussed in relation to these specific stem cell populations throughout the review.

### Dermal–epidermal basement membrane (Dermal-epidermal BM)

To maintain a balance in epidermal development during regeneration, the connection between the dermis and epidermis, known as the dermal-epidermal BM, provides an adhesive scaffold and mechanical forces to stem cells or facilitates important signaling processes involving growth factors and extracellular matrix (ECM) proteins [38]. In the skin’s structure, the dermal-epidermal BM contains a wealth of ECM and various molecules that promote communication and separation between the epidermis and dermis [38]. Key ECM proteins in the BM, such as laminin (LM) and collagen IV, bind to integrin receptors on basal SSCs, forming anchoring complexes that orchestrate the coordination of actin and microtubule networks within these cells [38]. This interaction influences the behavior of both normal SSCs and CSCs.

#### Laminin-integrin interaction in SSC and CSC regulation

- Laminin-integrin interaction in SSC niche

##### Laminin 332 (LM332) and laminin 511 (LM511), and their integrin receptors

Laminins (LMs) are multifunctional non-collagenous components of BM which mainly contribute to the skin anchorage structure and maintain SSC homeostasis. LM332 and LM511 are the most abundant laminins in the BM. LM332 is primarily located beneath IFESCs and less abundant in the upper HF [39], playing a key role in regulating epidermal differentiation [40]. In contrast, LM511 is predominantly found in deeper regions of the HF, with lower presence around basal IFESCs. It is integral to sustaining HFSCs and facilitating the progression of HF development [39].

LM332 and LM511 differ in their structure, distribution, and interaction with integrin receptors, resulting in their distinct roles in maintaining skin homeostasis. LM332 and LM511 share similar primary integrin receptors (α6β4 and α3β1) [40]. The skin integrins α6β4 and α3β1 serve as the main cell surface receptors on SSCs, interacting with LMs in the BM to enhance proliferation and maintain the quiescence of SSCs [41]. These transmembrane receptors are dual-direction signal transmitters, conveying information between the external environment and cell interior [42]. To ensure stable connections between SSCs and the ECM, they aggregate with intracellular adaptor proteins that bind to the cytoskeleton or actin filaments in SSCs [42]. In intact skin, the interaction of LM332 with the α6β4 integrin is more prominent than its binding to α3β1 integrin [43]. Conversely, the interaction between LM511 and α3β1 is regarded as dominant [44].

Integrin α6β4 and α3β1 receptors have distinct distribution patterns on the SSC membrane, facilitating their interaction with LMs to maintain SSC quiescence. α3β1 integrin, present on both the basolateral and apical surfaces of basal SSCs [43], interacts with LM332 and LM511 to constitute focal adhesions (FAs) [44]. In contrast, α6β4 is concentrated on the membrane surface of SSCs adjacent to the BM [43]. This arrangement enables α6β4 integrin to effectively bind to processed LM332, stabilizing basal SSCs in the BM through hemidesmosome formation [43]. Moreover, the expression of α6β4 and α3β1 is confined to the basal epidermal layer, where SSCs show high levels of these integrins, whereas TACs exhibit considerably lower levels [45, 46].

##### LM332 and α6β4 integrin: SSC population maintenance

LM332 and its primary receptor, α6β4 integrin, help maintain the SSC pool by preserving the integrity of the epidermal-dermal junction [40]. To perform its functions, LM332, unlike LM511, is secreted by keratinocytes as a precursor containing full-length α3, β3, and γ2 chains. It undergoes extensive proteolytic processing after secretion and deposition, which is required for its interactions with surface receptors and other ECM components [47]. The matured or processed LM332, resulting from these maturation events, is the primary form found in the BM of normal skin [47]. The laminin globular (LG)4–5 fragment is released when the 190–200 kDa α3 subunit is cleaved within the C-terminal region between the LG3 and LG4 subdomains. Meanwhile, a 105 kDa subunit is generated following cleavage of the 160 kDa γ2 subunit at its N-terminus [48, 49]. Following the initial partial proteolytic cleavage, LM332 self-organizes into polymer networks in the BM by indirectly connecting to the keratin intermediate filaments of basal SSCs through its α6β4 integrin receptors and to anchoring fibrils in the dermis via collagen VII, thereby forming stable hemidesmosomes [50, 51]. Therefore, hemidesmosomes act as a bridge to stabilize SSC-matrix adhesion and maintain BM assembly [49, 52]. The interaction between α6β4 integrin and processed LM332, embedded in stable hemidesmosome complexes, primarily restricts migration by ensuring basal SSC adhesion in the BM niche [52]. During epidermal regeneration, the dissociation of hemidesmosomes facilitates the detachment of basal keratinocytes from the BM, thereby promoting their differentiation. The γ2 subunit of LM332 may undergo further N-terminal cleavage by MMPs (Matrix metalloproteinases) during epidermal differentiation, resulting in a smaller protein EGF-like segment [53, 54]. EGFR (Epidermal growth factor receptor)-induced serine phosphorylation of the intracellular β4 tail is initiated by the EGF-like fragment [55, 56]. As a result, the interaction between α6β4 integrin and its adaptor protein plectin is disrupted, potentially destabilizing hemidesmosomes and enhancing cell migration [55, 57].

LM332 not only serves a structural role in stable hemidesmosomes but also modulates SSC behavior by participating in signaling through α6β4 integrins in hemidesmosomes and α3β1 integrins in FAs [52]. Tyrosine phosphorylation of β4 integrin in its intracellular tail occurs when α6β4 binds to the mature extracellular LM332 ligand [58]. This phosphorylated form of β4 can sequentially recruit and attach to the Src homology 2 domain containing (SHC) adaptor protein, and growth factor receptor-bound protein 2 (GRB2) [58–60]. The association of α6β4 with these adaptor proteins potentially links α6β4 to RAS (Rat sarcoma virus) and activates two distinct MAPK (Mitogen-activated protein kinase) signaling pathways, both RAS/ERK (Extracellular signal-regulated kinase) and RAC (Ras-related C3 botulinum toxin substrate)/JNK (c-Jun NH2-terminal kinase) signaling pathways, which control cell division in response to mitogens [59, 60]. Concurrently, α6β4 integrin-mediated quiescent SSCs may be reinforced by RAC1 (Rac family small GTPase 1), which exerts a negative regulatory effect on c-MYC stability [61]. Furthermore, c-MYC activation in keratinocytes causes a marked reduction in α6β4 integrin localization at the cell surface and hemidesmosomal junctions, impairing hemidesmosome assembly [62]. Overexpression of c-MYC transcriptionally results in the downregulation of integrin genes α6 (*ITGA6*), β1 (*ITGB1*), and β4 (*ITGB4*), causing stem cells to exit their BM niche and undergo premature terminal differentiation [63]. These findings point to the critical involvement of LM332 and integrin α6β4 in sustaining SSC populations.

##### LM332 and α3β1 integrin: SSC quiescence and migration

The interaction between LM332 and integrin α3β1 contributes to the dynamic dimensions of FAs, which are crucial for preserving quiescent SSCs. FAs in the basal epidermal layer not only transmit cytoskeletal forces but also act as key hubs for converting external signals into intracellular pathways [64]. FAs are initiated through the linkage of α3β1 integrins to LM332, with adaptor proteins such as talin, vinculin, and integrin-linked kinase (ILK) providing structural connections between the cytoplasmic β1 domain and the actin cytoskeleton [65]. The α3β1-LM332 interaction activates the MAPK pathway, essential for SSC proliferation, while also triggering focal adhesion kinase (FAK)/SRC/RAC1 signaling, which facilitates SSC differentiation and migration [66, 67]. Notably, MAPK is downstream of LM332-α6β4 integrin in hemidesmosomes and LM332-α3β1 integrin in FAs. However, the activation of MAPK by α3β1-LM332 occurs independently of RAS/SHC, as evidenced in studies involving α6β4-LM332 [59, 67]. MAPK activation via RAS, induced by α6β4-mediated anchorage, promotes cell cycle progression in response to mitogenic signals [59]. Conversely, the MAPK cascade, downstream of α3β1-mediated adhesion, plays a critical role in preserving the SSC pool [66, 68]. Moreover, the capacity of α3β1-LM332 to sustain the SSC compartment through MAPK activation may be associated with its downstream target gene, p53, which preserves the proliferative potential of SSCs and interrupts the cell cycle [69]. The p53 homolog p51/p63 enhances the transcription of α3β1 and α6β4 integrins in SSCs, supporting SC preservation by ensuring proper BM attachment and inhibiting Notch1 activity [70, 71]. Nonetheless, it is still uncertain whether MAPK activation by α3β1-LM332 plays a role in sustaining SSC immaturity via p53 or through other distinct downstream effectors. Interestingly, the activity of α3β1 integrin can be negatively modulated by MAPK signaling triggered by EGF or by α6β4 integrin [72]. This negative regulatory mechanism may be essential for preventing SSC hyperproliferation.

The interaction between LM332 and α3β1 integrin at FA sites promotes SSC migration. FAs regulate cell movement by anchoring to the actin cytoskeleton, enabling dynamic interactions essential for motility [52]. The binding of LM332 to α3β1 integrin activates FAK autophosphorylation, which then facilitates its interaction with the Src-homology 2 (SH2) domain of SRC [73]. The recruited SRC phosphorylates other tyrosine residues on FAK, activating the RAC1 downstream pathway, which drives cellular polarization and initiates cell spreading [67, 74]. The engagement of LM332 with α3β1 integrin induces actin cytoskeleton rearrangement and enhances cell movement by preferentially activating RAC1 and CDC42 (Cell division cycle 42), while concurrently weakening RAS homolog family member A (RHOA)/ Rho-associated protein kinase (ROCK) [75, 76]. In addition, inhibition of RHOA and ROCK activity correlates with reduced assembly and stabilization of FAs, reducing cell–matrix attachment and facilitating cell motility [77].

##### LM332 and LM511: antagonistic roles during HF cycle

LM511 is well recognized for its primary role in hair growth and maintenance of HFSC populations, although its functions in IFESCs have been less explored. During the normal HF cycle, LM332 antagonizes LM511-driven hair growth. LM511 encourages the proliferation of HFSCs stimulated by transforming growth factor (TGF)-β2, whereas LM332 inhibits the Wingless-related integration site (Wnt)/β-catenin pathway-induced differentiation of HFSCs [39]. Epidermal progenitor cell (EPC) adhesion to LM511 triggers SMAD2 (SMAD family member 2) phosphorylation, upregulating TGF-β expression without affecting β-catenin stability or Wnt target gene expression [39]. In contrast, adhesion of EPCs to LM332 suppresses β-catenin levels and downregulates Wnt target gene expression, without affecting TGF-β signaling [39]. Normally, a balanced gradient of LM332 and LM511 expression is maintained, with lower LM332 and higher LM511 levels around the bulge niche and hair germ, and the reverse around the basal IFE. This gradient is crucial for sustaining HFSC and IFSC homeostasis. Indeed, reduced levels of LM332 and elevated expression of LM511 enhance EPC assembly into LM511, accompanied by the unrestrained upregulation of Wnt and TGF-β signaling in EPCs [39]. This imbalance in the LM332/LM511 ratio occurs in the absence of actin-binding regulatory protein ILK, which is crucial for laminin-integrin interactions in FA complexes [78]. Accordingly, ILK-depleted mice exhibit hyperproliferation and elevated Wnt gene expression in IFESCs, increasing the risk of carcinogenesis and leading to a loss of quiescent SCs [39]. However, the exact contribution of ILK in modulating the deposition of LM332 and LM511, as well as its interaction with related signaling pathways during HF cycles, remains unclear. Furthermore, LM511 inhibits the proliferation and motility of basal keratinocytes, without significantly affecting their differentiation [79]. The absence of LM511 in the epidermal BM leads to a hyperproliferative phenotype in basal keratinocytes [79]. Moreover, LM511 supports basal cells by sustaining stem and progenitor cells, including melanoma-associated chondroitin sulphate proteoglycan (MCSP) and keratin 15 (K15)-positive cells [80].

b. Laminin-integrin interaction in skin CSC niche

While LMs and integrins are crucial for maintaining the SSC niche, they have also been implicated in tumorigenesis and invasiveness of skin cancers. LM332 overexpression at the invasive edges of SCCs is associated with poor prognosis [81, 82]. Numerous studies demonstrate that α3β1 or α6β4 integrins are indispensable for the tumorigenesis and invasiveness of SCC, BCC, and melanoma [83–88]. Depletion of α3β1 [87–89] or α6β4 integrin [84] in the epidermis reduces both cutaneous SCC size and incidence, whereas its overexpression is associated with poor prognosis [86, 87]. These observations may be attributed to the abnormal forms and localization of LM332 and its integrin receptors in the CSC niche.

##### LM332 maturation disorder: abnormal variants and malignant behavior

The promotive role of LM332 and integrins in the CSC niche is attributed to disruptions in the proteolytic processing of LM332. Disorders in the maturation process of the LM332 precursor induce dysfunctional variants and malignant behavior in LM332-integrin interactions [90, 91]. Physiologically, LM332 undergoes maturation, during which smaller β3 LG4-5 and γ2 EGF-like fragments are released and removed from the ECM in the BM [47]. However, LM332 appears as an independent biologically active α3 LG4–5 or γ2 EGF-like fragment, or contains full-length α3 in the BM, as reported in the microenvironment of aggressive forms of melanoma, SCC, and BCC [92–94]. These abnormal LM332 patterns do not attach well to the ECM, resulting in the breakdown of the BM assembly by destabilizing hemidesmosomes [93].

Abnormal variants of LM332 impair normal function in SSC development, promoting malignant cell behavior. The cleaved component LM332-γ2, comprising EGF-like fragments, is a distinctive marker of metastatic potential of tumors. This recognition is attributed to its prominent expression as a monomer at invasive tumor borders and its increased prevalence in more aggressive subtypes of BCC, SCC, and melanoma [92–96]. Overexpression of LM332-γ2 fragments in skin cancer has been associated with several MMPs, including MMP-2 [94], MMP-7 [97, 98], MMP-13 [99], MMP-19 [100], and membrane-type 1 (MT1)-MMP [94]. Additionally, significant co-localization of these MMPs with LM332-γ2 chains was observed at the invasive margins of skin tumor cells [98, 100]. Approximately 96.2% of BCC cases (50 out of 52) were positive for LM332-γ2, exhibiting a prominently diffuse pattern in the cytoplasm of infiltrating tumor cells. Furthermore, aggressive BCC demonstrated significantly higher levels of LM332-γ2 compared to non-aggressive BCC [96]. In addition, the inhibition of LM332-γ2 substantially decreased the migratory potential of SCC cells in vitro [92]. The supportive role of monomeric LM332-γ2 in skin cancer may be attributed to its EGF-like chain, which activates EGF/MAPK/ERK signaling pathways upon interacting with EGFR in cancer cells [55]. The resultant disassembly of hemidesmosomes, induced by tyrosine/serine phosphorylation of the β4 tail, facilitates the migration and scattering of tumor cells [56, 57]. Hyperactivation of oncogenic RAS, stimulated by the α6β4 integrin-LM332 in an EGFR-dependent manner, is observed in SCC progression and invasion [84]. Additional proteolytic cleavage of the γ2 domain has been proposed to inhibit the deposition and integration of LM332 into the ECM of the BM [51]. However, further research is required to fully understand the nature of this association.

A distinct abnormal form of LM332, either a full-length α3 chain or a monomeric LG4–5 chain, is proposed to contribute to skin cancer invasion. The LG4–5 segment, cleaved from the LM332 precursor on the α3 subunit and absent in the normal epidermis, is exclusively found in tumor tissue and facilitates the formation and progression of SCC [101]. Moderate to strong levels of α3 LG4–5 segments were found in 75% of 75 cutaneous SCC tumors and 56 non-cutaneous SCC tumors [101]. The LM332 harboring the full-length α3 chain exhibits a higher affinity for FA components, such as α3β1 integrin and syndecan-1, compared to α6β4 [102, 103]. Consequently, a transition in the main adhesion structure for the attachment of basal epidermal cells to the BM niche, evolving from reliance on hemidesmosome-dependent regulation to FA-dependent mediation, is observed [102]. Moreover, the activation of the MAPK/ERK/IL-1β pathway by α3 LG4 interaction with syndecan promotes the expression of MMP-1 [104] and MMP-9 [105]. MMP-1 and MMP-9 activity in invasive SCC, mediated by MAPK and PI3K pathways, necessitates the presence of LM332 containing α3 LG4–5 [101]. Notably, these MMPs are capable of cleaving LM332, releasing the LM332-γ2 fragment, which contributes to the progression of BCC, SCC, and melanoma [94–96]. As a result, LM332 α3 LG4–5 may facilitate and amplify the release of LM332-γ2, further driving tumor growth. However, the direct interactions of these altered LM332 fragments in the skin CSC niche are yet to be fully investigated. Future investigation into these interactions may offer critical understanding of the mechanisms underlying skin CSC resistance.

##### Abnormal interactions of aberrant LM332 variants with integrins

The aberrant variants of LM332 interfere with the interaction between LM332 and integrins. Unprocessed LM332 preferentially attaches to integrin α3β1 over α6β4, which triggers FAK phosphorylation and activates the RAS/MAPK pathway. This cascade subsequently enhances RAC1- and CDC42-mediated cell migration [103, 106]. The clustering of unprocessed LM332 is predominantly localized at the FA sites on the cell membrane, potentially enhancing FA formation through efficient binding to α3β1 [102]. This contrasts with the typical form of LM332, which is predominantly recruited to α6β4 sites. The atypical interaction redefines the role of LM and integrin, shifting from the maintenance of cell–matrix adhesion to acting as a cell signaling hub in the skin CSC niche, which facilitates BM disruption and accelerates cell migration. Furthermore, SCC tumors exhibit extensive cell death, along with markedly impaired proliferation and invasion, in the absence of LM332 α3 LG4–5. These defects are attributed to inactive PI3K/AKT signaling and reduced levels of MMPs, such as MMP-1 and MMP-9, following specific inhibition of the α3 LG4–5 domain [101].

Integrin α6β4 and integrin α3β1, which exhibit ectopic expression in the epidermis, can contribute to dysregulated signaling in skin cancer progression, independent of their LM ligands. Rudimentary hemidesmosomes are critical early factors that promote skin tumor cell movement and invasion [102]. Disruption of integrin-hemidesmosome interactions subsequently promotes malignant transformation within the SSC niche. Integrin α6β4 shifts from primarily facilitating cell–matrix adhesion to interacting with signaling proteins when not confined to hemidesmosomes. Altered integrin expression, characterized by abnormal basal or suprabasal expression instead of its typical restriction to the epidermal basal layer, contributes to the progression and invasion of skin cancers [107–110]. Suprabasal integrin α6β4 expression increases throughout SCC progression, with elevated levels associated with early relapse [109, 111]. Additionally, α6β4 integrin receptors redistribute diffusely on cell membranes in BCC and SCC, contrasting with their typical localization in the basal epidermal cells [108, 112]. In the normal epidermis, integrin α3β1 is primarily localized to the lateral membranes of basal SSCs. In contrast, integrin α3β1 is aberrantly expressed throughout the epidermis in SCC and BCC tumors, with a higher concentration on the basal membrane of basal keratinocytes [112, 113]. Notably, epithelial (E)-cadherin-mediated adhesion and PI3K activity are essential for the suprabasal activity of α6β4 integrin in SCC formation, thereby facilitating TGF-β-mediated epidermal hyperproliferation [114]. Additionally, the inflammatory milieu established by suprabasal α3β1 integrin through the MAPK/ERK pathway induces the unrestrained hyperproliferation of keratinocytes [115, 116].

Moreover, non-ligand-bound integrins can initiate oncogenic signaling pathways in the CSC niche. Signaling initiated by non-ligand integrins is contingent on their activation mechanism and the interacting molecules involved, which can generate distinct responses in SSCs and CSCs. Integrin α3β1, independent of its binding to LM332, engages FAK/SRC signaling and activates the downstream AKT/STAT3 cascade in basal keratinocytes, mediated through its membrane-associated protein, CD151 [117–119]. This mechanistic pathway enhances basal cell proliferation mediated by cyclin D [120, 121]. Simultaneously, the integrin α3β1-CD151 interaction at the cell–cell junctions of suprabasal keratinocytes activates AKT and STAT3 signaling, conferencing protection to differentiating cells by preventing apoptosis [117]. Subsequently, the retention of proliferating and differentiating cells due to impaired terminal differentiation facilitates tumor outgrowth and skin carcinogenesis. In contrast, the absence of integrin α3β1 diminishes FAK/SRC activation and attenuates STAT3/AKT signaling, which in turn expedites the depletion of slow-cycling stem cells and the terminal differentiation of suprabasal keratinocytes, ultimately preventing skin tumorigenesis [117, 122]. On the other hand, in the absence of its LM332 ligand, the interaction between α6β4 integrin and its co-receptor syndecan-1 facilitates the self-association of β4 cytoplasmic tails. Phosphorylated β4 engages Erb-B2 receptor tyrosine kinase 2 (ErbB2), a member of the EGFR kinase family [123]. This process initiates a cascade of Fyn-dependent phosphorylation of β4, which is associated with the disruption of hemidesmosomes and the subsequent activation of downstream PI3K/AKT signaling pathways [123]. The resulting PI3K/AKT signaling cascade elicits actin reorganization, facilitating tumor cell migration [124]. Although certain factors associated with the emergence of unconventional integrins have been identified, the precise mechanisms governing the interaction between ectopic integrin expression and signaling pathways in skin tumorigenesis warrant further investigation.

﻿The activation of integrins α6β4 and α3β1 is essential for the tumorigenic properties of CSCs. α6β4 integrin expression is upregulated in CSCs that also express Transglutaminase 2 (TG2), a multifunctional protein essential for CSC survival in SCC tumors [125]. Binding of integrin α6β4 to TG2 activates FAK/SRC/PI3K, which subsequently triggers phosphoinositide-dependent kinase 1 (PDK1) signaling [126]. PDK1, by inhibiting LATS1 activity, dampens the Hippo pathway [127], promoting nuclear accumulation of YAP1. This nuclear YAP1 then stabilizes ΔNp63α (Delta Np63 alpha), subsequently strengthening CSC stemness and promoting invasion of SCCs. Inhibition of α6β4 disrupts PI3K/PDK1 signaling and decreases ΔNp63α activity, which impairs CSC properties and SCC formation [126]. Knockout of integrin α3β1 in HFSCs residing in the bulge niche significantly impairs papilloma development, a type of skin tumor. In the early stages of tumorigenesis, the onset of epidermal tumors is facilitated by α3β1 in HFSCs through the upregulation of HFSC-derived CCN2 (Cellular communication network factor 2) expression [122]. The significant involvement of common integrins in SSCs in the initiation of skin tumors driven by CSCs has been recognized. However, the precise impact of these integrins on CSCs in the context of skin cancers remains largely unexplored. Investigating the functions of these integrins and their associated signaling pathways in the CSC niche may provide deeper insights into the mechanisms that sustain CSCs within their niche.

#### Collagen IV

- Collagen type IV in SSC niche

Collagen IV, another major BM component, primarily functions as a scaffold that supports the attachment of basal epidermal cells to the BM. Collagen IV not only preserves the structural integrity of the BM but also contributes to its durability [38]. Collagen IV forms intermolecular covalent linkages and tightly aggregates with LM and other BM proteins, generating a stable meshwork that provides the BM with the ability to withstand mechanical forces [50]. Basal SSC attachment to the BM is strengthened by the direct binding of collagen IV to integrin α2β1 on these SCs [46]. Additionally, collagen IV in the BM contributes to SSC maintenance. Epidermal cells rapidly adhering to collagen IV demonstrate significant proliferative potential in vitro and form fully differentiated epidermis in vivo [46]. Collagen IV is thought to be crucial for maintaining basal keratinocyte proliferation as a target of miR-135b [128]. In the absence of miR-135b, collagen IV expression in the SSC niche increased, whereas miR-135b reintroduction reversed this effect and promoted early keratinocyte differentiation [128]. Moreover, inhibition of miR-135b consistently elevated p63 levels, a recognized marker of SSCs [129], and enhanced proliferation in normal keratinocytes [128]. These findings underscore that restoring collagen IV in the BM may be essential for SSC proliferation and preservation. This aligns with the role of collagen IV in maintaining BM integrity, which supports SSCs within their niche and sustains their stemness properties [38]. Additional research is needed to uncover the mechanisms driving the interaction between collagen IV and miR-135b and their roles in sustaining the quiescent state of normal SSCs.

b. Collagen type IV in skin CSC niche

During skin cancer progression, the ECM composition surrounding CSCs and tumor cells varies, with collagen being the most abundant matrix protein. Disorganization of collagen IV is a common feature in skin cancers, closely associated with enhanced tumor invasion and progression. In aggressive skin cancers, such as melanoma and SCC, structural disintegration of collagen IV and the absence of an intact BM are often prominent features [112, 130–132]. During epithelial–mesenchymal transition (EMT), a key process in cancer progression, MMP-1 and MMP-2 secretion is significantly upregulated, leading to collagen IV fragmentation and destruction of the BM niche in invasive skin cancers [131]. Furthermore, the degradation of collagen IV in skin cancer may correlate with the aberrant expression of its regulatory factors. Notably, miR-135b, which inhibits collagen IV expression in normal SSCs [128], is overexpressed in human melanoma and aggressive SCCs. This microRNA promotes tumor growth by targeting LATS2 (Large tumor suppressor kinase 2) [133] and enhances invasiveness by inhibiting LZTS1 (Leucine zipper tumor suppressor 1) [134]. In addition, degradation of collagen IV in melanoma and SCCs is frequently observed [112, 130–132], suggesting a potential relationship between miR-135b and collagen IV expression in skin cancers. Therefore, targeting miR-135b may provide a novel approach for restoring BM integrity and inhibiting skin cancer progression.

When collagen IV is cleaved proteolytically, it unveils hidden binding sites that may influence integrin specificity and alter its cellular functions. Proteolytic cleavage of collagen IV denatures its triple helical structure by MMPs such as MMP-2, revealing a cryptic site that is typically obscured [135]. This shift in structure alters the integrin binding specificity from β1 integrin to αvβ3 integrin, which promotes angiogenesis in melanoma [136]. Thus, this alteration in integrin-mediated interactions may serve as a key regulatory mechanism for activating critical signaling pathways involved in invasive cellular behavior. However, the αvβ3 integrin inhibits vascular endothelial cell (EC) and melanoma cell proliferation when engaging with the α3 chain of collagen IV [α3(IV)] [136, 137]. Melanoma cell adhesion is enhanced by the coupling of αvβ3 integrin with the α3(IV) chain, which simultaneously reduces cancer spread by suppressing MMP-2 expression [138]. These findings suggest that modulating the α3 chain in the non-collagenous 1 fragment of collagen IV could offer novel strategies for inhibiting angiogenesis and tumor progression in melanoma.

### Adherens junction (AJ)-mediated cell–cell contact niche

#### Adherens junctions (AJ) in SSC niche

Adherens junctions (AJs), formed by cadherin–catenin complexes, are instrumental in maintaining cell-to-cell cohesion and preserving quiescent SSCs. Transmembrane E-cadherin is essential to AJs, facilitating lateral cell attachment through calcium-dependent binding in its extracellular regions [139]. E-cadherin levels are high in AJs of quiescent bulge stem cells. However, during anagen, as HFSCs experience enhanced cell division and decreased FOXC1 (Forkhead box C1) expression, E-cadherin levels drop accordingly [140]. This implies a negative correlation between the junction stability and SC proliferation.

AJs are reinforced by intracellular associations with cytosolic α-, β-, and p120-catenin. α-Catenin connects AJs to the actin filament network, either through direct interaction or through actin-binding molecules such as vinculin [141]. By stabilizing force transduction between actin and α-catenin, vinculin reinforces α-catenin interactions with both actin and β-catenin [141]. β-catenin is both a critical component of AJs and a transcriptional activator in the canonical Wnt pathway [142]. p120-catenin binds to the membrane-proximal portion of cadherins, recruiting microtubules to the cadherin-catenin junction. This interaction reinforces intercellular adhesion by stabilizing the junctional structure and enhancing cytoskeletal connections [143]. p120-catenin regulates basal epidermal cell behavior by modulating the RAS homolog (RHO) GTPase activity. The cytosolic domain of p120-catenin inhibits RHOA activation by binding to p190RHOGAP (Rho GTPase activating protein 19), preventing RHO–GDP (guanosine diphosphate) dissociation and its subsequent activity [144, 145]. Integrin-activated RAC recruits p190RHOGAP to p120-catenin in AJs, leading to RHOA inactivation and regulation of cytoskeletal dynamics [144]. This interaction partly elucidates the crosstalk between integrins and the RAC inhibition of RHOA in FAs.

AJ components are critical for maintaining the SSC pool and facilitating differentiation in response to mechanical cues. In proliferating basal epidermal cells, AJs extend beyond the lateral sides to encompass the cortical surface, functioning as a crucial cell–cell interface. In differentiating cells, AJs undergo remodeling, marked by a reduction in E-cadherin expression, which decreases adhesion forces and cortical tension in the early stages of differentiation [146]. This reduction in cell interface tension and cell–matrix contact enhances cell polarity and promotes delamination of basal keratinocytes. Upon detachment from the BM, differentiated keratinocytes lose cell–matrix adhesion, including FAs, while concurrently reorganizing and potentially enhancing E-cadherin-mediated adhesion. This transition results in an elevated cortical tension state, which is correlated with a decrease in actomyosin contractility [146, 147]. Thus, the re-establishment of AJs stabilizes the positioning of the suprabasal delaminated keratinocytes. Correspondingly, E-cadherin is expressed in basal SSCs, and its expression increases in the suprabasal layers [147].

The RHO family of small GTPases regulates AJ remodeling through modulation of actin dynamics and junctional integrity [148]. Activated RAC1 specifically disrupts cadherin receptor localization without affecting integrins, resulting in disassembly of cadherin-mediated contacts in keratinocytes [148], possibly by inducing E-cadherin endocytosis [149]. These observations align with the initiation of SSC differentiation, during which α3β1 integrin-LM332 ligation in FAs activates RAC1-mediated cell migration [67, 74]. This process coincides with actin polymerization and reduction of cell–cell AJs [149]. Further research is required to clarify how integrin and cadherin adhesion crosstalk regulates SSC behavior during epidermal regeneration.

Cadherins serve as mechanical sensors, actively responding to mechanical signals from both the extracellular and intracellular mechanical signals. Through interactions with their intracellularly bound proteins, cadherins modulate actomyosin connections and signaling pathways, thereby facilitating SSC adaptation to sustained mechanical stimuli [64]. Specifically, E-cadherin–α-catenin (Eα) complexes maintain the quiescent SSC population by inhibiting core signaling pathways involved in cell cycle activation, such as Wnt/β-catenin and YAP/TAZ pathways. Mechanical strain activates nuclear YAP and β-catenin, which enables quiescent SSCs to rapidly enter the cell cycle. This response relies on E-cadherin-mediated adhesion, which facilitates proliferation under mechanical stress [150]. In keratinocytes, tensile force applied to E-cadherin-based contacts inhibits cell proliferation by impairing the translocation of β-catenin and YAP to the nucleus [151]. E-cadherin stabilizes β-catenin, a crucial transcriptional co-activator of the lymphoid enhancer-binding factor 1 (LEF1)/ T-cell factor (TCF), by binding to it intracellularly and releasing it when necessary [142]. This interaction prevents β-catenin from entering the nucleus and inhibits transcriptional β-catenin/LEF1 activity, thereby regulating Wnt signaling pathway. Conversely, destabilization of the cadherin complex releases β-catenin, which initiates Wnt signaling pathway and promotes SSC terminal differentiation [142].

α-catenin, a force-responsive component of AJs, preserves quiescent SSCs by maintaining AJ integrity and modulating intracellular YAP/TAZ signaling [141, 152, 153]. The establishment of an intercellular bridge, which connects cytoskeletal actin filaments between adjacent cells via AJs, generates tensile forces across these junctions. Sequentially, tension-dependent conformational changes in α-catenin facilitate the exposure of previously concealed domains [141]. This α-catenin variant, in conjunction with vinculin, is thought to locally reorganize actomyosin, reinforcing AJs and maintaining α-catenin in the extended conformation [141]. This conformation stabilizes α-catenin through its association with actin, effectively sequestering YAP1 from α-catenin [152]. Interestingly, an intermediate force generated during actomyosin binding at the AJ cell–cell junctions stabilizes Eα complexes. However, excessive force causes vinculin to dissociate from the α-catenin-actin complex, shifting α-catenin to an inhibited state and liberating YAP1 [141]. By restricting YAP nuclear translocation, α-catenin serves as both an upstream modulator of YAP and an anti-oncogenic factor in the skin, ultimately inhibiting SC expansion [153]. Additionally, α-catenin indirectly regulates YAP/TAZ activity by interacting with protein 14–3-3, which retains YAP1 in cytoplasm [153]. The E-cadherin-α-catenin-β-catenin complex can phosphorylate the Ser127 residue of YAP independently of the Hippo kinase cascades (mammalian sterile20-like 1 [MST1/2]-LATS1/2), thereby exposing its binding site for 14–3-3 proteins [152, 153]. Another alternative non-canonical Hippo pathway mechanism through which α-catenin regulates YAP/TAZ involves the suppression of β4 integrin-mediated SRC activation [154]. In the absence of α-catenin, β4 integrin-mediated SRC becomes hyperactive, promoting YAP1/ TEA domain transcription factors (TEAD) transcriptional activity [154]. Therefore, α-catenin exerts an inhibitory effect on SSC proliferation by directly or indirectly downregulating YAP1.

#### Adherens junctions (AJ) in skin CSC niche

Disruption of AJ integrity in skin epidermal cells, including impaired function or deficiency of E-cadherin or α-catenin, can trigger uncontrolled cell growth and tumorigenesis [153, 155, 156]. Skin cancers, particularly aggressive variants with poor prognosis, frequently exhibit reduced E-cadherin and α-catenin expression [155–159]. The expansion of proliferating SOX9-expressing stem cells occurs following inducible depletion of α-catenin in HFSCs, culminating in SCC tumor development in vivo [152].

The E-cadherin-α-catenin complex performs its oncosuppressive function by regulating multiple mechanisms, including inhibition of β-catenin signaling [157], YAP/TAZ signaling [150, 152, 153], FAK–SRC signaling [160], and the nuclear factor-kappa B (NF-κB) pathway [155]. The elevated expression of neural (N)-cadherin adhesion and the downregulation of E-cadherin, which trigger EMT, coincide with the release of AJ-mediated inhibition of these signaling pathways. In melanoma, the abundance of cytoplasmic and nuclear β-catenin results from the downregulation of E-cadherin, promoting tumorigenic signaling, whereas in normal SSCs, E-cadherin sequesters β-catenin [157]. Moreover, SRC activates N-cadherin phosphorylation, disrupting the β-catenin-cadherin binding and promoting β-catenin accumulation in the cytoplasm and nucleus during melanoma cell transendothelial migration [161]. The increased transcriptional activity of nuclear β-catenin subsequently stimulates the upregulation of target genes essential for the progression and metastasis of skin cancers. These targets are *c-MYC*, *VEGF* (Vascular endothelial growth factor), *TWIST-1* (Twist-related protein-1), *Wnt5a* (Wnt family member 5a), *SOX9*, and *MMPs* [162, 163]. Moreover, down-regulation of α-catenin correlates with increased nuclear phosphorylated NF-κB and STAT3, inducing inflammatory gene expression and aligning with typical features of human skin SCCs [155]. Additionally, the YAP/TAZ signaling pathway involved in skin cancer progression evades AJ surveillance upon α-catenin reduction. Dysregulation of three independent pathways by which E-cadherin and α-catenin mediate YAP/TAZ activity has been observed during skin oncogenic transformation. The resultant increase in nuclear YAP/TAZ localization can be attributed to impaired sequestration of YAP/TAZ by α-catenin or its effector 14–3-3 protein [152]. Enhanced YAP/TAZ transcriptional activity, induced by β4 integrin-mediated SRC activation, may also contribute to the increased α-catenin-mediated YAP/TAZ activity [154].

Hyperproliferation and tumorigenesis in the skin are induced by p120-catenin loss, similar to the effects of α-catenin and E-cadherin depletion. E-cadherin loss disrupts the tumor suppressive role of p120-catenin, promoting cellular proliferation by enhancing NF-κB-mediated inflammation and aberrant RHOA hyperactivity in SCC [164]. In melanoma, RAS/MAPK signaling activates p90 ribosomal S6 kinases (RSKs), which phosphorylate and reorganize p120-catenin, leading to a direct disruption of AJ integrity [165]. Additionally, the reduction of cadherin-mediated adhesion may promote cell migration by activating RHO GTPases via p120-catenin reduction [166]. However, the exact mechanisms and direct interactions involved in these processes in skin CSC niche remain unclear.

Loss of E-cadherin coupled with upregulation of N-cadherin defines the ‘cadherin switch’, a critical driver of EMT and skin cancer metastasis. This shift in adhesive properties correlates with more aggressive behavior and progression of advanced-stage SCC [167], BCC [156, 168], and melanoma [169]. For instance, E-cadherin loss stabilizes c-JUN protein and upregulates target genes [170], such as MMP-9, MMP-14 and MMP-2 [171, 172], key factors in driving melanoma metastasis. N-cadherin on melanoma cell surfaces enables selective binding to tumor-associated cells, such as vascular ECs and cancer-associated fibroblasts (CAFs), which naturally express N-cadherin [169, 173, 174]. The intercellular connections formed by N-cadherin molecules are less stable than those formed by E-cadherin molecules, leading to increased motility in N-cadherin-expressing cells. Additionally, N-cadherin enhances melanoma cell persistence by activating the AKT pathway, thereby preventing apoptosis [175]. Consequently, skin tumor cells acquire a mesenchymal phenotype during EMT, facilitating invasion into the dermis and promoting metastasis [173, 176].

Cadherin switching is triggered by various mechanisms in skin cancer progression, notably through transcriptional regulation of cadherin gene expression. Microenvironmental factors from tumor or stromal cells such as tumor-associated macrophages (TAMs) [177], CAFs [178], including fibroblast growth factors (FGF), TGF-β, EGF, and hepatocyte growth factor (HGF), contribute to cadherin switching [179, 180]. Secreted factors and oncogenic pathways, including PTEN/PI3K and JAK/STAT3 activate EMT-related regulators such as Snail family transcriptional repressor 2 (SLUG), Zinc finger E-box binding homeobox (ZEB) 1/2, Snail family transcriptional repressor (SNAIL), TWIST1, Smad interacting protein (SIP) 1 and Transcription factor 3 (TCF3) [176, 181, 182]. These transcriptional regulators enhance N-cadherin expression while downregulating E-cadherin in skin cancers [168, 179, 183–186]. In particular, Snail-induced cylindromatosis lysine 63 deubiquitinase (CYLD) loss in melanoma promotes B cell lymphoma 3 (BCL3) ubiquitination, inducing N-cadherin expression [187]. TWIST further facilitates EMT by directly repressing E-cadherin transcription through binding to the E-cadherin promoter at the E-box, or indirectly by activating SNAIL [176, 188].

Signaling pathways such as Notch, Wnt/β-catenin, Shh/GLI (Glioma-associated oncogene homolog), PTEN/PI3K, and FAK/SRC are essential regulatory components for the conversion of E-cadherin to N-cadherin during cancer development. Activation of Notch1 enhances N-cadherin expression, thereby facilitating melanoma cell adhesion through N-cadherin interaction [179, 189]. Loss of PTEN activity or hyperactivation of PI3K during melanoma progression correlates with the transition from E-cadherin to N-cadherin, coupled with upregulation of the transcription factors SNAIL and TWIST [176]. Moreover, invasive cutaneous SCCs exhibit markedly reduced E-cadherin expression, accompanied by enhanced activity of the PI3K/AKT signaling pathway [181]. Pharmacological AKT inhibition attenuates EMT progression by decreasing the mesenchymal marker SLUG and restoring E-cadherin, suggesting its potential as a therapeutic target in skin SCC [181]. GLI2, a key mediator of Shh signaling, contributes to E-cadherin downregulation by inducing *SNAIL* in melanoma cells [190]. The p38/NF-κB signaling cascade, activated by free β-catenin accumulating in the cytoplasm following E-cadherin loss, upregulates N-cadherin expression in melanoma cells [191]. The upregulation of β1 integrin-mediated FAK/SRC signaling activates ERK1/2 and STAT3 pathways, which enhance the endocytosis of E-cadherin [29, 192]. Inhibition of the FAK/SRC pathway with SRC inhibitors preserves membranous E-cadherin localization and effectively reduces melanoma cell migration [192, 193]. Despite the proposed mechanisms underlying EMT, the precise regulatory pathways driving the cadherin switch in the skin CSC niche remain unclear.

### Cellular compartments in SSC and CSC niche

In addition to the epidermis’s role in maintaining skin homeostasis, the dermis is a multifaceted layer of skin that provides structural support through its ECM and houses a variety of cells, including immune cells, fibroblasts, adipocytes, and endothelial cells. These cellular components play a significant role in regulating SSC behavior. The interaction between the cellular niche and SSCs is particularly important for HF development and epidermal regeneration [194]. These cells undergo dynamic changes throughout the hair growth cycle, responding to signals from the epidermis in both normal and pathological conditions, such as wound healing.

The roles of CSCs in self-renewal, tumor maintenance, and resistance to therapy are shaped by their interactions with surrounding cells, including immune cells, fibroblasts, adipocytes, and endothelial cells. Molecular signals secreted by CSCs in SCC, BCC, and melanoma can drive a malignant phenotype in adjacent cells, sustaining the TME. In turn, the surrounding cellular niche supports tumor survival, aggressiveness, and treatment resistance.

#### Fibroblasts

- Fibroblasts in SSC niche

Human skin dermis contains heterogeneous fibroblast subpopulations, each with distinct structural and functional characteristics that play a role in regulating SSC fate and HF growth [194, 195]. Apart from supporting HF regeneration, dermal fibroblasts also contribute to the maintenance of BM integrity through the synthesis of ECM proteins and collagen [196, 197].

Dermal fibroblasts play a dual role in promoting and inhibiting HFSC activation, thereby influencing HF development and maintaining the SSC pool. Papillary fibroblasts, located immediately beneath the epidermis, are essential for HF formation. In contrast, reticular fibroblasts, residing in the deeper dermis, primarily contribute to the synthesis of major constitutive components of the ECM [196, 198]. Notably, the composition of the dermal fibroblast population shifts across the different stages of the HF cycle. During telogen, reticular fibroblasts dominate the dermis, while papillary fibroblasts become more prevalent during anagen [198]. Stabilizing β-catenin in papillary fibroblasts prolongs the anagen phase, whereas its stabilization in reticular fibroblasts does not affect HF cycling [199].

Distinct fibroblast subsets exhibit differential responses to signals from SSCs throughout the HF cycle. Activated Wnt/β-catenin signaling in the epidermis increases the number of papillary and reticular fibroblasts [198, 200]. Subsequently, Wnt/β-catenin-mediated enhancement of Shh signaling potentiates fibroblast proliferation in the papillary dermis, promoting HF growth. Epidermal TGF-β signaling, activated through Wnt/β-catenin, drives reticular fibroblasts to orchestrate ECM remodeling [198]. However, when Shh signaling in fibroblasts is suppressed, both papillary and reticular fibroblasts fail to respond to epidermal β-catenin activation [198]. This finding underscores the paramount role of dynamic Shh regulation in Wnt/β-catenin signaling during physiological HF cycling, ensuring the selective accumulation of fibroblasts in the anagen HF while curbing the aberrant hyperproliferation of quiescent HFSCs in the bulge [33]. Meanwhile, paracrine TGF-β1 signaling acts as a trigger for reticular fibroblast proliferation and ECM protein synthesis, promoting collagen maturation [198]. Activation of epidermal Wnt/β-catenin signaling enhances ECM-related gene expression, such as *COL11A1*, in dermal fibroblasts [201]. Furthermore, by functioning as a negative feedback mechanism, dermal fibroblasts help preserve stem cell reservoir. During the telogen phase, these fibroblasts amplify the BMP (Bone morphogenetic protein) signaling pathway by releasing BMP4, which suppresses HFSC activation and preserves the SSC pool [202]. In addition, the TGF-β2 signaling pathway, activated after Shh induction by Wnt/β-catenin, suppresses ECM production, collagen maturation, and fibroblast proliferation in the reticular dermis [198]. This may act as a negative feedback mechanism, preventing ectopic HF formation and excessive ECM protein accumulation. However, the underlying mechanisms of these processes are not fully understood and warrant further investigation.

b. Fibroblasts in skin CSC niche

CAFs are crucial for tumor progression, immune suppression, and treatment resistance in melanoma, SCC, and BCC [203–205]. CAFs, characterized by αSMA (Alpha smooth muscle actin) upregulation, modulate the CSC niche by engaging various signaling pathways, promoting dermal fibroblast expansion and increasing ECM stiffness [206]. The secretion of MMPs, such as MMP-2, MMP-11 and MMP-14, by these cells accelerates ECM degradation, promoting cancer cell invasion [203, 204]. Notably, hyperactivation of YAP1 in CAFs enhances tumor cell adhesion to CAFs via N-cadherin. Furthermore, YAP-silenced CAFs fail to activate the PI3K/AKT signaling pathway when attaching to N-cadherin-expressing melanoma cells [207]. In the melanoma microenvironment, N-cadherin-expressing fibroblasts and ECs can produce growth factors such as FGF, IGF-1 (Insulin-like growth factor), HIF (hypoxia-inducible factor), VEGF, and TGF-β. These factors foster MSLC (melanoma stem-like cell) proliferation, contributing to melanoma progression [208].

CAFs are recognized as key factors limiting immunotherapy effectiveness, exhibiting a distinct profile of immunomodulatory proteins and secreted factors that influence both innate and adaptive immune mechanisms against tumors [209]. For instance, CAFs can promote the infiltration and activity of immunosuppressive cells, including TAMs, myeloid-derived suppressor cells (MDSCs), and regulatory T cells (Tregs) in the melanoma microenvironment [209, 210]. Additionally, CAFs can weaken the proliferation, survival, and cytotoxic capabilities of cytotoxic T cells (CTLs) [211, 212] and natural killer (NK) cells [213, 214]. Additionally, therapies targeting CAFs can enhance CTL and NK cell-mediated tumor elimination, while preventing the recruitment of MDSCs and Tregs [210].

T cell dysfunction in melanoma, which contributes to resistance to CTL treatment, is partially influenced by CAFs. Melanoma-derived fibroblasts secrete factors such as C–C motif chemokine ligand 5 (CCL5), CCL2, and C-X-C motif chemokine ligand 12 (CXCL12), which induce immune checkpoint (ICP) ligand expression in melanoma cells, thereby compromising CD8^+^ T cell function [211, 212]. Notably, CAFs can shield tumor cells from immune attack by promoting the elimination of tumor antigen-specific CD8^+^ T cells. Melanoma-derived CAFs engulf antigens and exhibit delayed antigen processing, acquiring features similar to those of antigen-presenting cells (APCs). This enhances their ability to present cross-priming antigens to T cells, thereby distracting and diverting T cell attention from tumor cells [215]. Additionally, the simultaneous upregulation of FAS (Fas cell surface death receptor)/PD-1 (Programmed cell death protein 1) on T cells and FASL (Fas ligand)/PD-L2 (Programmed cell death ligand 2) on CAFs impairs the function and survival of tumor antigen-specific T cells, ultimately facilitating melanoma survival and progression [215]. Conversely, enhanced infiltration of tumor antigen-specific CD8^+^ T cells and reduced tumor volume are observed when the activity of PD-L2 or FASL on CAFs is blocked [215]. Collectively, targeting CAFs could be a strategy to counteract immune evasion in skin cancers.

In addition, CAFs can hinder the efficacy of immunotherapy through multiple mechanisms. Secreted factors like IL-6 and TGF-β from CAFs reduce the expression of major histocompatibility complex (MHC) I and II molecules in melanoma cells, promoting resistance to CTL-based therapies [216, 217]. By disrupting NF-κB and ERK1/2 signaling, melanoma-associated CAFs restrict CTL function, reducing granzyme B secretion and impairing their capacity to kill melanoma cells [218]. Furthermore, CAFs can reduce the effectiveness of anti-PD-1/PD-L1 therapy by concealing PD-1 molecules expressed on melanoma cells. Increased CAF accumulation within melanoma is associated with a reduced success rate of PD-1-targeted treatment [219]. CAF-derived MMP-9 may contribute to resistance against anti-PD-1 therapy by cleaving PD-L1 from melanoma cell surfaces, potentially impairing immune system recognition [220].

CAFs can protect tumor cells from immune destruction by significantly altering the NK cell-dependent anti-tumor immune response. CAFs decreased melanoma cell sensitivity to NK cell-mediated cytotoxicity by secreting high levels of MMPs, such as MMP-1, -2, -3, and -9. These MMPs promote the detachment of MHC class I polypeptide-related sequence A and B (MICA/B), ligands of NKG2D (Natural killer group 2 member D), from the surface of melanoma cells, weakening the NKG2D-mediated cytotoxic response of NK cells against tumor cells [213]. Furthermore, MMP inhibitor restored the presence of MICA/B on the melanoma cell membrane, thereby enhancing their susceptibility to NK cell-mediated destruction [213]. However, blocking MMP activity in CAF-conditioned media did not fully reinstate melanoma cell vulnerability to NK cell assault, indicating that MMPs are not the only contributors involved [213]. Further investigation is required to ascertain whether MMP inhibitors can augment NK cell-mediated tumor surveillance or whether additional CAF-derived factors contribute to facilitating cancer resistance. Additionally, CAFs secrete prostaglandin E2, which inhibits the upregulation of natural killer cell p44 (NKp44) and NKp30 expression on NK cells, thereby impeding NK cell activation [214]. These receptors enhance NK cell cytotoxicity by identifying and attaching to their specific ligands on tumor cells. Consequently, NK cells with low expression of these receptors impair their capacity to effectively target and eliminate tumor cells.

CAFs contribute to the progression of SCC by inhibiting anti-tumor immunity and facilitating tumor invasion via ECM remodeling. These fibroblasts are scarce in pre-cancerous lesions, but their abundance increases in established SCC tumors, emphasizing their involvement in tumor advancement [204]. Recurrent SCC shows an association between CAFs and upregulation of EMT markers, including TGF-β1 and vimentin. This is coupled with immunosuppressive traits in recurrent SCC, characterized by reduced inflammation and increased exhausted CD8^+^ T cells [221]. CAFs in relapsed SCC exhibit oncogenic properties, including impaired phagocytosis, reduced inflammation, and increased angiogenic potential, which collectively drive tumor proliferation and metastasis [221].

The interaction between CAFs and CSCs in the TME has been observed, but the precise role of CAFs in the CSC niche remains unclear. Melanoma cells attract dermal fibroblasts to the TME, inducing SOX2 expression in them and driving their transformation into CAFs [222]. Additionally, melanoma cells persuade fibroblasts to adopt a CAF phenotype by secreting soluble factors such as platelet-derived growth factor (PDGF), FGF, and TGF-β [217]. A heterogeneous population of fibroblasts, including cells with CAF-like properties, presented when dermal fibroblasts were cultured with melanoma spheroids [223]. CAFs in melanoma stroma contribute significantly to tumor progression by interacting directly with tumor cells and secreting a variety of factors. These include ECM proteins like collagen, MMPs (MMP-2, -9, and -13), angiogenesis-related factors (VEGF), growth factors (TGF-β, HGF), and chemokines such as CXCL-8 and IL-6, which affect tumor cell growth, metastatic potential, drug resistance, and CSC self-renewal [212, 223–225].

Moreover, CAFs have been demonstrated to modulate CSC behavior. CD44-expressing CAFs support the preservation of MSLC stemness and self-renewal capabilities through direct cellular interactions. However, the exact mechanistic pathways governing these interactions between CAFs and CSCs remain elusive. Interestingly, CD44^+^ CAFs can sustain MSLC survival in avascular environments [226]. Moreover, the abundance of CD44^+^ CAFs in tumor tissues was exacerbated following angiogenesis suppression using anti-VEGF neutralizing antibodies [226]. A synergistic approach targeting both CAFs and angiogenesis may potentiate therapeutic efficacy against malignant melanoma. These findings indicate that CAFs are implicated in the drug resistance mechanisms of malignant melanoma, particularly in relation to CSCs.

Although CSCs and adjacent CAFs in the tumor stroma interact pathologically, the exact molecular and biological processes remain poorly understood. Furthermore, key signaling pathways, such as the YAP/TAZ signaling pathway, in regulating normal SSC and CSC development, are associated with drug resistance when activated in CAFs. Thus, investigating the interactions between CSCs and CAFs could offer valuable insights for overcoming resistance in skin cancer therapies.

#### Immune cells

- Immune cells in SSC niche

Immune cells in the epidermis and dermis, including innate and memory cells, are crucial for skin function and epidermal homeostasis. Although the specific molecules mediating the interactions between SSCs and immune cells are still being identified, it is evident that these cells mutually regulate their behavior and functions.

Dendritic epidermal T cells (DETCs; γδ T cells) and Langerhans cells are found in the epidermis, functioning as T cells and APCs, respectively [227]. Despite their presence in the IFE and outer root sheath of HFs, the exact role these immune cells play in regulating SSC functions remains largely unknown [227, 228]. Their influence on HFSCs has been observed particularly in wound healing scenarios. Activated DETCs stimulate HFSC proliferation for HF growth and the healing process [229].

Immune cells residing in the dermis, including macrophages, CD4^+^ T cells, and Tregs, contribute to SSC development. These cells influence the natural process of HF regeneration by directly affecting the HFSC behavior [230, 231]. Immune cells influence HFSC differentiation and activation through signaling pathways like Wnt/β-catenin, JAK/STAT, and JAGGED1/Notch within the SSC niche [232]. Their distribution and function vary during hair development and growth phases [231, 232].

In the dermis, resident Tregs, especially CD4^+^ Tregs, are the most prevalent immune cells, constituting approximately 20% of the total resident T cells. Tregs are predominantly localized in the vicinity of HFs, enabling dynamic interactions with bulge-derived HFSCs [233]. When Tregs are depleted, there is a significant reduction in HF regeneration, emphasizing their critical function in modulating stem cell proliferation and maturation [231]. The number and functional state of Tregs in the skin are tightly associated with distinct stages of the HF cycle. In the telogen phase, the abundance and activation of local Tregs drive the shift to anagen by promoting HFSC proliferation and differentiation. In contrast, Treg numbers decline throughout the anagen period [231]. Most skin-resident Tregs, which express high levels of JAGGED1, rely on the Notch signaling pathway to activate HFSCs and modulate efficient hair regeneration [231, 234]. Absence of JAGGED1 on Tregs results in a substantial decrease in HFSC population and downregulation of critical epidermal differentiation genes, such as *SOX7*, *SOX4*, and *CCND1* (Cyclin D1) [231]. The depletion of CD4^+^CD25^+^FOXP3^+^ Tregs hampers CD34^+^ HFSC proliferation, inhibiting their progression to the anagen phase [231]. In addition to HF regeneration, Tregs aid in the regeneration of IFESCs. This role becomes apparent during epidermal repair following injury, where a particular population of Tregs enhances EGFR expression to promote wound healing [235]. Overall, Tregs in the SSC niche primarily carry out non-immune functions, playing a role in supporting both epidermal and HF regeneration.

HFSCs and IFESCs exhibit contrasting immunogenic profiles, facilitating their distinct physiological functions. While HFSCs can evade detection by T cells, IFESCs are recognized and eliminated by T cells promptly. Upon activation, local T cells expand and efficiently kill KRT14^+^ IFESCs, which express high levels of MHC I [236]. In contrast to IFESCs, LGR5^+^ bulge HFSCs exhibit a downregulation of MHC I, which helps them avoid detection and destruction by T cells [236]. Moreover, IFESCs are in close proximity to a dense population of resident immune cells, such as γδ T cells and memory CD8^+^ T cells, which serve as crucial immune effector cells in the initial response to both inflammation and cancer progression [237, 238]. This supports the role of IFSCs and their differentiated cells as the primary defense against pathogens. The highly immunogenic IFESCs act as detectors of inflammatory signals and produce cytokines to sustain immune balance and a healthy physical barrier.

In contrast, the HF region is characterized by immune privilege, which supports the maintenance of SSCs for both epidermal and HF regeneration. The dynamic activity of HFs and their signaling pathways during physical injury and the HF cycle recruit various immune cells. This recruitment increases the risk of immune activation and inflammation, putting HFSCs in the bulge niche at greater vulnerability. Therefore, the attenuated immunogenicity of the HFSC niche ensures the preservation of its population. HFSCs, situated in a Treg-dense niche, express the immunoinhibitory molecule CD200 [239] and resist T-cell destruction, potentially resulting from reduced MHC I expression on their surfaces [236]. In the telogen phase, high levels of MHC I protein are observed across the IFE and isthmus region of the HF, with the bulge being the only area showing low MHC I expression [236]. This reduces their visibility to T cells, along with the high presence of resident Tregs, safeguarding the stem cell reservoir essential for lifelong skin regeneration.

Macrophages are also crucial for regulating SSC activity. HFSC proliferation and hair growth are enhanced due to the depletion of TREM2^+^ macrophages, which are present around the HF during the quiescent phase. These macrophages maintain HFSC dormancy during the telogen phase, preserving HF quiescence [240]. Removal of skin-resident macrophages triggers premature HF growth, driven by the Wnt/β-catenin pathway in HFSCs [230]. Additionally, the increase in macrophages expressing Wnt10a and Wnt7b coincides with the activation of bulge HFSCs before the onset of anagen [230]. Thus, under normal conditions, macrophages act as key regulators of HFSC activity.

b. Immune cells in the skin CSC niche

Recent research and current immunotherapy approaches have shown the clinical potential of targeting immune cells within the CSC niche for treating skin cancers, particularly melanoma [241–243] and SCC [244]. However, resistance, both primary and acquired, is commonly observed in advanced melanoma and SCC patients treated with immunotherapies like CTLs and ICP inhibitors [245, 246]. This resistance is often associated with CSCs and other components of the CSC niche.

##### Decreased function of anti-tumor immune cells in CSC niche

The immune cell niche typically helps eliminate malignant cells, but in the CSC niche, these responses turn immunosuppressive. The impaired anti-tumor functions of immune cells contribute to tumor cell evasion of immune surveillance by promoting inhibitory receptor expression and pro-inflammatory cytokine secretion in melanoma and SCC [241–243, 247]. CD8^+^ T cells are key effectors in cancer immune surveillance, leveraging their ability to identify and eliminate target cells through T-cell receptor (TCR) recognition of antigens displayed by MHC I molecules [248]. However, despite their cytotoxic potential, CD8^+^ T cells, especially those associated with CSCs, fail to mount a sustained and effective anti-tumor response in refractory melanoma and SCC.

MSLCs evade immune detection by downregulating melanoma-associated antigens (MAAs), preventing effective anti-tumor immune responses [249, 250]. In melanoma, tumor cells typically express MAAs such as gp100, MAGE-A (Melanoma antigen family A), MART-1 (Melanoma antigen recognized by T cells 1), and CTAG1B (Cancer/testis antigen 1B), which attract MAA-specific CTLs for targeted destruction [250]. CD133^+^ CSCs in melanoma, which display elevated levels of MAAs like CTAG1B, are particularly susceptible to recognition by specific T cells [251]. Nevertheless, a distinct subset of MSLCs suppresses MAA expression on their surfaces, thereby maintaining their immune privilege and protecting them from immune attack. Specifically, MSLCs expressing ABCB5 and CD271 demonstrate a marked attenuation in the levels of MAAs such as MART-1, CTAG1B, and MAGE-A [249, 252, 253]. Moreover, CTL therapy can induce melanoma cell dedifferentiation, marked by increased CD271 (NGFR [Nerve growth factor receptor]) expression and reduced MAA levels, including gp100 and MART-1. Such changes contribute to resistance against CTLs and are associated with increased levels of TNF-α, an inflammatory mediator released by activated T cells [254, 255]. Additionally, inhibition of CD271 can reverse melanoma resistance, restoring its vulnerability to T cell-mediated attacks. The presence of the CD271 signature in melanoma cells may predict resistance to anti-PD-1 treatment [256].

MSLCs and their secreted factors impair cytotoxic T cell function. MSLCs expressing ABCB5 significantly reduce or completely lack MHC I expression [249]. Melanoma cells lacking MHC I expression are unable to be recognized by CD8^+^ T cells, impairing the anti-tumor immune response. Even when MAAs are present, the absence of functional MHC I molecules blocks CTL activation, further enabling immune evasion. Moreover, overexpression of PD-1, PD-L1, CD86, and B7-2 in ABCB5^+^ MSLCs suppresses immune function by inducing T cell anergy, recruiting Tregs, and enhancing the secretion of immunosuppressive cytokines, such as IL-10 [249, 257, 258]. On the other hand, ABCB5^+^ MSLCs can release TGF-β family members, including TGFB2 and TGFB3, into the CSC niche, thereby reinforcing immunosuppression through the promotion of Treg activation [249]. However, it remains unresolved whether this resistance is primarily driven by the inflammatory microenvironment, or if other factors within the CSC niche contribute significantly to the amplification of melanoma resistance to CTLs and various immunotherapeutic approaches.

Although T cells heavily infiltrate SCC, the presence of CSCs fosters resistance to T cell immunotherapy, preventing these immune cells from successfully eliminating the tumor in advanced stages of SCC [259, 260]. A subset of CSCs in SCC preferentially evades T cells by upregulating CD80 in response to TGF-β, which promotes T cell exhaustion and contributes to tumor relapse [261]. CD80 overexpression on CSCs within the SCC niche binds to cytotoxic T-lymphocyte antigen 4 (CTLA-4) on T cells, effectively suppressing T cell activity [261]. Moreover, Tregs are implicated in SCC metastasis and may serve as important prognostic markers. The SCC microenvironment, especially in metastatic cases, shows significant accumulation of CD4^+^FOXP3^+^ and CD8^+^FOXP3^+^ Tregs [262–264]. These tumor-infiltrating Tregs suppress the expansion of effector CD4^+^ and CD8^+^ T cells and their secretion of interferon gamma (IFNγ) [259]. However, it remains uncertain whether the elevated presence of Tregs or other immunosuppressive factors is directly associated with CSCs in the SCC microenvironment. Investigating the relationship between CSCs and immune system interactions may provide deeper insights into SCC progression.

##### Increased immunosuppressive cells in CSC niche

In addition to exhausted T cells in resistant tumors, immunosuppressive cells, such as Tregs, TAMs and tumor-associated neutrophils (TANs), are frequently observed and associated with drug resistance in the CSC niche.

**Tumor-associated macrophages (TAMs)**

TAMs, transitioning from M1 to M2 forms, are present in melanoma, SCC, and BCC, contributing to drug resistance and poor clinical outcomes [265–271]. In SCC, IL-10, IL-4, and VEGF-A drive TAM polarization to the M2 type, promoting neovascularization [272]. In BCC, an abundance of M2 macrophages has been linked to more aggressive disease, marked by deeper invasion and elevated microvessel density [268]. Furthermore, co-culturing BCC cell lines with M2 macrophages significantly augments the invasiveness and angiogenic potential of tumor cells [268]. In melanoma, M2 macrophages are primarily found in the early inflammatory infiltrate, whereas M1 macrophages, known for their anti-cancer activity, are present in much smaller numbers within the tumor [273]. Moreover, melanoma cell-derived PD-L1 promotes macrophage polarization toward the TAM phenotype, exacerbating the immunosuppressive landscape of the TME [274]. The secreted products from TAMs contribute to various processes, such as ECM remodeling, angiogenesis, pro-inflammatory cytokine production, and the promotion of local invasion and metastasis in melanoma, SCC, and BCC [266, 268–270, 275]. TAM-secreted factors include a range of chemokines, including IL-2, IL-10, IL-1, TNF-α, IFNγ, angiotensin, cyclooxygenase-2, CCL12, CCL17 and IL-1β. These chemokines recruit immunosuppressive cells, including Tregs, MDSCs, and T-helper 2 (Th2) cells, which are essential for sustaining the immunosuppressive TME [265, 268, 269, 276–280]. TAMs also secrete VEGF and MMPs, such as MMP-9 and MMP-11 [266, 268], which are crucial for tumor dissemination.

TAMs influence the TME by releasing chemokines that recruit immunosuppressive cells and cytokines that stimulate fibroblasts to release additional chemokines. For instance, IL-1β released by TAMs prompts fibroblasts to generate CXCR2 ligands, which are crucial for attracting MDSCs to melanoma tumors [281, 282]. Additionally, the combined use of anti-CD115 antibodies, which significantly reduce TAMs by targeting CSF1R (Colony stimulating factor 1 receptor), and CXCR2 antagonists has demonstrated potent antitumor effects in vivo. This approach not only prevents the recruitment of granulocytic MDSCs via CXCR2 blockade but also depletes TAMs through CSF1R inhibition, thereby disrupting immune evasion mechanisms within the melanoma microenvironment [282].

TAMs contribute to the failure of the immunomodulatory effects of chemotherapeutic agents in skin cancers. Increased TAMs and PD-L1 are associated with melanoma resistance to the single-agent BRAF (Serine/threonine-protein kinase B-raf) inhibitor dabrafenib or in combination with MEK (Mitogen-activated protein kinase) inhibitor trametinib [283]. TAMs are associated with poorer prognosis, likely by facilitating immune evasion in melanoma [273]. Similarly, improvements in treatment are associated with a reduction in TAM infiltration within SCC tumor tissues. Peplomycin enhances cytotoxic T lymphocyte presence and reduces TAM and Treg levels, potentially enhancing immune response and defense against SCC [284]. TAMs in malignant melanoma exhibit elevated PD-L1 expression [285], which impairs macrophage phagocytosis and diminishes their tumoricidal functions [286]. The therapeutic efficacy of cytotoxic agents such as dacarbazine, vincristine, and nimustine hydrochloride relies on their ability to modulate TAM activity, notably through the suppression of PD-L1 expression and a decrease in CCL22 chemokine levels [287]. These drugs, commonly used in adjuvant treatment for advanced melanoma, exert anti-TAM effects as a key aspect of their therapeutic action [287]. Notably, circadian modulation of PD-1 expression on TAMs significantly affects the response to PD-1 inhibitors in melanoma treatment. The therapeutic impact of PD-1 inhibitors is considerably attenuated when TAMs exhibit minimal PD-1 expression on their surface during the daytime, thereby impairing the immune response [288]. Intriguingly, DEC2 (differentiated embryo-chondrocyte expressed gene 2) transcriptionally regulates diurnal PD-1 expression on TAMs by inhibiting the time-dependent activation of *PDCD1* (Programmed cell death 1) through p65. Downregulation of DEC2 in macrophages increases *PDCD1* expression, encoding PD-1, which in turn enhances tumor cell clearance by anti-PD-1 therapy [288]. Therefore, therapeutic interventions that modulate or block PD-L1 expression on TAMs can potentiate anti-tumor immunity.

In malignant skin tumors, CSC niches are intricately regulated by immune cells, particularly TAMs. SCC, originating from oncogenic mutations in IFESCs, exhibits infiltration of TAMs, which secrete VEGF within the CSC niche, thus fostering tumor growth [246]. TAMs can support the survival of CD34-negative tumor-initiating cells and promote melanoma progression [289]. TGF-β released from TAMs and Tregs in the hypoxic melanoma microenvironment induces glucosylceramide synthase (GCS) expression in melanoma cells, crucial for sustaining and expanding MSLCs [290]. Inhibition of GCS effectively eliminates the immunosuppressive functions of Tregs and TAMs [290]. The microphthalmia-associated transcription factor (MITF)-low melanoma, typically characterized by high stemness, produces a CCL2-rich secretome [291], which promotes the recruitment of TAMs. These observations imply that TAMs are crucial in the skin CSC niche and may become potential targets for future molecular therapies. However, the complex interplay between skin CSCs and TAMs, which underpins the maintenance and tumorigenic potential of CSCs, is yet to be fully understood.

**Tumor-associated neutrophils (TANs)**

The interplay between TANs and CSCs fosters sustained viability of CSCs, thereby driving the continued progression of skin cancer. Neutrophils initially act as the first line of defense in early inflammation, promoting immunostimulatory effects. However, they rapidly transform into TANs within the immunosuppressive TME of skin cancers. Melanoma [292, 293] and SCC [294] patients with abundant TAN infiltration often exhibit poorer therapeutic outcomes and advanced disease stages. The secretome of MSLCs, including IL-6, IL-8, GM-CSF (Granulocyte–macrophage colony-stimulating factor), CCL2, and TGF-β, not only recruits neutrophils but also induces their polarization toward the protumor phenotype [292, 295]. The activation of the STAT3/ERK and IL-8/CXCR2/NF-κB pathways in TANs is triggered by exposure to MSLC-conditioned medium [295]. Moreover, MSLC-conditioned medium enhances ROS (Reactive oxygen species) production, cytokine release, MMP-9 secretion, and NET (neutrophil extracellular trap) formation in neutrophils, which collectively bolster the immunosuppressive and protumor functions of TANs. MSLC-activated neutrophils impart unique stem cell characteristics to melanoma cells, enhancing ABCG2 (ATP-binding cassette sub-family G member 2) expression and capacity to form melanospheres [295]. The reciprocal interaction between CSCs and neutrophils in melanoma underscores the need for further research on tumor-promoting TANs in the skin CSC niche to better understand the mechanisms driving resistance in skin cancers.

#### Adipocytes

- Adipocytes in SSC niche

Adipocytes, in conjunction with dermal fibroblasts, are crucial for epidermal integrity and HF cycling regulation. During anagen, intradermal adipose tissue regenerates, increasing the adipose layer thickness and enhancing adipocyte proliferation [296]. Adipocytes and preadipocytes in the skin have contrasting roles in regulating HFSC activity during the physiological hair cycle [297].

Mature adipocytes in the dermis regulate the duration of the hair cycle’s resting phase. Upon the activation of HFSCs for anagen, these adipocytes secrete BMP proteins [298, 299]. As hair growth progresses, the increased in mature adipocytes enhances BMP ligand expression in the dermis [298]. The expression of BMP2 in these adipocytes aligns with telogen phase, promoting HFSC quiescence and preventing overactivation. As the hair cycle shifts from telogen to anagen, preadipocytes release PDGF-α, which activates PDGF signaling in the dermal papilla, thereby stimulating HFSCs [296]. The communication between adipocytes and HFs underscores the critical role of cellular niche in supporting proper SSC development. When adipocyte precursor formation is blocked, the resulting loss of adipocytes disrupts HFSC activation and delays the onset of hair growth [296]. The activation of HFSCs by preadipocytes is regulated by PPARγ (Peroxisome proliferator-activated receptor gamma), an adipogenic transcription factor predominantly expressed in preadipocytes. Inhibiting PPARγ before the onset of anagen disrupts the regrowth of adipose tissue in dermis by disrupting preadipocyte function [296]. Furthermore, PPARγ antagonists did not impact epidermal proliferation or preadipocyte formation but effectively inhibited the development of PPARγ^+^ preadipocytes [296].

The dynamic process of adipogenesis and its impact on HF growth are influenced by epidermal signals, particularly Shh and Wnt/β-catenin signaling [300, 301]. Activation of Wnt/β-catenin in the epidermis stimulates HF growth and regulates adipogenesis, driving adipose tissue expansion and dermal adipocyte differentiation [198, 300]. The precise mechanism is not fully understood, but keratinocytes are thought to release proadipogenic factors like BMP2, BMP6, and IGF2, which promote adipocyte differentiation [300]. The persistence of Wnt/β-catenin activation in the epidermis triggers abnormal HF growth and thickens the adipose tissue layer. This also causes an abnormal accumulation of adipocytes in the deeper dermis and their unusual presence in the superficial dermis, a region in which they are typically absent [300]. Once anagen is initiated, TACs in HFs increase Shh production, promoting the proliferation and differentiation of preadipocytes through PPARγ, and enabling TACs to generate both their progeny and neighboring stromal lineages [301].

The functional roles of adipocytes in the HFSC niche have previously been identified. However, the role of adipocytes in other aspects of SSC development remains unclear. Future studies are required to uncover the molecular pathways through which adipocytes regulate SSC behavior and to determine whether SSCs can modulate adipocyte function.

b. Adipocytes in the skin CSC niche

Although the involvement of cancer-associated adipocytes (CAAs) is well-documented in various cancers, their exact role in the skin CSC niche remains largely unexplored. In melanoma, the adipose tissue surrounding the tumor shows significant macrophage infiltration with TAM-like properties [302]. The influx of macrophages prompts melanoma cells to secrete angiogenic factors like VEGF and pro-inflammatory cytokines such as MCSF (Macrophage colony-stimulating factor) and CCL2 [303]. Considering the spatial proximity of adipose tissue to melanoma and SCC lesions, further investigation is imperative to elucidate their potential interactions and subsequent influence on tumorigenesis and progression [302, 304].

Adipose tissue in the skin cancer niche can influence the anti-cancer immune response. Secreted proteins from adipose tissue-derived macrophages, such as YKL-40 (chitinase-3-like protein 1), have been correlated with poor survival rates in melanoma patients [305, 306]. This may be due to a TAM-related reduction in NK cell accumulation, which promotes pulmonary metastasis in melanoma [307]. Adipocyte-derived TNF-α and IL-6 are positively associated with PD-L1 expression in melanoma [308]. On the other hand, reduced adiponectin levels, commonly downregulated in cancer, foster melanoma progression while hindering the recruitment of tumor-suppressive macrophages to the TME [309].

Despite being abundant in the TME, the role of adipocytes in skin cancers and CSC development remains poorly understood. In cancers such as prostate cancer and breast cancer, adipocytes undergo differentiation into CAAs, exhibiting distinct characteristics from their normal counterparts [310]. These CAAs contribute to tumorigenesis and protect tumor cells from therapeutic treatments. Consequently, exploring the interplay between the skin CSC niche and CAAs is crucial for devising more effective approaches to targeting and eradicating resistant skin cancers.

#### Endothelial cells (ECs)

- Endothelial cells in SSC niche

The epidermis and HFs are avascular and rely on nearby dermal blood vessels to supply nutrients. ECs support HF regeneration by promoting angiogenesis during the anagen phase and undergoing apoptosis in the catagen phase [311]. As HFSCs in the hair germ prepare for activation during the transition from late catagen to telogen, the skin vasculature undergoes morphological changes [311]. It becomes more horizontal, forming a distinct plexus with tightly packed blood vessels beneath the hair germ. This vascular network briefly aligns with the region where HFSC activation occurs during the telogen phase [311]. During the resting phase, EGFL6 (Epidermal growth factor-like domain 6), an angiogenic factor expressed by K15-negative HFSCs above the bulge, may attract ECs to telogen HFs [312]. As activated HFSCs proliferate from telogen to early anagen, the vascular plexus progressively disperses away from the proliferating hair germ [311].

ECs play a role in maintaining the SSC source. When angiogenesis is suppressed, anagen onset is delayed [313]. However, during the late catagen and early telogen phases, an expanded vascular network near the hair germ generates BMP4 signals that sustain HFSC quiescence prior to activation and the initiation of hair growth [311, 314]. Moreover, an enhanced density of the vascular network surrounding the hair germ during the telogen phase is concomitant with extended HFSC quiescence and a delayed transition into the anagen phase [311]. These findings suggest that EC compartments may influence the timing of HFSC awakening from the resting state, ensuring proper skin homeostasis. Further studies are essential to elucidate the interactions between skin ECs and SSCs in epidermal regeneration.

b. Endothelial cells in skin CSC niche

Neoangiogenesis in ECs is a critical driver of CSC development and tumor progression in melanoma, SCC, and BCC, providing essential support for tumor growth and metastasis [315–317]. Several angiogenic growth factors and signaling pathways, including the VEGF family (VEGF-A, VEGF-B, VEGF-C, VEGF-D, and PLGF [Placental growth factor]), are increased in melanoma. The VEGF and its receptor can activate critical intracellular pathways, such as FAK, PI3K/ERK, PKC, and MAPK/ERK, which facilitate melanoma cell movement and tumor progression [318]. Highly proliferative MSLCs, particularly at the tumor margins, produce crucial factors for angiogenesis and are closely associated with the early onset of vascularization [319]. Particularly, MSLCs expressing ABCB5, CD133, and ABCG2 secrete proangiogenic factors, including TIE2 (Tyrosine kinase with immunoglobulin and epidermal growth factor homology domains 2), VEGF and its receptor VEGFR-2, MMP-2/−9, and angiopoietin [320–323]. Integrin α5β1 activation in melanoma cells highly expressing CD271 triggers the MAPK/ERK pathway, which activates STAT and HIF1α pathways. These signaling pathways reinforce the proangiogenic secretome of MSLCs by enhancing the production of angiogenic VEGF and IGF2 activators, while attenuating the levels of angiogenic inhibitors such as TIMP1 (tissue inhibitor of metalloproteinases 1) [324]. Inhibition of integrin α5β1 or ERK1/2 signaling reduces both MSLC characteristics and its ability to promote angiogenesis [324]. Overexpression of ALDH1A1 in melanoma cells stimulates the secretion of proangiogenic factors, including IL-8, activating the DLL4-dependent Notch signaling pathway in ECs and promoting angiogenesis. Moreover, there is a noticeable increase in the recruitment of ECs to melanomaspheres [325].

Vasculogenic mimicry (VM) refers to tumor cells adopting endothelial-like traits, reflecting the aggressiveness and plasticity of melanoma [326, 327]. The CD133^+^ and ABCB5^+^ MSLCs in perivascular niches, expressing VE-cadherin, TIE2, VEGF, and VEGFR, possess the ability to acquire the VM phenotype and contribute to melanoma drug resistance [322, 327]. Under hypoxia, endothelin-1 synthesis is induced, sustaining HIF-1α/2α-driven VEGF-A and VEGF-C expression in tumor cells and ECs, thereby promoting angiogenesis and melanoma cell migration [328]. The vasculogenic capacity and acquisition of MSLC traits are influenced by EC-derived signals, which are modulated through the endothelin B receptor (ETBR) and VEGFR [328].

MSLCs facilitate melanoma metastasis through their interaction with ECs. MSLCs engage their α4β1 integrin and E-selectin ligands with VCAM-1 and E-selectin on ECs, thereby enhancing migration along the endothelial surface [329] (Fig. 1). MSLCs can traverse EC junctions by binding their upregulated α5β1 and α6β4 integrins to fibronectin and laminin, respectively, which are present in endothelial and basement membranes [329]. Given that the attachment of MSLCs to ECs initiates transendothelial navigation, targeting the adhesion proteins involved offers a promising therapeutic strategy for impeding cancer cell extravasation.

ECs are integral in modulating the TME and constructing a vascular niche for CSCs in SCCs. As tumors advance, angiogenesis becomes increasingly pronounced, facilitating the progression of malignancy [316, 330]. Angiogenesis progressively intensifies as SCC progresses to a malignant and invasive state. The enhanced density of the microvascular network in the TME strongly correlates with the upregulation of VEGF and its VEGFR2 receptors [331]. Conversely, SCC regression correlates with a reduction in EC proliferation and vasculature density [330]. Moreover, the VEGF/VEGFR2 signaling is instrumental in advancing SCC progression by establishing a supportive vascular niche for CSCs [330]. The overexpression of VEGF in the epidermis promotes the expansion of the CD34^+^ CSC pool in SCC. These cells exhibit significantly higher VEGF expression compared to CD34^−^ tumor cells or normal keratinocytes [330]. In contrast, deficiency of VEGF or VEGFR2 in the epidermis impairs CSC proliferation and results in a remarkable reduction in SCC establishment. Neuropilin-1 (NRP1), a co-receptor of VEGF found in melanoma and SCC tumor cells, mediates the role of VEGF in maintaining CSC self-renewal. VEGF signaling activates NRP1 in CSCs, which enhances stemness and proliferative gene expression, sustaining tumor growth and CSC maintenance [330]. The deletion of NRP1 and/or NRP2 specifically in ECs profoundly hampers melanoma progression and disrupts the angiogenic processes crucial for tumor vascularization [332].

EC activity in the skin CSC niche evidently contributes to the dynamic aggressiveness of CSCs. Elucidating the mechanisms of their interactions could provide crucial insights to develop targeted therapies against malignant skin cancers.

## Signaling regulation of SSCs and CSCs

### Signaling regulation of skin stem cell niche

The balance between SSC maintenance and lineage specification is mainly regulated by four core pathways, including the Shh, Wnt/β-catenin, YAP/TAZ, and Notch signaling pathways (Fig. 2).

#### Sonic Hedgehog signaling pathway

Shh signaling is a crucial factor in the regeneration and preservation of IFESCs and HFSCs [33, 333]. Shh pathway effectors, including GLI2 and GLI3, are expressed in the outer root sheath during the active stage and in the hair germ and bulge during the resting stage of the hair cycle [33]. Hair growth regulation is influenced by GLI1 expression in the epidermis layer and dermal papillae [33]. Notably, GLI1 and GLI2 drive the activation of the Shh pathway, whereas GLI3 functions as a repressive modulator, attenuating its signaling [334, 335]. GLI1 and SOX2, key target genes of Shh signaling, serve as distinct markers for the stem cell-like subpopulation in the dermal papilla, essential for its maintenance and HF regeneration [33, 336]. Shh signaling inhibition disrupts HF cycle initiation and slows HFSC proliferation, preventing de novo HF formation [333]. The enhanced Shh levels promote the renewal of SSCs by directly activating cell cycle regulators such as cyclin E, cyclin D, N-MYC, and c-MYC [335, 337].

The Shh signaling pathway holds a crucial function in HFSC regeneration and IFESC proliferation. Patched 1 (*PTCH1)*, which encodes the PTCH1 receptor, is the main Shh transcriptional suppressor gene in the epidermis. The attenuated Shh transcriptional repressors, *PTCH1* and *PTCH2*, enhance SSC proliferation while suppressing cell differentiation [338]. *PTCH* forms a crucial negative feedback loop that dampens Shh pathway activity by sequestering the Shh ligand [339]. When Shh ligands are absent, PTCH1 receptor inhibits Smoothened (SMO) activity through a ligand-independent mechanism [339]. When Shh binds to PTCH1, it triggers degradation of the SMO-PTCH complex, which enables the phosphorylation and activation of SMO. This activated SMO then initiates the canonical Shh signaling pathway, leading to GLI-dependent transcription of crucial targets for cell proliferation, such as cyclin D, *PTCH1*, *SOX2*, *GLI1*, and *GLI2* [33, 336, 337]. In developing HFs, the expression of PTCH1 and GLI1 is highest at the proximal base and progressively decreases toward the IFE [340]. During HF maturation, the hair germ at the HF base continues to express high levels of PTCH1 transcripts in response to increased Shh ligand production [340]. Shh signaling weakens as the HF elongates and moves farther from the Shh source. In the quiescent basal IFE, low GLI1 transcriptional activity is observed in both basal IFESCs and those above the future HF bulge [340]. Therefore, a gradient of Shh levels is mainly established by *PTCH1* across different SSC regions, with hair germ HFSCs exhibiting high Shh activity, while basal IFESCs exhibit low Shh expression [340]. This is important for ensuring normal HF growth and IFESC maintenance.

The Shh pathway interacts with Wnt/β-catenin and Notch in HF formation. In the epidermis, enhanced β-catenin activity elevates Shh and its receptor Patched (PTCH) levels [341]. HES1 (Hairy and enhancer of split-1) stabilizes the GLI1^+^ population in the lower bulge or hair germ, independent of canonical Notch signaling, promoting anagen initiation [342]. Simultaneously, GLI1 activity promotes the inactivation of glycogen synthase kinase 3 beta (GSK3β) in anagen HF, resulting in increased nuclear β-catenin activation and stimulating SSC proliferation during the anagen phase of HF growth [335]. However, Shh signalling can activate Wnt5a in human dermal papillae, which reduces LEF-1 levels and subsequently suppresses Wnt/β-catenin signaling [343]. Therefore, the Shh/Wnt5a signaling promotes mature, differentiated HF formation by inhibiting proliferation and promoting differentiation of cells in human dermal papillae [344]. Collectively, the interplay of these pathways precisely controls SSC proliferation and orchestrates the dynamics of hair growth.

#### WNT/β-catenin signaling pathway

The Wnt/β-catenin pathway is integral to maintaining, regenerating, and determining the fate of SSCs during skin development. Wnt ligands bind to frizzled (FZD) and low-density lipoprotein receptor-related protein (LRP) receptors, initiating signaling that disrupts the adenomatous polyposis coli (APC)/AXIN/Glycogen synthase kinase (GSK-3β) complex, preventing β-catenin degradation [345]. Activated Wnt signaling upregulates β-catenin and LEF1, initiating a new HF growth cycle [333]. Conversely, Wnt/β-catenin deficiency accelerates the premature differentiation of IFESCs and promotes HFSC quiescence in the bulge and hair germ during the telogen phase [346]. Moreover, the Wnt3a subtype enhances SSC stemness by upregulating key factors in the BM niche, including *COL4A1*, *ITGB4*, *ITGB1* and *ITGA6* [347].

IFESCs and HFSCs rely on Wnt/β-catenin signaling to sustain quiescent SSCs and facilitate hair growth. SSCs that express *AXIN2* (Axin homolog 2), a Wnt target gene, are predominant in the basal epidermal layer and bulge niche [346, 348]. These cells can secrete Wnt gene products, including *Wnt1*, *Wnt3*, *Wnt4*, *Wnt6*, *Wnt10a*, *Wnt10b,* and *Wnt7b*, to sustain their proliferative cycle renewal and release Wnt inhibitors to modulate IFESC proliferation [346, 348]. Wnt ligands are predominantly localized in the basal layers, while elevated concentrations of Wnt inhibitors, such as DKK, are found in the suprabasal layers adjacent to AXIN2^+^ SSCs [346]. This distribution pattern may inhibit Wnt signaling in IFESCs, prompting these SCs to depart from the basal layer and trigger differentiation. AXIN2 is expressed exclusively in the outer bulge during the growth phase of the hair cycle, while the Wnt-inactive inner bulge, containing differentiated cells, expresses Dickkopf 3 (DKK3) [348]. Moreover, Wnt suppressors such as SFRP1 (Secreted frizzled related protein 1) and DKK3 secreted by AXIN2^+^ HFSCs are predominantly found in the outer regions of the bulge niche during the resting stage [346, 348]. This finding provides insight into how Wnt/β-catenin activation is suppressed during the transition of HFs into the resting phase. Consequently, AXIN2^+^ IFESCs and HFSCs can maintain their stemness while still being capable of committing to differentiation in an autocrine Wnt/β-catenin signaling manner.

Wnt signaling not only regulates skin development but also governs melanocyte stem cell (MSC) development. Its activation in MSCs promotes pigmented hair formation by driving the commitment to melanocyte fate. Wnt/β-catenin signaling directs the differentiation of neural crest stem cells (NCSCs) into melanocytes during embryonic development by regulating the homeobox gene MITF [349]. Conversely, a shortage of melanocytes occurs when β-catenin is deficient in NCSCs [35].

#### YAP/TAZ signaling pathway

Mechanosensitive signaling pathways, such as YAP/TAZ, are responsible for sensing biomechanical pressures and regulating cell fate in SSCs [350]. In proliferating basal epidermal cells, YAP is mainly nuclear, while its levels decline and it relocates to the cytoplasm during the differentiation of suprabasal cells [153, 351]. Therefore, YAP facilitates the proliferation of SSCs, keeping them in an undifferentiated state, while reduced YAP activity is crucial for epidermal cell terminal differentiation [351, 352]. The fate determination of SSCs is influenced by mechanical cues, such as tensile and compressive forces within their niche. Cell proliferation is enhanced by increased matrix stiffness through EGFR signaling activation via FA feedback [353]. Meanwhile, increased contractility reduces E-cadherin expression in SSCs, which subsequently activates YAP1 and β-catenin signaling [354]. Low mechanical pressure inactivates YAP/TAZ signaling, reducing SSC stemness and activating Notch signaling [350]. Accordingly, keratinocytes on soft substrates stop proliferating and undergo differentiation [355]. Furthermore, YAP/TAZ signaling is upregulated in a fibrous and rigid ECM environment but downregulated in a soft ECM environment [350]. Therefore, ECM mechanical properties regulate SSC behavior and fate through the modulation of YAP/TAZ signaling.

YAP/TAZ is crucial for preserving the quiescent SSC pool, which is contingent on adhesive proteins. In normal keratinocytes, the integrity of the hemidesmosome complex, which mainly consists of LM332 and integrin α6β4, regulates YAP signaling [352, 356]. Disruption of the LM332-α6β4 connection caused by harmful mutations in *LAMB3* elevates cytoplasmic YAP level [352]. The expansion of SSCs dependent on YAP/TAZ is mediated by the integrin-SRC interaction, which phosphorylates YAP directly or indirectly through PI3K stimulation, suppressing the MST-LATS in the Hippo kinase cascade [356]. This activation of YAP/TAZ subsequently suppresses Notch signaling during SSC differentiation [40]. LM332 regulates the balance between differentiation and proliferation of IFESCs by modulating YAP activity. Increased YAP/TAZ signaling and suppression of Notch activity, both associated with LM332 depletion, promote the regeneration of IFESCs [40]. In *LAMB3*-deficient junctional epidermolysis bullosa, enhanced YAP expression effectively reinstates the clonogenic potential and proliferative capacity of IFESCs [352].

Skin cell density also regulates YAP signaling via α-catenin in AJs. α-catenin prevents YAP activation by promoting YAP/TAZ-14–3-3 interaction [153]. The α-catenin-mediated sequestration of YAP1 in the cytoplasm by 14–3-3 proteins under high cell density conditions facilitates YAP1 phosphorylation [153]. This process prevents nuclear localization and enhances the proteasomal degradation of YAP, thereby ensuring proper regulation. Nuclear YAP activity increases with the loss of 14–3-3σ function, inhibiting differentiation and stimulating basal epidermal progenitor expansion [357].

The YAP/TAZ pathway interacts with other core signaling pathways to sustain the SSC niche. Notch signaling regulates YAP/TAZ activity, which, in turn, also influences Notch signaling. Abnormal hyperactivity of YAP/TAZ can hinder SC differentiation by inhibiting Notch signaling, specifically through enhancing cis-inhibition by delta-like ligand 1 (DLL1) in SSCs [350]. Conversely, weak mechanical forces generally suppress the transcriptional activity of YAP/TAZ by activating Notch signaling. This leads to the terminal differentiation of SSCs and loss of their stemness properties [350]. Overexpression of GLI2/Shh, which is linked to YAP/TAZ transcriptional activity, is critical for SSC development [358]. Additionally, YAP activation promotes SSC proliferation via the classical Wnt/β-catenin pathway by upregulating the downstream effector WNT16 [359]. YAP-driven β-catenin expression enhances mitotic activity in basal epidermal cells [360].

In summary, YAP/TAZ signaling in the SSC niche acts as a critical intrinsic regulator of skin homeostasis, primarily by driving cell division and preserving the stemness of SSCs.

#### Notch signaling pathway

Notch signaling regulates SSCs by controlling their epidermal differentiation, proliferation, and fate determination. Activated Notch signaling coincides with increased differentiation markers, including spinous-specific genes like keratin 1/10, involucrin and the negative cell cycle regulator p21 [361], which represses Wnt4 expression [362]. Consequently, keratinocytes commit to leaving the SSC compartment, leading to their detachment from the BM and commence differentiation.

The differentiation of SSCs and MSCs is driven by Notch signaling, mediated by Notch1–4 receptors and ligands such as DLL1 and JAGGED1/2 within the SSC niche [363, 364]. Notch1, the most active receptor in the epidermis, is expressed across both basal and suprabasal layers, and is found in SSCs, TACs, and differentiated keratinocytes [363, 364]. In contrast, Notch2 and Notch3 are mainly expressed in the suprabasal layers, particularly in the spinous and granular cells, with Notch4 being nearly absent [363–365]. While inactivation of Notch2 and Notch3 impairs terminal differentiation, knockdown of Notch1 predominantly accelerates cell proliferation, with a negligible impact on differentiation [363]. JAGGED1/2 and DLL1 are predominantly expressed in the basal layer, where JAGGED1/2 promotes terminal differentiation, while DLL1 counteracts this effect [363, 364]. Ligation of Notch ligands (DLL1 or JAGGED1/2) to Notch1 receptors in sender cells induces a cis-inhibition process. This enhances the binding of Notch receptors in neighboring receiver cells to ligands in sender cells, a phenomenon known as transactivation [363]. Consequently, this activates Notch signaling in adjacent receiver cells by promoting paracrine Notch-ligand interactions. Activation of the Notch receptor leads to cleavage of its intracellular domain, which then translocates to the nucleus. In the nucleus, it associates with RBPJ (transcription factor C promoter-binding factor 1/recombination signal binding protein J/κ), a transcription factor, to regulate the expression of genes such as *HEY1* (hairy/enhancer-of-split related with YRPW motif 1) and *HES1* [365].

Second, Notch signaling induces SSC differentiation by influencing the cohesion and formation of boundaries within the epidermis. Notch signaling induces basal keratinocyte detachment from the BM by downregulating α3β1 and α6β4 integrins and upregulating differentiation markers such as involucrin and keratin 1 [361]. Additionally, activated Notch in human epidermal cells and SCC tumor cells inhibits the expression of ROCK2 and MRCKα (myotonic dystrophy kinase-related Cdc42-binding kinase), impairing cell migration and favoring the development of well-differentiated tumors by disrupting the expression of integrin α6β4 and Wnt7a [366]. In contrast, increased activity of these molecules suppresses cell differentiation and enlarges the stem cell pool in SCC [366]. Therefore, Notch1 functions as a tumor-suppressive regulator within the homeostatic SSC niche by preventing hyperproliferation.

Thirdly, Notch signaling facilitates differentiation by downregulating p63 through a non-canonical pathway. p63 is prominently expressed in proliferative SSCs, with a marked reduction as cells undergo differentiation into suprabasal layers [367]. Notch activation suppresses p63 expression by selectively influencing the interferon regulatory factors (IRFs), leading to decreased levels of IRF3 and IRF7 [367], while increasing IRF6 [368]. However, the reduction in p63 and its downstream effectors creates a negative feedback loop, which prevents excessive differentiation and maintains the SSC pool by inhibiting the Notch target gene *HES1* through p63 [367]. Therefore, the interplay between Notch signaling and p63 is crucial for regulating SSC development.

Fourthly, Notch signaling governs MSC development and differentiation, as evidenced by the elevated expression of Notch1 and its transcription factor HES1 in MSCs of the bulge niche [369]. Deletion of Notch1 and Notch2 increases *HES1*-mediated apoptosis of MSCs and hair pigmentation disorders [369]. Maintenance of the immature state and migration of melanoblasts are primarily regulated by Notch signaling. Notch inactivation leads to a graying hair phenotype, which can be reversed by overexpression of HES1 in the melanocyte lineage [369].

Finally, in addition to Notch-mediated SSC differentiation, intrinsic signaling helps sustain the SSC population. Notch ligand-mediated cis-inhibition of Notch in SSCs, driven by YAP/TAZ or Fringe enzymes, preserves the undifferentiated state of SSCs [363]. Fringe proteins, known for binding to the EGF repeats of the Notch extracellular domain (NECD), can modify the affinity of the NECD for ligands in neighboring or the same cells [370]. The most prominent of these is the lunatic fringe (LFNG), which is highly expressed in basal epidermal cells rich in DLL1 [364]. LFNG enhances the affinity of Notch for DLL1 while simultaneously inhibiting signals from JAGGED [371]. In cells overexpressing LFNG, reduced differentiation is reflected by decreased Notch downstream differentiation markers and HES1, while increased proliferation is indicated by elevated p63 levels [364]. In contrast, JAGGED ligands induce differentiation in keratinocytes, selectively downregulating LFNG expression [364]. Elevated levels of DLL1 enhance keratinocyte adhesion independently of Notch signaling, while impairing Notch activity, which inhibits the terminal differentiation of epidermal cells [371]. Thus, DLL1, despite being present at lower concentrations than other Notch ligands and localized in SSCs, significantly contributes to sustaining stemness, with LFNG expression amplifying its function [364].

In summary, Notch signaling has significant effects on SSC differentiation, with its ability to either promote or inhibit differentiation determined by its engaging ligands. When this pathway is disrupted, Notch can adopt opposing roles, either promoting tumor suppression or driving oncogenesis.

#### PI3K/AKT/mTOR and JAK/STAT3 signaling pathway

The PI3K/AKT/mTOR and JAK/STAT3 signaling pathways play key roles in survival, proliferation, and regeneration of SSCs. In the SSC niche, PI3K/AKT/mTOR facilitates epidermal differentiation, while JAK/STAT3 helps maintain the resting state of SSCs. The positive impact of these pathways on cell proliferation and HF growth is particularly noticeable during epidermal regeneration following injury, inflammation, and cancer development [372–376].

The survival and proper differentiation of cells depend on PI3K/AKT signaling [372]. During SSC differentiation, this pathway can be activated by EGFR, SRC family tyrosine kinases, and E-cadherin-mediated adhesion [114, 356, 377]. Extracellular ligands bind to tyrosine kinase receptors, activating PI3K phosphorylation. This leads to the conversion of phosphatidylinositol (4,5)-bisphosphate (PIP2) to phosphatidylinositol (3,4,5)-trisphosphate (PIP3), which recruits AKT to the plasma membrane and activates mTORC1. This cascade then activates downstream effectors essential for survival, cell proliferation, and metabolism [378, 379]. Active AKT is mainly found in post-mitotic suprabasal keratinocytes and in a subset of basal SSCs, which are loosely anchored to the BM and begin to migrate toward the spinous layer [372]. AKT activation causes growth arrest in keratinocytes and initiates their differentiation. In contrast, the majority of proliferating basal SSCs do not exhibit AKT activation [372]. In addition, PI3K signaling is crucial for preventing premature cell death in differentiating keratinocytes. Inhibition of this pathway activates selective caspase-3 and apoptosis in differentiating keratinocytes, while proliferating keratinocytes remain unaffected [372]. Consequently, the PI3K signaling pathway governs epidermal cell differentiation and prevents apoptosis-driven cell death during this process.

JAK/STAT3 signaling helps prevent SSC exhaustion and depletion by inhibiting their proliferative and activating capacity. JAK/STAT3 is implicated in anti-hair growth signaling [380]. Inhibition of this pathway prompts HFSCs to exit their resting state and re-enter the hair cycle [380]. In aged skin, inhibition of JAK/STAT3 signaling increases SSC proliferation in the IFE and bulge niche [381]. On the other hand, hyperactive JAK/STAT3 signaling in aged mice is closely tied to impaired HFSC function [381].

STAT3 activation, a key component of JAK/STAT signaling, helps prevent programmed cell death and promotes proliferation of SSCs during skin regeneration, particularly in wound healing and HF growth [375, 376]. The absence of STAT3 profoundly impairs the HF cycle activation, affecting the healing process [375]. Cytokine receptors (e.g., IL-6, IL-11, CCL2), receptor tyrosine kinases (e.g., EGFR), and non-receptor tyrosine kinases like SRC can activate STAT3 [382, 383]. Phosphorylated STAT3 dimerizes and moves to the nucleus, and modulates its targets such as cyclin D1, Bcl-xL (B-cell lymphoma-extra large), c-MYC, and TWIST [384].

STAT3 promotes epidermal cell differentiation and HF maturation. Its activation is evident in differentiating keratinocytes, whereas the abrogation of STAT3 activation results in a concomitant diminution in the expression of the differentiation marker keratin 1 [385]. Moreover, keratinocytes lacking STAT3 exhibit increased adhesiveness and impaired migratory capacity [386]. Conversely, the constitutively active STAT3C variant of STAT3, facilitates HFSC maturation within the bulge region and promotes their egress from the niche [387].

### Signaling regulation of skin cancer stem cell niche

Signaling regulation in the normal SSC niche operates as a tightly woven network, where each signaling pathway and niche factor is precisely positioned to interact with surrounding elements, ensuring balanced epidermal homeostasis. Dysregulation of these fundamental signaling pathways, whether through pro-oncogenic events, latent mutations, or SC niche disturbance, can trigger uncontrolled proliferation and differentiation, thereby initiating and sustaining CSC populations. This complexity presents a significant challenge for effective skin cancer treatment. In the following sections, we will focus on how core signaling pathways in the SSC niche contribute to the CSC niche (Fig. 3), with particular emphasis on melanoma, SCC, and BCC.

#### Sonic Hedgehog signaling pathway

Shh signaling is crucial for preserving the SSC pool, and its overactivation drives the continuous growth and expansion of CSCs. Shh signaling is a critical pathway involved in maintaining CSC properties in melanoma. MSLCs express pluripotency factors (*OCT4, SOX2*, and *NANOG*) and genes related to Shh downstream signaling (*SHH*, *GLI2*, *GLI3, SMO*, and *PTCH1*) [388]. Blocking both *SMO* and *GLI1* significantly inhibits ALDH^+^ MSLC self-renewal and tumorigenesis [388]. The oncogenic gene *WIP1* positively regulates Shh activity in melanoma, and its silencing limits melanosphere formation by impairing *GLI1* and Shh signaling activation [389].

Abnormal Shh signaling is a prominent mechanism in BCC. PTCH1-mediated Shh repression is relieved through enhanced Shh binding to PTCH1 or mutational inactivation of *PTCH1*, resulting in unregulated SMO activation [390]. Hyperactivation of the Shh pathway enhances the CSC population in the epidermis, driving epidermal hyperproliferation and facilitating oncogenic progression of BCC [384]. GLI1 and GLI2 expression are tightly associated with the HFSC markers LGR4 and LGR5 in BCC [391]. Furthermore, cells expressing GLI1 are predominantly localized in the hair bulb, a critical reservoir of CSCs involved in BCC formation, with their localization largely overlapping the region expressing LGR5 [391, 392]. In addition, GLI1 is significantly upregulated and is necessary for the CD200^+^ cells in BCC tumors to function properly [20]. CD200^+^CD45^−^ cells in BCC display stem cell-like properties and chemotherapy resistance, with sustained Shh activation [20]. These findings propose a potential BCC treatment strategy combining anti-CD200 neutralizing antibodies and SMO inhibitors.

#### Wnt/β-catenin signaling pathway

In the CSC niche, dysregulation of the Wnt/β-catenin pathway disrupts its ability to prevent CSC hyperproliferation. Silencing β-catenin depletes CD34^+ ^CSCs and suppresses SCCs, whereas overexpression of nuclear β-catenin results in SCC-like tumors and an expanded CSC pool in the bulge [393]. Furthermore, diminished Wnt/β-catenin correlates with reduced levels of its effector SOX9 and impaired tumor cell proliferation in metastatic SCCs [394].

Wnt/β-catenin signaling is crucial for melanoma initiation and metastasis, regulating the functional and biological traits of MSLCs. Its activation promotes EMT and fosters the acquisition of stem-like properties in melanoma [395]. For instance, it enhances the prometastatic properties of MSLCs that express CD133 [396]. Disruption of Wnt/β-catenin signaling in MSLCs diminishes stemness features, impairs migration, and limits proangiogenesis [397]. Targeting the Wnt/β-catenin pathway to eliminate CSCs could significantly enhance the effectiveness of melanoma treatments.

EMT enhancement and accelerated skin cancer metastasis are driven by the Wnt/β-catenin signaling cascade. A critical event in melanoma, EMT initiation, involves the disruption of β-catenin/E-cadherin complexes. This process liberates β-catenin, which in turn hyperactivates the Wnt pathway, driving the progression and metastatic potential of melanoma [398]. Additionally, Wnt5a overexpression in melanoma cells activates PKC, upregulating MMP-2 and promoting cell spread [399]. This process decreases E-cadherin expression and simultaneously upregulates EMT markers such as vimentin and SNAIL [399]. SNAIL and SLUG promote β-catenin/LEF1 transcription during EMT by downregulating E-cadherin, which allows β-catenin to translocate to the nucleus [400].

#### YAP/TAZ signaling pathway

YAP/TAZ hyperactivation enhances the growth and survival of melanoma, SCC, and BCC tumors in a fibrotic and dysregulated signaling niche. High YAP/TAZ activity contributes to BCC and SCC development. The knockout of YAP/TAZ in epidermal basal cells inhibits BCC and SCC tumor formation [28]. Enhanced expression of YAP/TAZ has been implicated in melanoma progression and may significantly affect postoperative survival outcomes in patients [401].

Condensed ECM activates YAP/TAZ signaling, maintaining stemness in tumor cells within the CSC niche. The stiff ECM surrounding the SCC tumor stimulates YAP/TAZ signaling through Hippo-independent pathways, including PI3K/AKT and ERK [402]. Ectopic expression of YAP in BCC is influenced and promoted by the stiffness of the tumor niche. Shh/GLI2 signaling, which mediates the c-JUN/AP1 (Activator protein 1) axis, may promote YAP function [358, 403]. Furthermore, hyperactivation of YAP and its downstream molecules, such as connective tissue growth factor (CTGF) and cysteine-rich protein 61 (Cyr61), was observed in BCC [404]. Notably, Cyr61 can induce aberrant proliferation of epidermal basal cells and CTGF can remodel the tumor microenvironment [404].

YAP/TAZ plays a key role in determining MSLC fate within various ECM environments in the CSC niche. In a collagen-induced stiff matrix, nuclear YAP/TAZ signaling is triggered by enhanced FAK/SRC phosphorylation, regulated by FAs [405]. This activation promotes a differentiated/proliferative MITF-high phenotype in melanoma cells, which exhibit enhanced spreading in a rigid collagen matrix [405]. This contrasts with the canonical YAP/TAZ pathway via YAP/TEAD signaling, which promotes the resting state of SSCs and CSCs. YAP/TAZ-induced differentiation of melanoma cells occurs through a transcriptional interaction with PAX3 (Paired box 3), which activates MITF gene expression in these cells [405, 406]. Nevertheless, YAP/TAZ suppresses MITF expression and induces a de-differentiated melanoma phenotype in a fibroblast-rich matrix. This effect is attributed to fibroblast-secreted TGF-β, which activates YAP/TEAD transcription while inhibiting YAP/PAX3-driven MITF expression [405]. Mechanistically, TGF-β downregulates PAX3 expression [407], limiting the availability of PAX3 for YAP binding. Moreover, PAX3 depletion decreases YAP association with the MITF promoter and increases its interaction with the CTGF (connective-tissue growth factor) promoter [405], which is responsive to TGF-β [408]. In summary, YAP/TAZ promotes melanoma cell proliferation and invasion while preserving MSLC stemness. Therefore, combining treatments that target stromal components to modulate various aspects of YAP/TAZ activity may help overcome resistance in melanoma and other skin cancers.

#### Notch signaling pathway

In skin cancers, Notch signaling plays dual roles as both an oncogene and tumor suppressor, contrasting its tumor-suppressive function in the SSC niche. Loss-of-function mutations that inactivate Notch contribute to cutaneous SCC formation by disrupting its role in differentiation and preventing hyperproliferation [409]. Alterations in *NOTCH1* and *NOTCH2* genes, leading to their inactivation, are frequently observed in both SCC and BCC. Among 88 SCC tumors, *NOTCH1* mutations were found in 55.4% of cases, and *NOTCH2* mutations in 36.1% [410]. Of the 293 BCC tumors analyzed, 25% of 85 BCCs and 30% of 76 BCCs exhibit loss of function mutations in *NOTCH1* and *NOTCH2*, respectively [411]. These findings align with the role of Notch signaling in restraining cancer progression in skin malignancies [412]. These loss of function mutations impair receptor-ligand interactions, thereby disrupting signaling pathways [410, 411, 413, 414]. In SCC patients, mutations in the EGF-repeat region, such as D469G (missense) in *NOTCH1* and Q610* and W330* (nonsense) in *NOTCH1* and *NOTCH2*, respectively, impair the binding of the Notch receptor to DLL1 [415], effectively blocking DLL1-dependent signaling. Another type of mutation that impairs Notch function involves disruption of Notch/RBPJ complex formation. For example, the P1770S mutation in the RAM (RBPJk–associated molecule) domain of *NOTCH1* interferes with its binding to RBPJ, preventing the assembly of functional Notch transcription complexes [416]. This disruption blocks the activation of downstream gene expression mediated by Notch signaling. A novel mechanism driving *NOTCH* inactivation in SCC is through mutations in its transcriptional regulator, EP300 (p300) [410]. The histone acetyltransferase domain of EP300, essential for its function as a transcriptional co-activator of *NOTCH1*, harbors hotspot mutations [417]. Additionally, the expression of HES1, HEY1, and the canonical Notch transcription factor RBPJ is downregulated in CD133^+^ stem-like subsets in SCC tumors [418]. Despite the high frequency of *NOTCH* driver mutations in skin cancers, their direct relationship with CSCs is yet to be explored.

On the other hand, suppressing Notch1 diminishes tumor growth, likely due to its non-canonical functions. Inhibition of Notch1 signaling reduces the number and tumorigenicity of CD133^+^ CSCs [418]. This observation pertains specifically to the impairment of the IKKα/NF-κB axis, a non-canonical Notch pathway that induces tumor cell death. In CD133^+^ CSCs from SCCs, upregulation is seen in genes associated with the Notch (*JAGGED2*, *NOTCH1*), Shh (*GLI2*, *PTCH1*, *SUFU*), and Wnt (*CTNNB1*, *WNT3*) pathways [418]. Notch signaling promotes melanoma cell survival and invasiveness by post-translationally stimulating the PI3K/AKT pathway [189]. Conversely, stabilized HIF-1α, in conjunction with PI3K/AKT activation, enhances Notch signaling in melanoma [419]. These findings suggest that dysregulated Notch signaling, skewed toward a non-canonical pathway and hindering classical Notch function, may redirect Notch from its anti-tumor role to a tumorigenic promoter.

Notch signaling is critical for sustaining CSC populations. In melanoma, Notch activity enhances melanoblast proliferation and CD133 expression, thereby facilitating MSLC expansion [321]. Inhibition of γ-secretase and tumor necrosis factor-alpha converting enzyme (TACE) prevents melanosphere establishment by downregulating NICD2 and HES1 [420]. Additionally, Notch signaling promotes cyclin D1 expression, driving the G1 to S phase transition [421]. Mechanistically, Notch signaling activation supports the preservation of MSLCs by either inducing its downstream effector HES1 [369] or activating HIF1α/β, promoting the upregulation of tumor-related genes such as *VEGF* [422]. Deletion of Notch1 or CD133 leads to the downregulation of SNAIL and SLUG, while E-cadherin expression is upregulated [321]. Additionally, CD133 activation initiates the MAPK pathway, increasing MMP-2, MMP-9, and VEGF expression, thereby promoting angiogenesis and tumor development [321]. Furthermore, Notch1 enhances the proangiogenic properties of melanoma cells that overexpress ALDH1A1, thereby facilitating tumor vascularization and progression [325].

Notch3 and Notch4 are elevated and crucial for maintaining the plasticity of MSLCs [423, 424]. Silencing Notch3 results in a reduction of stemness markers such as CD133 and CD271, depletes the melanoma CSC population, and weakens their proangiogenic activity [424]. Inhibition of VM and tumor blood vessel formation due to Notch3 inactivation destabilizes MSLC homeostasis [424]. Notch4 drives the aggressive behavior of melanoma, promoting processes such as VM and growth independently of attachment [425]. Targeting Notch3 or Notch4 could disrupt the vascular niche and suppress stemness markers, thereby eliminating established MSLCs and preventing the formation of new MSLC subsets [423, 424].

#### PI3K/AKT/mTOR and JAK/STAT3 signaling pathway

The PI3K/AKT/mTOR and JAK/STAT3 signaling cascades are commonly aberrant in skin malignancies, facilitating tumor persistence by exerting anti-apoptotic effects and driving proliferative expansion [374, 379, 384, 426–429].

The PI3K/AKT/mTOR axis is crucial for SCC progression. Aberrant ECM components, such as LM332, in the SCC microenvironment trigger the PI3K/AKT pathway. The β3 domain of LM332 (V-III), which is highly enriched during SCC invasion, interacts with collagen VII rather than with integrins, activating the PI3K pathway and driving SCC progression [430]. Moreover, oncogenic RAS initiates SSC tumorigenesis through the activation of the PI3K/AKT/mTOR pathway as a downstream effector [431].

Maintaining the stemness of MSLCs and enhancing their resistance to drugs are both regulated by the PI3K/AKT/mTOR pathway. PI3K activation subsequently activates downstream pathways, such as PI3K/MDM2 (Mouse double minute 2) and PI3K/AKT/MKP-1 (Mitogen-activated protein kinase phosphatase 1), which drive self-renewal, sustain stemness, and contribute to treatment resistance in CD133^+^ MSLCs [432]. The PI3K/AKT/MDM2 pathway destabilizes p53, whereas the PI3K/AKT/MKP-1 pathway suppresses JNK and p38-MAPK activity [432]. Both PI3K/AKT/MKP-1 and PI3K/AKT/MDM2 pathways are instrumental in blocking apoptosis. Additionally, MSLCs rely on the PI3K/AKT pathway for their VM formation and angiogenesis, processes essential for tumor spread and metastasis [433]. Blocking PI3K activity prevents MSLCs from forming VM structures [433].

JAK/STAT3 signaling plays a pivotal role in the oncogenesis, progression, and maintenance of CSCs in skin cancers, with its activation being crucial for the formation of SCC [428, 434], BCC [384], and melanoma [429]. STAT3 activation facilitates the growth and persistence of tumor cells during SCC carcinogenesis [435]. Conversely, STAT3 deletion in the basal epidermal layer prevents epidermal hyperproliferation during SCC progression [428]. Mechanistically, STAT3 deletion lowers the levels of cell cycle mediators (c-MYC, cyclin D1, and cyclin E) and survival proteins (Bcl-xL) in tumor cells [120, 435]. STAT3 regulates HFSC behavior in the bulge niche during early skin tumorigenesis. STAT3 ablation at this stage significantly reduces tumor establishment and increases HFSC apoptosis [436]. Furthermore, in the absence of STAT3, CSC proliferation in response to Shh signaling is significantly diminished, impairing the initiation of BCC tumorigenesis [384]. STAT3 activation drives the upregulation of cyclin D1, which accelerates cellular proliferation, thereby facilitating CSC accumulation and BCC development [384].

Hyperactivation of STAT3 is crucial for the invasion and metastasis of skin cancers, including SCC [437–440], BCC [440, 441] and melanoma [442, 443]. In melanoma, poor prognosis and unfavorable clinicopathological features are associated with STAT3 hyperactivation [443]. STAT3 suppression triggers apoptosis, enhancing melanoma cell death [429]. Disruption of the STAT3 pathway impedes tumor progression and suppresses the secretion of soluble factors by SCC tumor cells [439, 444, 445]. STAT3 further potentiates the metastatic capability of SCCs by downregulating E-cadherin and activating TWIST, which drive EMT [437, 444, 446]. Consequently, STAT3 is integral not only to the initiation of skin carcinogenesis but also to its metastatic advancement. 

JAK/STAT3 maintains survival and reinforces the CSC phenotype in the skin CSC niche. In SCC tumors, SNAIL overexpression preserves the undifferentiated state of CSCs during metastasis [447], which is associated with the increased activation of STAT3 [448]. Inhibition of STAT3 phosphorylation disrupts self-renewal in SNAIL-expressing CSCs and downregulates stem cell markers, such as OCT4, SOX2, NANOG, and CD44 [447]. Consistently, the absence of STAT3 in SNAIL-driven SCC tumors results in diminished tumor size and growth. Notably, SNAIL-expressing CSCs can sustain STAT3 activation in an autocrine manner. This is mediated by the secretion of Mindin (Spondin-2), a protein in the SNAIL-expressing epidermal cell secretome [449] that is required for SSC stemness preservation and engages SRC to activate the STAT3 signaling cascade [447]. Furthermore, STAT3 is essential for sustaining the Shh-driven CSC population during the formation and progression of BCC [384]. These findings emphasize the need for deeper exploration of the ECM in the CSC niche and the secreted factors from CSCs, which engage with signaling pathways to sustain skin cancer progression.

### Crosstalk between signaling pathways in the SSC and CSC niche

Signaling regulation within the normal SSC niche operates as an intricately connected network, where each signaling pathway and niche factor is strategically placed to interact with surrounding elements, ensuring the maintenance of balanced epidermal and HF development. Pro-oncogenic events and latent malignant risks, such as mutations, can induce dysregulated proliferation and differentiation by disrupting the critical regulation of fundamental signaling pathways. For instance, disruption of regulatory control over cell growth signals from the Wnt and Shh pathways in the SC compartment occurs through loss-of-function mutations in *NOTCH1* or Shh inhibitor *PTCH* [390, 412]. Furthermore, the Shh and Wnt signaling pathways mutually enhance each other in skin cancers, amplifying tumor cell proliferation [450, 451]. In the CSC niche, reduced DKK levels [452] and increased nuclear β-catenin [359] contribute to the hyperactivation of Wnt signaling. Additionally, enhanced metastatic and proliferative capabilities in tumor cells are promoted by the overexpression of Notch and its ligands [189, 453]. YAP/ TAZ hyperactivation in the CSC niche, crucial for maintaining CSC stemness, is driven by a stiff ECM and disrupted cell adhesion [126, 153]. These dysregulated signaling pathways and their crosstalk facilitate the accumulation of oncogenic mutations and help sustain CSC populations.

The interplay between Shh and Wnt signaling may complicate melanoma treatment. Loss of *PTCH* function releases SMO inhibition, leading to excessive Shh activity and enhanced expression of downstream Wnt proteins [454]. Wnt5a, a main target of the Shh pathway, can activate both canonical and non-canonical Wnt signaling [455]. In melanoma, Wnt5a promotes tumor cell proliferation by activating ADP-ribosylation factor 6 (ARF6) through interaction with FZD4 or LRP6, which in turn facilitates β-catenin nuclear translocation [456]. Additionally, Wnt5a drives tumor cell invasion via the non-canonical Wnt pathway, engaging PKC [399], CDC42 [457] or JNK/c-JUN [458, 459] signaling cascades. Wnt5a activation induces an invasive phenotype in melanoma by promoting SNAIL-mediated downregulation of E-cadherin, thus facilitating EMT during tumor progression [399]. Moreover, canonical Wnt signaling can interact with Shh through its downstream genes, *LGR4* and *LGR5*, in BCC. The ablation of *PTCH1* in LGR5^+^ SSCs promotes the formation of BCC-like tumors [460]. Overexpression of SSC markers *LGR4* and *LGR5*, which enhance *GLI1/2* gene expression, was observed in BCCs [391]. GLI proteins induce the expression of *SNAIL*, promoting nuclear β-catenin accumulation and facilitating EMT in BCCs [461].

The interaction between Shh and YAP/TAZ or Notch signaling is critical for BCC initiation and progression. Generally, Shh drives oncogenesis in BCC, whereas Notch acts to prevent tumor formation. Notch1 loss in the skin sustains GLI2 and β-catenin activation, fostering BCC-like tumor formation [412]. In contrast, YAP transcriptional activity exerts a synergistic effect with the Shh signaling pathway in enhancing CSC proliferation. Epidermal YAP activation stimulates β-catenin signaling in basal SSCs, facilitating substantial nuclear expression of GLI2 in BCC [358] and promoting epidermal hyperproliferation [360]. This YAP/β-catenin-induced GLI2 hyperactivation is concomitant with enhanced RHOA/ROCK signaling and elevated ECM fibrous deposition in human BCC [358].

The mechano-transduction pathway, increased by elevated protein deposition and increased stiffness of the ECM, drives the hyperactivation of multiple signaling pathways and their crosstalk, ultimately promoting the expansion of CSCs. The mechanical forces present in the CSC niche promote β-catenin nuclear accumulation and stimulate oncogenic pathways, including integrin-mediated FAK and PI3K/AKT signaling pathway [462]. Aberrant integrin-FAK-SRC signaling contributes to an aggressive SCC variant [463] and sustains the CSC population [19]. Increased ECM density can stimulate the integrin-FAK signaling pathway, resulting in PI3K/AKT activation in hyperproliferative and invasive SCC tumors. Nuclear β-catenin accumulation is enhanced by AKT-mediated GSK3β phosphorylation, which prevents its degradation [462, 463].

In the skin CSC niche, STAT3 activation is facilitated by SRC, a non-receptor tyrosine kinase. SRC kinase inhibition impedes STAT3 signaling, thereby attenuating the proliferative capacity of melanoma cells [429]. Blocking STAT3 or SRC signaling decreases key cell survival proteins, including Bcl-xL and Mcl-1 (Myeloid cell leukemia-1), precipitating apoptosis in melanoma cells [429]. These findings underscore the indispensable functions of STAT3 and SRC signaling in sustaining tumor cell viability and facilitating malignant progression in melanoma. Moreover, the STAT3 signaling pathway is pivotal in conferring stem-like properties and tumorigenic capabilities to SCC cells. Notably, this effect is mitigated in the absence of SRC within SCC tumors that express SNAIL [447]. In addition, STAT3 signaling activation is frequently observed in Shh signaling-driven BCCs. Ablation of STAT3 results in a marked reduction in both tumor volume and proliferation mediated by Shh signaling. Additionally, Shh-induced expansion of CSCs is significantly attenuated in the absence of STAT3 in BCC tumors [384].

The intricate interplay between the signaling pathways in the CSC niche warrants further exploration. A deeper understanding of these interactions could enhance therapeutic efficacy by targeting and reducing the CSC population in skin cancers.

## Drug targeting and clinical implications

A promising therapeutic strategy for addressing drug resistance and recurrence involves targeting skin CSC niche. Various strategies have been designed to target CSC niche and eliminate the CSC population in skin tumors. The core signaling pathways, namely Shh, Wnt/β-catenin, YAP/TAZ, Notch, PI3K/AKT/mTOR, and JAK/STAT3 have been reported to stimulate the expansion of skin CSCs (as shown in Fig. 3 and Table 2). The potential signaling pathways targeted in both current and ongoing clinical trials (Fig. 4 and Table 1) are summarized below.

### Targeting Shh signaling pathway

Targeting the Shh signaling pathway can be achieved through the use of inhibitors targeting Shh, GLI, and SMO (Fig. 4A). For the treatment of advanced BCC, vismodegib and sonidegib received approval from the US Food and Drug Administration (FDA) in 2012 and 2015, respectively [464]. Clinical studies have shown that vismodegib (GDC-0449, a SMO inhibitor) is effective in treating late-stage BCC [465, 466]. The objective response rates (ORRs) for metastatic BCC (mBCC) and locally advanced BCC (laBCC) patients were 30%–30.8% and 43%–46.4%, respectively [465, 467]. For mBCC and laBCC patients, overall response rates ranged from 30.3% to 50% and 42.9% to 60%, respectively, with median response durations between 7.6 and 8.8 months [466, 468]. For individuals affected by nevoid basal cell carcinoma syndrome, vismodegib reduced tumor burden and prevented the development of new BCCs [469]. Furthermore, a clinical trial showed that sonidegib treatment resulted in ORRs for 194 laBCC and 36 mBCC patients ranging from 46.1% to 56% and 8% to 17%, respectively [470]. This trial also had a long-term 42-month follow-up on safety and efficacy [470].

Despite the significant effects of vismodegib and sonidegib on shrinking tumors and delayed progression, some patients experienced BCC recurrence upon discontinuation of the drugs [469, 471, 472]. The main reasons for treatment failures may involve refractory quiescent CSCs and SMO mutations [473–476]. The ERIVANCE phase II clinical trial identified concerns regarding the safety profile of vismodegib, with disease progression cited as the reason for treatment discontinuation in 27.9% of the 104 advanced BCC patients [477]. A retrospective review found that 21% of 28 advanced BCC patients who received vismodegib treatment experienced tumor recurrence within 56 weeks [478]. Similarly, trials of sonidegib also reported continuous tumor progression in some patients [470, 479]. Notably, patients with a history of resistance to an SMO inhibitor exhibited a secondary resistance event when treated with another SMO inhibitor [480]. Additionally, a contentious association has been observed between vismodegib treatment for BCC and a high susceptibility to developing SCC [481, 482].

Other SMO antagonists have been evaluated in phase I and II trials for treating advanced or metastatic BCC and melanoma. These include IPI-926 (NCT00761696, NCT02828111, NCT01609179, and NCT02762084), itraconazole (NCT01108094 and NCT02120677) [483, 484], BMS-833923 (XL139; NCT00670189), taladegib (NCT01226485), LEQ506 (NCT01106508), vitamin D3 (calcitriol; NCT00301067 and NCT01358045), and ZSP1602 (NCT03734913). Oral IPI-926 (saridegib/patidegib) was well-tolerated and demonstrated efficacy in BCC patients, showing anti-tumor activity in those previously untreated with vismodegib [485]. Topical application of patidegib (IPI-926) has shown promising results in reducing tumor size and decreasing *GLI1* mRNA levels after 12 weeks in phase II trials (NCT02828111) [486]. The topical variant of IPI-926 has also been shown to be safe and effective, with minimal risks of systemic adverse events [486]. Itraconazole, an antifungal agent that can inhibit the Shh pathway, has demonstrated some efficacy in BCC patients (NCT01108094). However, congestive heart failure and fatigue were reported as complications in two patients [487], emphasizing the need for further research to assess the safety of this treatment over time.

In populations resistant to SMO inhibitors, alternative compounds have been investigated to target other molecules in the Shh signaling pathway. For instance, arsenic trioxide (ATO; NCT01791894), nicotinamide (NCT03769285 and ACTRN12612000625875 [488]), and casein kinase 2 inhibitor (CX-4945, silmitasertib) (NCT03897036) have been studied for their ability to target *GLI* transcription factors. Nicotinamide is thought to suppress GLI activity by increasing cytoplasmic sirtuin 1 (SIRT1) level [489]. A phase III trial involving oral administration of nicotinamide (500 mg twice per day) demonstrated a safe and effective regimen for preventing new BCC and SCC development [488]. Furthermore, topical imiquimod (Aldara), FDA-approved in 2004 for primary superficial BCC treatment, inhibits the Shh pathway by inducing protein kinase A-mediated GLI phosphorylation [490].

### Targeting Wnt/β-catenin signaling pathway

Suppressors of Wnt/β-catenin activation in skin cancers include β-catenin destruction complex enhancers and porcupine inhibitors like WNT974 (NCT01351103) (Fig. 4B). The initial results of this trial indicated reduced AXIN2 expression, a marker of Wnt/β-catenin activity, in melanoma patients [491]. With only 4% of 94 patients encountering dose-limiting toxicities during the first course of treatment, WNT974 demonstrated good tolerance [491]. In a phase I/II clinical trial, the tankyrase inhibitor E7449, which stabilizes Axin protein and suppresses β-catenin, is being assessed for its potential in treating solid tumors, including advanced melanoma (NCT01618136) [492]. Sulindac (NCT00755976) and aspirin (NCT03396952) are also being investigated as treatments for melanoma in phase II clinical trials, due to their ability to increase GSK3β phosphorylation and β-catenin degradation [493, 494].

### Targeting YAP/TAZ signaling pathway

SRC inhibitors have shown potential as drugs that inhibit YAP/TAZ activity in skin cancers (Fig. 4C). The efficacy of SRC inhibitors, such as dasatinib, nilotinib, and saracatinib, has been evaluated in clinical trials for melanoma. In trial NCT00597038, the dasatinib-dacarbazine combination yielded a 72.4% clinical benefit rate in 29 metastatic melanoma patients [495]. A phase II study (NCT00700882) found that KIT-positive melanoma had a higher partial response rate (18% or 4/22 tumors) than KIT-negative melanoma (5.9% or 3/51 tumors) [496]. Notably, KIT-mutated metastatic melanoma patients treated with nilotinib showed stable disease for more than six weeks in 47.6% of cases (*n* = 42), without noticeable side effects [497]. Overall, phase I/II trials in advanced melanoma have also observed partial and minor responses to SRC inhibitors [495–498].

Histone deacetylase (HDAC) inhibitors have been shown to effectively block nuclear YAP translocation by suppressing its gene transcription [499]. HDAC inhibitors specifically target cancer cells with hyperactive YAP expression. In phase I/II clinical trials for skin cancers, vorinostat, an HDAC inhibitor, was evaluated. Two melanoma patients in a phase I clinical trial (NCT00331955) maintained stable disease for at least 8 months following treatment with a combination of vorinostat and doxorubicin [500]. A phase II trial (NCT00121225) in advanced melanoma patients reported that the major side effects of vorinostat included fatigue, nausea, lymphopenia, anemia, and hyperglycemia.

Pazopanib, which promotes the degradation of YAP, has demonstrated beneficial effects in melanoma treatment. A clinical benefit rate of 91% was observed in 58 metastatic melanoma patients receiving pazopanib and paclitaxel [501]. In terms of clinical outcomes, the median overall survival was 12.7 months, and 55% of the 58 patients had stable disease for a duration of 8 weeks or more [501]. Notably, no severe side effects were reported. Although pazopanib shows promise as a treatment for advanced melanoma, further evaluation of this regimen is needed.

### Targeting Notch signaling pathway

RO4929097, a small-molecule blocker of γ-secretase, shows potential benefits for skin cancer treatment (Fig. 4D), although its efficacy remains mixed. In a phase I trial, RO4929097 demonstrated efficacy in treating melanoma patients [502]. This drug showed nearly complete antitumor efficacy against cutaneous metastatic melanoma in one patient. Additionally, individuals with different stages of melanoma maintained stable disease for a minimum of 3 to 6 months post-treatment [502]. RO4929097 was evaluated in phase II trials on melanoma patients in stages IIIB–IV, but these trials (NCT01216787 and NCT01120275) were withdrawn and terminated, respectively. Further evaluation is required to validate the safety and effectiveness of this agent, as well as to investigate other potential targets of the Notch signaling pathway or to consider combination therapies for skin cancer treatment.

### Targeting PI3K/AKT/mTOR and JAK/STAT3 signaling pathway

Several PI3K inhibitors are under evaluation in clinical trials for skin cancer, yielding promising results (Fig. 4E). A trial combining the PI3K/mTOR inhibitor voxtalisib (SAR245409) with the MEK inhibitor pimasertib (NCT01390818) reported a complete response in one melanoma patient. Moreover, 46% of the 110-patient cohort with advanced solid tumors of various types exhibited stable disease with this combination treatment [503]. Another study on triple therapy including the PI3K inhibitor buparlisib, BRAF inhibitor, and MEK inhibitor, showed a low clinical benefit in advanced BRAF V600-mutated melanoma (NCT02159066) [504]. Notably, buparlisib combined with a BRAF inhibitor (NCT01512251) did not yield a well-tolerated response in BRAF V600 melanoma patients [505]. In addition, current and ongoing clinical trials have focused on agents targeting the PI3K/AKT/mTOR pathway in metastatic and BRAF-mutant melanoma. These include the PI3K-beta inhibitor GSK2636771 (NCT03131908), PI3K-gamma inhibitor eganelisib (IPI-549; NCT02637531), and mTORC1 inhibitors such as temsirolimus (CCI-779, NCT00909831 and NCT00022464), and everolimus (RAD001, NCT00591734, NCT00098553, NCT00521001, NCT01014351, and NCT00976573).

AKT inhibitors have shown potential as therapies for advanced and refractory skin cancers, but there are inconsistent data regarding their toxicity and clinical advantages. In a phase I trial, BRAF-wildtype melanoma patients were treated with a combination of the AKT inhibitor uprosertib and trametinib [506]. Disease progression was observed in 43% of 14 patients with melanoma undergoing this combined regimen [506]. Another phase II trial found that no BRAF-wild type melanoma patients achieved a noticeable objective response when treated with the combined uprosertib-trametinib regimen (NCT01941927) [507]**.** Common adverse effects like rash, diarrhea, and mucositis affected the patients’ quality of life with this regimen [506, 507]. However, a SWOG S1221 clinical trial reported that uprosertib combined with dabrafenib or trametinib was well tolerated in melanoma patients [508]. Other AKT inhibitors, such as MK-2206 (NCT00848718 and NCT01480154), ipatasertib (NCT03673787), and afuresertib (NCT01476137), have also been studied in clinical trials targeting advanced melanoma. During a phase I trial, one melanoma patient showed a partial response when MK-2206 was administered alongside carboplatin/paclitaxel, docetaxel, or erlotinib [509]. These combinations were well tolerated, with maculopapular rash and febrile neutropenia being the most common adverse events [509]. A phase I study showed a 55% tumor size reduction in a melanoma patient after 40 weeks of treatment with afuresertib and trametinib [510]. While clinical improvement has been observed with AKT inhibitors, further investigation is required to assess their efficacy and toxicity in skin cancer treatment.

The JAK/STAT3 signaling pathway, crucial in skin cancer progression, presents a potential target for enhancing treatments for advanced skin cancers [117] (Fig. 4E). Inhibitors of JAK and STAT3 have shown strong preclinical antitumor activity against skin cancers. Oral STAT3 inhibitors, such as TTI-101 (NCT03195699) and OPB-31121 (NCT00657176), are currently being assessed in phase I/II clinical trials for their effectiveness and safety. Additionally, modulation of JAK/STAT3 signaling could potentially influence the YAP/TAZ pathway (Fig. 3). However, additional research is needed to evaluate the efficacy and risk profile of STAT3 inhibitors in skin cancer treatment.

## Discussion of treatment challenges and future perspectives related to double-sided niche regulation

The self-renewing epidermis maintains a balance between basal layer proliferation and a precisely regulated differentiation process, forming distinct suprabasal layers, while ensuring SSC preservation. Disruption or dysfunction within the normal SSC niche may serve as a catalyst for skin cancer initiation. The complexity of managing skin cancers arises from the dual functionality of key regulators, which are essential for sustaining both normal SSC and CSC niches (Table 2). Additionally, CSCs can acquire intrinsic resistance while interacting with heterogeneous components of their niche, including critical signaling pathways, adhesion molecules, stromal cells, and immune cells. This multifaceted crosstalk sustains the CSC pool and resistance attributes, including immune evasion. A thorough understanding of how to modulate the components of the CSC niche, in conjunction with the intrinsic resistance mechanisms of CSCs, is essential for devising strategies that can efficiently target and eliminate tumors while sparing the normal developmental processes of SSCs.

### Challenges presented by the shared regulation of signaling pathways in SSC and CSC niches

A major concern when targeting shared regulators of both normal SSC and CSC niches is the potential for treatment failure, as well as the risk of secondary skin cancers arising as adverse outcomes. Notably, the involvement of Shh inhibitors in the formation of secondary SCC is contentious, as existing evidence has failed to demonstrate a clear causal relationship. Nonetheless, various potential pathways have been suggested to elucidate the predisposition to non-BCC secondary cancers following Shh inhibitor treatment [511–513]. It has been reported that among 2,576 patients with BCC treated with vismodegib, there were 197 instances of cutaneous SCC [514]. Invasive SCCs arising from post-BCC clearance with Shh inhibitors harbor new mutations in genes commonly implicated in metastatic SCC, including NOTCH1/2 [515]. Remarkably, these invasive SCCs exhibit a similar mutation rate and approximately 90% genomic identity to the original BCC in a patient treated with vismodegib [513]. These observations imply that mutations in these novel or primary BCC-like genes may drive the squamous transition during Shh pathway blockade. Additionally, SCCs may develop at the same site as previously treated BCCs following Shh inhibitor therapy [513]. Although these cases show SCC-like pathological features, genetic analyses revealed shared tumor drivers between primary BCC and subsequent SCC, suggesting that Shh inhibitors may induce a phenotypic shift from BCC to other skin cancer types [513, 516]. Some alternative oncogenic pathways have been proposed to contribute to the switch in cancer types or exacerbate latent skin cancers following Shh inhibition [481, 517].

The RAS/MAPK pathway has been reported to be activated as an alternative mechanism following Shh pathway inhibition, thereby promoting SCC progression. In Shh inhibitor-resistant BCC or secondary SCCs following BCC treatment, reduced Shh pathway activation is accompanied by increased RAS/MAPK activation [517, 518]. Interestingly, Shh inhibitor-resistant melanoma cell lines show downregulation of Shh-GLI signaling and a shift toward alternative MAPK pathways, such as RAS/ Rapidly Accelerated Fibrosarcoma (RAF)/ERK and JNK/p38 pathways [519], which are also crucial for sustaining CSC stemness and EMT-driven migration in SCC [84, 431, 520]. Additionally, GLI1, a key downstream effector of Shh signaling in BCC, suppresses keratinocyte migration and hyperproliferation driven by EGFR/MEK/ERK signaling [521]. Therefore, inhibition of GLI1 via Shh antagonist treatment may upregulate the EGFR/MEK/ERK signaling pathway, potentially activating the RAS/MAPK pathway in SCC development [521]. Collectively, the potential risks of secondary skin cancers and enhanced tumor invasiveness from Shh pathway inhibitors emphasize the necessity for a deeper understanding of SSC and CSC niches to effectively manage skin cancer.

The dual roles of certain factors in normal SSCs can complicate effective treatment strategies and raise concerns about their unintended effects on healthy tissues. For instance, Notch antagonists have demonstrated minimal antitumor activity in stage IV cutaneous melanoma and are not widely favored in research for skin cancer therapy [522]. Reportedly, unlike Notch1, which promotes the E-cadherin to N-cadherin switch, Notch4 induces opposing changes in melanoma cells during EMT. It reverses this process by repressing the transcription of mesenchymal markers and enhancing E-cadherin expression [523]. Upon activation, Notch4 specifically promotes the expression of HEY1 and HEY2, which suppresses SNAIL2 and TWIST1 transcription by directly binding to their promoter regions [523]. Moreover, Notch is a tumor suppressor in BCC [412] and SCC [414]; however, its activation and DLL1 ligand expression are required for SCC [524] and melanoma progression [453]. DLL1 can promote melanoma expansion through canonical Notch activation or the non-canonical Notch pathway, such as through enhanced tumor cell adhesion or cadherin switch [453, 525]. Interestingly, decreased LFNG levels coincided with elevated expression of DLL1, JAGGED1, and Notch downstream effectors such as HES1 in metastatic melanoma [526]. Reduced LFNG levels in tumor cells may promote Notch trans-activation in adjacent cells through JAGGED ligands by weakening DLL1-Notch1 cis-inhibition and releasing constraints on JAGGED1-Notch1 cis-inhibition [364] (Fig. 1). This change can increase DLL1 ligand availability to activate Notch receptors in adjacent cells [364] or promote TGF-β-mediated tumor spread via SMAD binding [527]. Alternatively, manic and radical fringe enzymes may undergo compensatory changes due to the reduction in LFNG, sustaining high Notch affinity for DLL1 [370]. However, direct evidence connecting reduced LFNG levels with DLL1 activation in melanoma development is still lacking. Collectively, the dual functions of Notch signaling in skin cancers likely contribute to the limited efficacy of Notch inhibitor-based therapies. This underscores the critical need for precise targeting of Notch components or intervention at optimal time points to enhance the effectiveness of skin cancer treatment.

The Wnt signaling pathway can both inhibit and promote melanoma, depending on the context. Given the role of increased Wnt signaling in skin cancer progression, its inhibition has been shown to effectively halt tumor growth [491, 492, 494]. However, recent findings indicate that improving melanoma treatment outcomes may involve the enhancement of Wnt/β-catenin pathway. This can be achieved either by using selective GSK3α/β inhibitors [528] or by increasing Wnt3a levels [529]. The dual roles of Wnt/β-catenin signaling in melanoma likely arise due to shifts between canonical and non-canonical mechanisms. Notably, Wnt5a, which activates Wnt signaling in both canonical and non-canonical processes, is typically upregulated in skin cancers [399]. Wnt5a disrupts the classical Wnt/β-catenin signaling pathway by blocking Wnt3a binding to the FZD2 and LRP6 receptors [530], or by interacting with ROR2 (receptor tyrosine kinase-like orphan receptor 2), either directly or through the upregulation of SIAH2 (Siah E3 ubiquitin protein ligase 2) [455, 531]. These interactions sequester β-catenin in the cytoplasm, promoting its degradation and inhibiting β-catenin-dependent Wnt signaling [455, 530, 531]. Interestingly, the Wnt5a/ROR2 interaction activates the Hippo suppressive pathway of YAP/TAZ signaling, thereby restraining melanoma growth [532]. In contrast, the activation of PKC by non-canonical Wnt5a signaling initiates STAT3 activation, leading to the suppression of MITF expression. This MITF downregulation subsequently suppresses melanocytic differentiation antigens, including MART-1 (melanoma antigen recognized by T-cells 1), while promoting a metastatic, low MITF, stem-like phenotype in invasive melanoma [455]. Additionally, in melanoma cells with highly invasive signatures, increased expression of *WNT5a*, *VEGF*, *TCF4*, and EMT-related genes (fibronectin, *SOX9*, and *MMPs*) is observed, while levels of β-catenin, *LEF1*, *MITF*, E-cadherin, cyclin D1, and c*-MYC* are reduced [162]. In contrast, Wnt3a protein, by activating the canonical Wnt pathway, increases the expression of MAAs such as MART1 and gp100, which helps CTLs target and kill melanoma cells. Furthermore, Wnt3a induces MITF expression in melanoma cells, potentially promoting the differentiation of MSLCs [455, 533]. In addition, β-catenin-induced upregulation of MITF inhibits RHO/ROCK-mediated cell invasion and disrupts the β-catenin-induced expression of MT1-MMP, ultimately reducing tumor invasiveness [534]. These findings suggest that targeting specific Wnt components, including ligands, receptors, and the β-catenin degradation complex, along with the associated signaling pathways, is key to modulating pathway activity and preventing resistance.

### The unique therapeutic targets in the CSC niche for resistant skin cancers

Focusing on components with minimal impact on epidermal homeostasis or those rarely present in the normal SSC niche may help overcome the challenge posed by shared mediators in SSC and CSC niches. For instance, targeting aberrant integrin ligands, such as the γ2 and LG4–5 subunits of LM332, which are absent in the normal SSC niche, shows promise by blocking their integrin binding sites [101]. Antibodies targeting the LM332 α3 chain at the LG4–5 segment effectively induce SCC tumor apoptosis, inhibit tumor cell proliferation, and significantly impair tumorigenesis, while sparing normal tissue adhesion [101]. In addition, the abundant accumulation of the LM332 γ2 chain, coupled with its cleaving enzymes MT1-MMP and MMP-2, is indispensable for the establishment of VM by aggressive melanoma cells [95]. The aberrant cleavage of LM332 γ2 fragments in invasive melanoma generates pro-migratory segments, which significantly enhance VM-associated gene expression [95]. Notably, PI3K facilitates melanoma migration and VM formation by driving MMP-2 and MT1-MMP production, which cleave LM332 γ2 into migratory segments [535]. Intriguingly, inhibiting PI3K with LY294002 significantly impairs the capacity of MSLCs to form VM [535], but also substantially diminishes MT1-MMP and MMP-2 activity, thereby preventing the proteolytic degradation of the LM332 γ2 chain [536]. Therefore, effectively targeting stemness and invasiveness in aggressive skin cancers demands a thorough grasp of the complex relationships among signaling pathways and other components within the CSC niche, including dysregulated ECM elements.

Similarly, integrins like α5β1, αvβ6, αvβ5, α4β1, and αvβ3, which are barely detectable in normal epidermis, are significantly upregulated in CSCs and invasive skin cancers [112, 180, 537–541]. Particularly, integrins α5β1, αvβ5, and αvβ3 are overexpressed in melanoma and contribute to tumor progression. By inhibiting α5β1 [542, 543] or αvβ3 [544], melanoma metastasis is reduced through decreased MMP (MMP-2,-7, and -9) expression and suppression of angiogenesis driven by FGF and VEGF [545]. Additionally, ongoing phase I/II clinical trials are targeting integrin conformations (Fig. 4E), particularly αvβ3 integrin antagonists like MEDI-522 (Etaracizumab, Abegrin, or Vitaxin) (NCT00066196, NCT00111696), Volociximab (α5β1 inhibitor, NCT00099970), and Intetumumab (anti-αv integrin antibody, NCT00246012). These trials have shown a favorable safety profile for targeting key integrins in skin cancers, which are absent in the normal SSC niche, reinforcing their potential for treatment.

### Intrinsic immune evasion mechanisms within CSC niche hinder the effectiveness of immunotherapy

The inherent quiescence of SCs may serve as a defense mechanism, enabling them to evade immune detection. Specifically, long-lived CD34^+^ SSCs located in the HF bulge can escape immune surveillance by T cells [236]. Therefore, CD34^+^ CSCs may resist T cells because of their intrinsically reduced MHC I expression. In contrast, CD34^−^ cells, located outside the bulge, are more vulnerable to CD8^+^ T cell-driven cytotoxicity and show elevated MHC I expression [236]. Notably, CD34^+^ CSCs are increasingly being recognized for their role in enhancing tumorigenicity and conferring resistance to treatments in skin cancers, including BCCs [546], SCCs [547], and melanoma [548]. Investigating the mechanisms that sustain the stemness of CSCs within their niche, particularly those that contribute to immune evasion, could reveal new strategies for improving treatment efficacy in malignant skin cancers.

MSLCs are crucial contributors to the CSC niche and resistance to immunotherapies, including CTL therapies and ICP inhibitors. Their stemness characteristics may provide a means to classify melanoma into distinct immune subtypes, which can help predict how these tumors will respond to immunotherapy. A correlation between stemness and the immune phenotype of melanoma cells was demonstrated through clustering analysis. By analyzing the composition of 28 immune cell populations in the melanoma TME, the study categorized melanoma into three immune groups: high (Im-H), medium (Im-M), and low (Im-L) [549]. The Im-H subtype is distinguished by a pronounced anti-tumor immune landscape and minimal stemness. It also exhibits limited proliferative capacity, enhanced sensitivity to immunotherapy, and favorable clinical prognosis. Conversely, the Im-L subtype is marked by poor anti-tumor immune profiles, elevated stemness, enhanced proliferative potential, and reduced response to immunotherapy [549]. Compared to other subtypes, the Im-H group exhibits higher expression of HLA (Human leukocyte antigen) genes, which are responsible for encoding MHC proteins, as well as elevated scores for both type I and II interferon responses. In contrast to the Im-L group, the Im-H group demonstrates a markedly elevated ratio of immunostimulatory markers, such as CD8^+^ T cells, to immunosuppressive elements, including CD4^+^ Treg cells and M2 macrophages [549]. Notably, melanomas resistant to treatment display significantly augmented stemness attributes and reduced immune scores, particularly in cases treated with ICP inhibitors [549]. The differentiated, proliferative phenotype, and its increased sensitivity to anti-melanoma immunotherapy, are associated with high MITF expression. In contrast, an invasive MSLC phenotype that resists apoptosis and is capable of immune evasion is correlated with low MITF expression [550–552]. This aligns with the reduced MITF levels and upregulation of the mesenchymal marker TCF4 observed in ICP therapy non-responders [552]. These findings indicate that the inherent traits of CSCs, along with the CSC niche that sustains their stemness, are critical factors in contributing to resistance to immunotherapy.

### Potential treatments targeting factors in the CSC niche sustaining immunoresistance

Enhanced IFNγ expression in the CSC niche can sensitize tumor cells to immunotherapy. During the telogen phase, HFSCs show low expression of interferon gamma receptor genes such as *IFNGR1*, which impedes the ability of activated T cells to upregulate MHC I levels through IFNγ production [236]. This lack of IFNγ responsiveness is consistent with the observation that reduced IFNγ levels correlate with a poorer prognosis in skin cancers. CD8^+^ T cell infiltration and SCC regression were not observed when IFNγ activity was blocked [553]. In addition, a case of early mortality due to disseminated and recurrent SCC was reported in a patient with a deficiency in IFNγR2 [554]. Conversely, elevated IFNγ levels in the TME correlate with BCC tumor regression [555]. Treatment with IFNγ serves as an immuno-sensitizer, enhancing MHC-dependent antigen presentation across various cell types, including melanoma and cutaneous SCC cells [553, 556]. Additionally, IFNγ increases MHC I expression and enhances CD8^+^ T cell migration into SCCs by upregulating chemokines such as CXCL10 and CCL5 [553]. Systemic IFNγ administration induced MHC I expression in MHC I-negative melanomas, as demonstrated in a phase II trial [556]. In a phase Ib/II trial, IFNγ supplementation was shown to improve the effectiveness of checkpoint blockade therapy in melanoma patients [557]. Melanoma pretreatment with low-dose IFNγ enhances tumor cell sensitivity to CD8^+^ and CD4^+^ T cell-mediated destruction [558]. This suggests that combining IFNγ with adoptive cell therapy (ACT) could enhance treatment efficacy, offering a promising approach for metastatic melanoma. However, IFNγ is considered a double-edged sword due to its contrasting effects on tumor immunity [558, 559]. While IFNγ enhances tumor cell recognition by upregulating MHC I and II, crucial for effective CTL-mediated killing, it also induces the upregulation of immunosuppressive factors like PD-L1 and IDO (indoleamine 2,3-dioxygenase) in melanoma cells [558]. Notably, transferred T cells that overexpress PD-1 following IFNγ treatment may dampen the immune response, ultimately limiting the effectiveness of immunotherapy [559]. In addition, IFNγ-induced PD-L1 upregulation can trigger dedifferentiation in melanoma cells, which is characterized by reduced MITF expression, increased SOX10 expression, and elevated PD-L1 levels [560, 561]. Through dedifferentiation, IFNγ induces higher expression of immunomodulatory genes, upregulates PD-L1 protein, and promotes cytokine secretion [561]. To enhance therapeutic efficacy, it is crucial to identify and target the various cellular pathways involved in CSC immunogenicity, thereby achieving a more precise and effective immune response.

Integrating targets involved in key signaling pathways in the CSC niche with immunotherapy could enhance treatment strategies for skin cancers. For example, by upregulating MHC I expression and boosting CD4^+^ and CD8^+^ T cell infiltration in BCC tumors [562], Shh inhibitors may enhance adaptive immune responses, thereby potentially improving the effectiveness of anti-PD-1 therapy and increasing tumor response rates [563]. Moreover, PD-L1 expression is elevated in tumor cells and lymphocytes in BCC after treatment with Shh inhibitors [564]. As a result, combining Shh inhibitors with PD-L1 inhibitors could provide a promising strategy for advanced BCCs, potentially overcoming the immune resistance caused by increased PD-L1 expression. Anti-tumor T-cell activity is enhanced by cemiplimab, an FDA-approved treatment for adults with advanced or metastatic BCC that failed or cannot tolerate Shh inhibitors [565]. This IgG4 human monoclonal antibody prevents PD-L1 and PD-L2 ligands from binding to the PD-1 receptor. In clinical trials, cemiplimab induced durable responses and exhibited substantial anti-tumor activity in metastatic BCC patients with disease progression or those unable to tolerate Shh inhibitors. A phase II multi-center study (NCT03132636) examined cemiplimab efficacy in 54 mBCC and 84 laBCC patients with prior Shh inhibitor failure or intolerance. The trial showed that cemiplimab yielded clinically meaningful anti-tumor activity and was well-tolerated in these patients. Notably, complete and partial responses were recorded in 5 patients (6%) and 21 patients (25%) experiencing laBCC, respectively [566]. Among the 84 laBCC patients, 40 (48%) experienced emergent side effects with grade 3–4, most commonly colitis and hypertension (both in 5% of patients). Progression-free survival in 54 mBCC patients ranged from 4 to 16 months, with a disease control rate of 63%, according to independent central examination [567]. Importantly, no fatalities were attributed to treatment in this phase II trial [566, 567]. However, a phase II trial comparing the combination of anti-PD-1 pembrolizumab with vismodegib to pembrolizumab monotherapy found no substantial enhancement in response rate [568]. The overall response rate for pembrolizumab monotherapy (44% in 9 patients) surpassed that of the combination with vismodegib (29% in 7 patients). These results imply that targeting the signaling pathway and immunotherapy remain challenging in skin cancer treatment.

In metastatic melanoma, combining Wnt/β-catenin antagonists with ICP inhibitors (anti-PD-1 pembrolizumab and anti-CTLA-4 ipilimumab) failed to yield a substantial therapeutic benefit in metastatic melanoma, while concomitantly increasing the risk of adverse effects [569]. Aspirin functions both as a Wnt/β-catenin inhibitor, promoting β-catenin sequestration via GSK3β, and as an anti-inflammatory agent by inhibiting COX enzymes [494]. Therefore, this combination may confer dual benefits in enhancing anti-PD-1 efficacy for several reasons. Reportedly, Wnt/β-catenin activation in melanoma cells impedes anti-PD-L1 efficacy by blocking T-cell recruitment into the TME. Mechanistically, when β-catenin is activated in melanoma cells, ATF3-mediated repression of CCL4 hinders T-cell infiltration in the melanoma microenvironment [570]. Upregulated Wnt ligands (Wnt4 and Wnt5a) and positive modulators (DKK2 and SFRP2) were detected in non-responders to anti-PD-L1 therapy [571]. Additionally, COX2 suppression by aspirin can impair the synthesis of prostaglandin E2 [494], which is associated with a reduced accumulation of exhausted CD8^+^ T cells, marked by TIM3 (T-cell immunoglobulin and mucin-domain containing-3) expression, in the melanoma TME [572]. A retrospective analysis showed that combining ICP inhibitors (anti-CTLA-4 and/or anti-PD-1) with COX inhibitors (aspirin or selective COX2 inhibitors) significantly improved the overall response rate at 6 months in melanoma patients, compared to ICP monotherapy. Among 90 melanoma patients, combination therapy achieved an overall response rate of 58.6%, compared to 19.2% with ICP monotherapy (*p* = 0.0005) [573]. However, the trial (NCT03396952) examining the combination of aspirin and ICP inhibitors in melanoma has not yielded significant clinical benefits in metastatic melanoma [569]. This trial reported a 62.9% objective response rate at 12 weeks in 27 patients [569], comparable to the 55%−61% observed at approximately 16 months in a clinical study using exclusive ICP treatment [574]. In addition, a study found that PD-L1 expression inversely correlated with Wnt/β-catenin signaling activity in melanoma tumors [575]. These findings suggest the presence of alternative mechanisms within the CSC niche, as well as in CSCs or tumor cells, which may sustain resistance to therapy after blocking oncogenic signaling pathways such as Wnt and Shh. Integrating immunotherapy and modulating other signaling pathways or CSC features associated with immune evasion could enhance therapeutic efficacy in refractory skin cancers.

Currently, phase I/II clinical trials (NCT03673787, NCT02637531, NCT03131908) are investigating anti-PD-1/PD-L1 agents in combination with PI3K/AKT inhibitors for advanced melanoma. Additionally, targeting the YAP pathway has demonstrated potential in mitigating tumor-induced immune evasion. Melan-A-specific CD8^+^ T cells failed to attack BRAFi-resistant melanoma cells with high nuclear YAP accumulation, which upregulated PD-L1 expression [576]. Anti-PD-1 treatment or YAP depletion can mitigate T cell dysfunction and PD-L1 upregulation mediated by YAP in melanoma cells [576]. Therefore, further investigation into how signaling pathways and immune responses interact within the CSC niche may uncover new combination strategies to overcome resistance in skin cancers.

### Shared signaling pathways in the CSC niche and tumor immunosuppression

Signaling pathway activation or specific protein expression in CSCs, which overlap with those found in immunosuppressive cells such as CAFs and TAMs, may elevate the likelihood of treatment resistance in skin cancers. A clinical trial with cilengitide has failed to show survival benefits in metastatic melanoma patients [577]. Integrin β3 expression on TAMs contributes to cilengitide-mediated tumor-promoting effects. This may be attributed to their role in suppressing the tumor-promoting functions of TAMs [578]. Integrin β3 in macrophages increases the M1/M2 macrophage ratio in the TME by activating STAT1 and suppressing STAT6 [578]. Therefore, inhibiting β3 on TAMs can accelerate tumor growth, promote TAM accumulation, and diminish CD8^+^ T cell infiltration. Conversely, targeting macrophages can reduce the unexpected supportive effects of anti-integrin β3 treatment on tumor progression [578]. Melanoma resistance to integrin αvβ3 inhibitors is influenced by these opposing roles of integrin β3 in TAMs and MSLC. Therefore, a combination treatment of anti-macrophage might help reduce cancer resistance.

The plasticity of metastatic melanoma cells is regulated by Notch1 signaling in CAFs. Melanosphere formation and the acquisition of Nestin- and CD271-positive melanoma stem/initiating cell phenotypes are suppressed by CAFs with Notch1 hyperactivation [579]. In contrast, Notch1-silenced CAFs significantly expand the MSLC population by upregulating stemness markers such as SOX2, OCT4, and NANOG [579]. Moreover, Notch1 preserves the stemness, oncogenic properties, and metastatic potential of MSLCs [321, 325]. Therefore, the divergent effects of Notch1 signaling in CAFs and MSLCs may attenuate the therapeutic efficacy of targeting the Notch pathway.

Gaining a comprehensive understanding of how core signaling pathways are intricately regulated within the CSC niche is crucial for effectively influencing CSC behavior. Targeting these pathways, in combination with other niche components, could offer a more efficient strategy to eradicate CSC populations in skin cancers.

## Conclusion

The niche microenvironment plays a crucial role in regulating the fate of normal SSCs and CSCs. Adhesive molecules within the niche are essential for normal epidermal differentiation; however, their excessive and abnormal expression can lead to the expansion of CSCs. The dual roles of key components of the adhesive niche—including the dermo-epidermal junction, adherens junctions, various cell types (especially immune cells and fibroblasts), and major signaling pathways such as Shh, Wnt/β-catenin, YAP/TAZ, and Notch—are critical for maintaining the balanced growth of SSCs. While their aberrant activation contributes to skin tumor progression and CSC formation, targeting specific signaling pathways regulated by the niche holds the potential for treating skin cancers with CSC properties and drug resistance, both in clinical practice and ongoing clinical trials. Nonetheless, the precise relationship between CSCs and their niche remains partially understood in certain contexts, and cases of resistance have been associated with the incomplete eradication of CSCs. Further research is needed to investigate the reciprocal interactions between cutaneous CSCs and their niche, which could provide valuable insights for addressing malignancy and drug resistance in skin cancers.

## Data Availability

No datasets were generated or analysed during the current study.

## Abbreviations

SSCs: Skin stem cells
TACs: Transient-amplifying cells
SCs: Stem cells
NMSC: Nonmelanoma skin cancer
SCC: Squamous cell carcinoma
CSCs: Cancer stem cells
IFESCs: Interfollicular epidermal stem cells
HFSCs: Hair follicle stem cells
BM: Basement membrane
ECM: Extracellular matrix
LMs: Laminins
EGFR: Epidermal growth factor receptor
FAs: Focal adhesions
TGF: Transforming growth factor
Wnt: Wingless-related integration site
BCC: Basal cell carcinoma
MMPs: Matrix metalloproteinases
MAPK/ERK: Mitogen-activated protein kinase/Extracellular signal-regulated kinase
RAC1: Rac family small GTPase 1
CDC42: Cell division cycle 42
RAS: Rat sarcoma virus
PI3K/AKT/mTOR: Phosphoinositide 3-kinase/Protein kinase B (Akt)/Mammalian target of rapamycin
FAK: Focal adhesion kinase
RHOA/ROCK: Ras homolog family member A/ Rho-associated protein kinase
JAK/STAT3: Janus kinase/ Signal transducer and activator of transcription 3
EMT: Epithelial–mesenchymal transition
AJs: Adherens junctions
YAP/TAZ: Yes-associated protein/transcriptional co-activator with pdz-binding motif
CAFs: Cancer-associated fibroblasts
TAMs: Tumor-associated macrophages
TANs: Tumor-associated neutrophils
SHH: Sonic hedgehog
MSCs: Melanocyte stem cell
DLL1: Delta-like ligand 1
LFNG: Lunatic Fringe

## References

[1] Blanpain C, Fuchs E. Epidermal homeostasis: a balancing act of stem cells in the skin. Nat Rev Mol Cell Biol. 2009;10:207–17. 19209183 10.1038/nrm2636 PMC2760218

[2] Zhang W, Zeng W, Jiang A, He Z, Shen X, Dong X, et al. Global, regional and national incidence, mortality and disability-adjusted life-years of skin cancers and trend analysis from 1990 to 2019: An analysis of the global burden of disease study 2019. Cancer Med. 2021;10:4905–22. 34105887 10.1002/cam4.4046 PMC8290243

[3] Chen C, Wang Z, Qin YR. Health-related quality of life in stage III-IV melanoma treated with targeted therapy or immunotherapy: A systematic review on the adequacy of reporting and clinical issues in phase III randomized controlled trials. Cancer Med. 2023;12:2262–80. 36030506 10.1002/cam4.5183 PMC9939121

[4] Bray F, Laversanne M, Sung H, Ferlay J, Siegel RL, Soerjomataram I, et al. Global cancer statistics 2022: GLOBOCAN estimates of incidence and mortality worldwide for 36 cancers in 185 countries. CA Cancer J Clin. 2024;74:229–63. 38572751 10.3322/caac.21834

[5] Hamp A, Anderson J, Sivesind TE, Szeto MD, Hadjinicolaou A. From the Cochrane library: systemic treatments for metastatic cutaneous melanoma. JMIR Dermatol. 2021;4: e30270. 37632820 10.2196/30270 PMC10334959

[6] Wang M, Gao X, Zhang L. Recent global patterns in skin cancer incidence, mortality, and prevalence. Chin Med J (Engl). 2025;138:185–92. 39682020 10.1097/CM9.0000000000003416 PMC11745855

[7] Arnold M, Singh D, Laversanne M, Vignat J, Vaccarella S, Meheus F, et al. Global burden of cutaneous melanoma in 2020 and projections to 2040. JAMA Dermatol. 2022;158:495–503. 35353115 10.1001/jamadermatol.2022.0160 PMC8968696

[8] Leiter U, Keim U, Garbe C, Epidemiology of skin cancer: update,. In Sunlight, Vitamin D and Skin. Cancer. 2019;2020:123–39. 10.1007/978-3-030-46227-7_6 32918216

[9] Eide MJ, Krajenta R, Johnson D, Long JJ, Jacobsen G, Asgari MM, et al. Identification of patients with nonmelanoma skin cancer using health maintenance organization claims data. Am J Epidemiol. 2010;171:123–8. 19969529 10.1093/aje/kwp352 PMC2796985

[10] Hillen U, Leiter U, Haase S, Kaufmann R, Becker J, Gutzmer R, et al. Advanced cutaneous squamous cell carcinoma: A retrospective analysis of patient profiles and treatment patterns-Results of a non-interventional study of the DeCOG. Eur J Cancer. 2018;96:34–43. 29665511 10.1016/j.ejca.2018.01.075

[11] Guo A, Liu X, Li H, Cheng W, Song Y. The global, regional, national burden of cutaneous squamous cell carcinoma (1990–2019) and predictions to 2035. Eur J Cancer Care. 2023;2023:5484597.

[12] Biddle A, Liang X, Gammon L, Fazil B, Harper LJ, Emich H, et al. Cancer stem cells in squamous cell carcinoma switch between two distinct phenotypes that are preferentially migratory or proliferative. Cancer Res. 2011;71:5317–26. 21685475 10.1158/0008-5472.CAN-11-1059

[13] El-Khattouti A, Selimovic D, Haïkel Y, Megahed M, Gomez CR, Hassan M. Identification and analysis of CD133+ melanoma stem-like cells conferring resistance to taxol: An insight into the mechanisms of their resistance and response. Cancer Lett. 2014;343:123–33. 24080340 10.1016/j.canlet.2013.09.024

[14] Schatton T, Murphy GF, Frank NY, Yamaura K, Waaga-Gasser AM, Gasser M, et al. Identification of cells initiating human melanomas. Nature. 2008;451:345–9. 18202660 10.1038/nature06489 PMC3660705

[15] Luo Y, Dallaglio K, Chen Y, Robinson WA, Robinson SE, McCarter MD, et al. ALDH1A isozymes are markers of human melanoma stem cells and potential therapeutic targets. Stem Cells. 2012;30:2100–13. 22887839 10.1002/stem.1193 PMC3448863

[16] Pinc A, Somasundaram R, Wagner C, Hörmann M, Karanikas G, Jalili A, et al. Targeting CD20 in melanoma patients at high risk of disease recurrence. Mol Ther. 2012;20:1056–62. 22354376 10.1038/mt.2012.27 PMC3345981

[17] Civenni G, Walter A, Kobert N, Mihic-Probst D, Zipser M, Belloni B, et al. Human CD271-positive melanoma stem cells associated with metastasis establish tumor heterogeneity and long-term growth. Cancer Res. 2011;71:3098–109. 21393506 10.1158/0008-5472.CAN-10-3997

[18] Shakhova O, Zingg D, Schaefer SM, Hari L, Civenni G, Blunschi J, et al. Sox10 promotes the formation and maintenance of giant congenital naevi and melanoma. Nat Cell Biol. 2012;14:882–90. 22772081 10.1038/ncb2535

[19] Schober M, Fuchs E. Tumor-initiating stem cells of squamous cell carcinomas and their control by TGF-β and integrin/focal adhesion kinase (FAK) signaling. Proc Natl Acad Sci U S A. 2011;108:10544–9. 21670270 10.1073/pnas.1107807108 PMC3127891

[20] Colmont CS, BenKetah A, Reed SH, Hawk NV, Telford WG, Ohyama M, et al. CD200-expressing human basal cell carcinoma cells initiate tumor growth. Proc Natl Acad Sci U S A. 2013;110:1434–9. 23292936 10.1073/pnas.1211655110 PMC3557049

[21] Guasch G, The epithelial stem cell niche in skin, in Biology and engineering of stem cell niches, A.a.K. Vishwakarma, J.M., Editor. 2017, Elsevier. p. 127–143.

[22] Gonzales KAU, Fuchs E. Skin and its regenerative powers: an alliance between stem cells and their niche. Dev Cell. 2017;43:387–401. 29161590 10.1016/j.devcel.2017.10.001 PMC5797699

[23] Box NF, Torchia EC, Roop DR. Are stem cell niches shared for skin cancers? Pigment Cell Melanoma Res. 2010;23:517–20. 20546533 10.1111/j.1755-148X.2010.00727.x PMC3137929

[24] Hsu Y-C, Li L, Fuchs E. Emerging interactions between skin stem cells and their niches. Nat Med. 2014;20:847–56. 25100530 10.1038/nm.3643 PMC4358898

[25] Boukamp P. Non-melanoma skin cancer: what drives tumor development and progression? Carcinogenesis. 2005;26:1657–67. 15905207 10.1093/carcin/bgi123

[26] Hasan N, Nadaf A, Imran M, Jiba U, Sheikh A, Almalki WH, et al. Skin cancer: understanding the journey of transformation from conventional to advanced treatment approaches. Mol Cancer. 2023;22:168. 37803407 10.1186/s12943-023-01854-3 PMC10559482

[27] Kumar D, Gorain M, Kundu G, Kundu GC. Therapeutic implications of cellular and molecular biology of cancer stem cells in melanoma. Mol Cancer. 2017;16:7. 28137308 10.1186/s12943-016-0578-3 PMC5282877

[28] Debaugnies M, Sánchez-Danés A, Rorive S, Raphaël M, Liagre M, Parent MA, et al. YAP and TAZ are essential for basal and squamous cell carcinoma initiation. EMBO Rep. 2018;19: e45809. 29875149 10.15252/embr.201845809 PMC6030692

[29] Pei G, Lan Y, Chen D, Ji L, Hua ZC. FAK regulates E-cadherin expression via p-SrcY416/p-ERK1/2/p-Stat3Y705 and PPARγ/miR-125b/Stat3 signaling pathway in B16F10 melanoma cells. Oncotarget. 2017;8:13898–908. 28108732 10.18632/oncotarget.14687 PMC5355148

[30] Blanpain C, Simons BD. Unravelling stem cell dynamics by lineage tracing. Nat Rev Mol Cell Biol. 2013;14:489–502. 23860235 10.1038/nrm3625

[31] Hsu Y-C, Pasolli HA, Fuchs E. Dynamics between stem cells, niche, and progeny in the hair follicle. Cell. 2011;144:92–105. 21215372 10.1016/j.cell.2010.11.049 PMC3050564

[32] Ito M, Liu Y, Yang Z, Nguyen J, Liang F, Morris RJ, et al. Stem cells in the hair follicle bulge contribute to wound repair but not to homeostasis of the epidermis. Nat Med. 2005;11:1351–4. 16288281 10.1038/nm1328

[33] Brownell I, Guevara E, Bai CB, Loomis CA, Joyner AL. Nerve-derived sonic hedgehog defines a niche for hair follicle stem cells capable of becoming epidermal stem cells. Cell Stem Cell. 2011;8:552–65. 21549329 10.1016/j.stem.2011.02.021 PMC3089905

[34] Dupin E, Le Douarin NM. Development of melanocyte precursors from the vertebrate neural crest. Oncogene. 2003;22:3016–23. 12789276 10.1038/sj.onc.1206460

[35] Rabbani P, Takeo M, Chou W, Myung P, Bosenberg M, Chin L, et al. Coordinated activation of Wnt in epithelial and melanocyte stem cells initiates pigmented hair regeneration. Cell. 2011;145:941–55. 21663796 10.1016/j.cell.2011.05.004 PMC3962257

[36] Lapouge G, Youssef KK, Vokaer B, Achouri Y, Michaux C, Sotiropoulou PA, et al. Identifying the cellular origin of squamous skin tumors. Proc Natl Acad Sci U S A. 2011;108:7431–6. 21502497 10.1073/pnas.1012720108 PMC3088632

[37] Becker JC and zur Hausen A. Cells of origin in skin cancer. J Invest Dermatol. 2014; 134:2491–2493. 10.1038/jid.2014.233 25219650

[38] Roig-Rosello E, Rousselle P. The human epidermal basement membrane: A shaped and cell instructive platform that aging slowly alters. Biomolecules. 2020;10:1607. 33260936 10.3390/biom10121607 PMC7760980

[39] Morgner J, Ghatak S, Jakobi T, Dieterich C, Aumailley M, Wickström SA. Integrin-linked kinase regulates the niche of quiescent epidermal stem cells. Nat Commun. 2015;6:1–15. 10.1038/ncomms9198 PMC4569844 26349061

[40] Yamada T, Hasegawa S, Miyachi K, Date Y, Inoue Y, Yagami A, et al. Laminin-332 regulates differentiation of human interfollicular epidermal stem cells. Mech Ageing Dev. 2018;171:37–46. 29555367 10.1016/j.mad.2018.03.007

[41] Margadant C, Charafeddine RA, Sonnenberg A. Unique and redundant functions of integrins in the epidermis. FASEB J. 2010;24:4133–52. 20624931 10.1096/fj.09-151449

[42] Huveneers S, Danen EHJ. Adhesion signaling – crosstalk between integrins. Src and Rho J Cell Sci. 2009;122:1059–69. 19339545 10.1242/jcs.039446

[43] Carter WG, Kaur P, Gil SG, Gahr PJ, Wayner EA. Distinct functions for integrins alpha 3 beta 1 in focal adhesions and alpha 6 beta 4/bullous pemphigoid antigen in a new stable anchoring contact (SAC) of keratinocytes: relation to hemidesmosomes. J Cell Biol. 1990;111:3141–54. 2269668 10.1083/jcb.111.6.3141 PMC2116384

[44] Nishiuchi R, Murayama O, Fujiwara H, Gu J, Kawakami T, Aimoto S, et al. Characterization of the ligand-binding specificities of integrin alpha3beta1 and alpha6beta1 using a panel of purified laminin isoforms containing distinct alpha chains. J Biochem. 2003;134:497–504. 14607975 10.1093/jb/mvg185

[45] Kaur P, Li A. Adhesive properties of human basal epidermal cells: an analysis of keratinocyte stem cells, transit amplifying cells, and postmitotic differentiating cells. J Invest Dermatol. 2000;114:413–20. 10692098 10.1046/j.1523-1747.2000.00884.x

[46] Jones PH, Harper S, Watt FM. Stem cell patterning and fate in human epidermis. Cell. 1995;80:83–93. 7813021 10.1016/0092-8674(95)90453-0

[47] Rousselle P, Beck K. Laminin 332 processing impacts cellular behavior. Cell Adh Migr. 2013;7:122–34. 23263634 10.4161/cam.23132 PMC3544776

[48] Tsubota Y, Yasuda C, Kariya Y, Ogawa T, Hirosaki T, Mizushima H, et al. Regulation of biological activity and matrix assembly of laminin-5 by COOH-terminal, LG4-5 domain of alpha3 chain. J Biol Chem. 2005;280:14370–7. 15695818 10.1074/jbc.M413051200

[49] Goldfinger LE, Stack MS, Jones JC. Processing of laminin-5 and its functional consequences: role of plasmin and tissue-type plasminogen activator. J Cell Biol. 1998;141:255–65. 9531563 10.1083/jcb.141.1.255 PMC2132728

[50] Behrens DT, Villone D, Koch M, Brunner G, Sorokin L, Robenek H, et al. The epidermal basement membrane is a composite of separate laminin- or collagen IV-containing networks connected by aggregated perlecan, but not by nidogens. J Biol Chem. 2012;287:18700–9. 22493504 10.1074/jbc.M111.336073 PMC3365761

[51] Kariya Y, Sato H, Katou N, Kariya Y, Miyazaki K. Polymerized laminin-332 matrix supports rapid and tight adhesion of keratinocytes, suppressing cell migration. PLoS ONE. 2012;7: e35546. 22563463 10.1371/journal.pone.0035546 PMC3341393

[52] Tsuruta D, Hashimoto T, Hamill KJ, Jones JC. Hemidesmosomes and focal contact proteins: functions and cross-talk in keratinocytes, bullous diseases and wound healing. J Dermatol Sci. 2011;62:1–7. 21376539 10.1016/j.jdermsci.2011.01.005 PMC4492441

[53] Koshikawa N, Giannelli G, Cirulli V, Miyazaki K, Quaranta V. Role of cell surface metalloprotease MT1-MMP in epithelial cell migration over laminin-5. J Cell Biol. 2000;148:615–24. 10662785 10.1083/jcb.148.3.615 PMC2174802

[54] Koshikawa N, Minegishi T, Sharabi A, Quaranta V, Seiki M. Membrane-type matrix metalloproteinase-1 (MT1-MMP) is a processing enzyme for human laminin gamma 2 chain. J Biol Chem. 2005;280:88–93. 15525652 10.1074/jbc.M411824200

[55] Schenk S, Hintermann E, Bilban M, Koshikawa N, Hojilla C, Khokha R, et al. Binding to EGF receptor of a laminin-5 EGF-like fragment liberated during MMP-dependent mammary gland involution. J Cell Biol. 2003;161:197–209. 12695504 10.1083/jcb.200208145 PMC2172889

[56] Margadant C, Frijns E, Wilhelmsen K, Sonnenberg A. Regulation of hemidesmosome disassembly by growth factor receptors. Curr Opin Cell Biol. 2008;20:589–96. 18583123 10.1016/j.ceb.2008.05.001

[57] Frijns E, Sachs N, Kreft M, Wilhelmsen K, Sonnenberg A. EGF-induced MAPK signaling inhibits hemidesmosome formation through phosphorylation of the integrin beta4. J Biol Chem. 2010;285:37650–62. 20870721 10.1074/jbc.M110.138818 PMC2988370

[58] Mainiero F, Pepe A, Wary KK, Spinardi L, Mohammadi M, Schlessinger J, et al. Signal transduction by the alpha 6 beta 4 integrin: distinct beta 4 subunit sites mediate recruitment of Shc/Grb2 and association with the cytoskeleton of hemidesmosomes. EMBO J. 1995;14:4470–81. 7556090 10.1002/j.1460-2075.1995.tb00126.x PMC394539

[59] Mainiero F, Murgia C, Wary KK, Curatola AM, Pepe A, Blumemberg M, et al. The coupling of alpha6beta4 integrin to Ras-MAP kinase pathways mediated by Shc controls keratinocyte proliferation. EMBO J. 1997;16:2365–75. 9171350 10.1093/emboj/16.9.2365 PMC1169837

[60] Dans M, Gagnoux-Palacios L, Blaikie P, Klein S, Mariotti A, Giancotti FG. Tyrosine phosphorylation of the beta 4 integrin cytoplasmic domain mediates Shc signaling to extracellular signal-regulated kinase and antagonizes formation of hemidesmosomes. J Biol Chem. 2001;276:1494–502. 11044453 10.1074/jbc.M008663200

[61] Benitah SA, Frye M, Glogauer M, Watt FM. Stem cell depletion through epidermal deletion of Rac1. Science. 2005;309:933–5. 16081735 10.1126/science.1113579

[62] Frye M, Gardner C, Li ER, Arnold I, Watt FM. Evidence that Myc activation depletes the epidermal stem cell compartment by modulating adhesive interactions with the local microenvironment. Development. 2003;130:2793–808. 12736221 10.1242/dev.00462

[63] Gebhardt A, Frye M, Herold S, Benitah SA, Braun K, Samans B, et al. Myc regulates keratinocyte adhesion and differentiation via complex formation with Miz1. J Cell Biol. 2006;172:139–49. 16391002 10.1083/jcb.200506057 PMC2063541

[64] Noethel B, Ramms L, Dreissen G, Hoffmann M, Springer R, Rübsam M, et al. Transition of responsive mechanosensitive elements from focal adhesions to adherens junctions on epithelial differentiation. Mol Biol Cell. 2018;29:2317–25. 30044710 10.1091/mbc.E17-06-0387 PMC6249805

[65] Stutchbury B, Atherton P, Tsang R, Wang DY, Ballestrem C. Distinct focal adhesion protein modules control different aspects of mechanotransduction. J Cell Sci. 2017;130:1612–24. 28302906 10.1242/jcs.195362 PMC5450230

[66] Gonzales M, Haan K, Baker SE, Fitchmun M, Todorov I, Weitzman S, et al. A cell signal pathway involving laminin-5, alpha3beta1 integrin, and mitogen-activated protein kinase can regulate epithelial cell proliferation. Mol Biol Cell. 1999;10:259–70. 9950675 10.1091/mbc.10.2.259 PMC25167

[67] Choma DP, Milano V, Pumiglia KM, DiPersio CM. Integrin alpha3beta1-dependent activation of FAK/Src regulates Rac1-mediated keratinocyte polarization on laminin-5. J Invest Dermatol. 2007;127:31–40. 16917494 10.1038/sj.jid.5700505

[68] Zhu AJ, Haase I, Watt FM. Signaling via beta1 integrins and mitogen-activated protein kinase determines human epidermal stem cell fate in vitro. Proc Natl Acad Sci U S A. 1999;96:6728–33. 10359780 10.1073/pnas.96.12.6728 PMC21983

[69] Freije A, Molinuevo R, Ceballos L, Cagigas M, Alonso-Lecue P, Rodriguez R, et al. Inactivation of p53 in human keratinocytes leads to squamous differentiation and shedding via replication stress and mitotic slippage. Cell Rep. 2014;9:1349–60. 25453755 10.1016/j.celrep.2014.10.012

[70] Kurata S-i, Okuyama T, Osada M, Watanabe T, Tomimori Y, Sato S, et al. p51/p63 controls subunit α3 of the major epidermis integrin anchoring the stem cells to the niche*.* J Biol Chem. 2004; 279:50069–50077. 10.1074/jbc.M406322200 15361520

[71] Okuyama R, Ogawa E, Nagoshi H, Yabuki M, Kurihara A, Terui T, et al. p53 homologue, p51/p63, maintains the immaturity of keratinocyte stem cells by inhibiting Notch1 activity. Oncogene. 2007;26:4478–88. 17237812 10.1038/sj.onc.1210235

[72] Russell AJ, Fincher EF, Millman L, Smith R, Vela V, Waterman EA, et al. Alpha 6 beta 4 integrin regulates keratinocyte chemotaxis through differential GTPase activation and antagonism of alpha 3 beta 1 integrin. J Cell Sci. 2003;116:3543–56. 12865436 10.1242/jcs.00663

[73] Schlaepfer DD, Mitra SK. Multiple connections link FAK to cell motility and invasion. Curr Opin Genet Dev. 2004;14:92–101. 15108811 10.1016/j.gde.2003.12.002

[74] Hamelers IH, Olivo C, Mertens AE, Pegtel DM, van der Kammen RA, Sonnenberg A, et al. The Rac activator Tiam1 is required for (alpha)3(beta)1-mediated laminin-5 deposition, cell spreading, and cell migration. J Cell Biol. 2005;171:871–81. 16330714 10.1083/jcb.200509172 PMC2171282

[75] Miao H, Li S, Hu YL, Yuan S, Zhao Y, Chen BP, et al. Differential regulation of Rho GTPases by beta1 and beta3 integrins: the role of an extracellular domain of integrin in intracellular signaling. J Cell Sci. 2002;115:2199–206. 11973360 10.1242/jcs.115.10.2199

[76] Nguyen BP, Gil SG, Carter WG. Deposition of laminin 5 by keratinocytes regulates integrin adhesion and signaling. J Biol Chem. 2000;275:31896–907. 10926936 10.1074/jbc.M006379200

[77] Zhou H, Kramer RH. Integrin engagement differentially modulates epithelial cell motility by RhoA/ROCK and PAK1. J Biol Chem. 2005;280:10624–35. 15611088 10.1074/jbc.M411900200

[78] Brakebusch C, Fässler R. The integrin-actin connection, an eternal love affair. EMBO J. 2003;22:2324–33. 12743027 10.1093/emboj/cdg245 PMC156003

[79] Wegner J, Loser K, Apsite G, Nischt R, Eckes B, Krieg T, et al. Laminin α5 in the keratinocyte basement membrane is required for epidermal-dermal intercommunication. Matrix Biol. 2016;56:24–41. 27234307 10.1016/j.matbio.2016.05.001

[80] Iriyama S, Yasuda M, Nishikawa S, Takai E, Hosoi J, Amano S. Decrease of laminin-511 in the basement membrane due to photoaging reduces epidermal stem/progenitor cells. Sci Rep. 2020;10:1–10. 32724130 10.1038/s41598-020-69558-y PMC7387558

[81] Katayama S, Koga K, Fujimoto M, Matsuzaki I, Nabeshima K, Imafuku S, et al. Expression of laminin332 γ2 at the invasive front is associated with tumor budding and poor prognosis in cutaneous squamous cell carcinoma. J Dermatol. 2023;50:1585–93. 37752805 10.1111/1346-8138.16952

[82] Siljamäki E, Rappu P, Riihilä P, Nissinen L, Kähäri VM, Heino J. H-Ras activation and fibroblast-induced TGF-β signaling promote laminin-332 accumulation and invasion in cutaneous squamous cell carcinoma. Matrix Biol. 2020;87:26–47. 31655292 10.1016/j.matbio.2019.09.001

[83] Oikawa Y, Hansson J, Sasaki T, Rousselle P, Domogatskaya A, Rodin S, et al. Melanoma cells produce multiple laminin isoforms and strongly migrate on α5 laminin(s) via several integrin receptors. Exp Cell Res. 2011;317:1119–33. 21195710 10.1016/j.yexcr.2010.12.019

[84] Dajee M, Lazarov M, Zhang JY, Cai T, Green CL, Russell AJ, et al. NF-kappaB blockade and oncogenic Ras trigger invasive human epidermal neoplasia. Nature. 2003;421:639–43. 12571598 10.1038/nature01283

[85] Tsuji T, Kawada Y, Kai-Murozono M, Komatsu S, Han SA, Takeuchi K, et al. Regulation of melanoma cell migration and invasion by laminin-5 and alpha3beta1 integrin (VLA-3). Clin Exp Metastasis. 2002;19:127–34. 11964076 10.1023/a:1014573204062

[86] Stewart RL, O’connor KL. Clinical significance of the integrin α6β4 in human malignancies. Lab Invest. 2015;95:976–86. 26121317 10.1038/labinvest.2015.82 PMC4554527

[87] Longmate WM, Varney S, Power D, Miskin RP, Anderson KE, DeFreest L, et al. Integrin α3β1 on tumor keratinocytes is essential to maintain tumor growth and promotes a tumor-supportive keratinocyte secretome. J Invest Dermatol. 2021;141:142-151.e6. 32454065 10.1016/j.jid.2020.05.080 PMC7680721

[88] Sachs N, Secades P, van Hulst L, Kreft M, Song JY, Sonnenberg A. Loss of integrin α3 prevents skin tumor formation by promoting epidermal turnover and depletion of slow-cycling cells. Proc Natl Acad Sci U S A. 2012;109:21468–73. 23236172 10.1073/pnas.1204614110 PMC3535625

[89] Missan DS, Mitchell K, Subbaram S, DiPersio CM. Integrin α3β1 signaling through MEK/ERK determines alternative polyadenylation of the MMP-9 mRNA transcript in immortalized mouse keratinocytes. PLoS ONE. 2015;10: e0119539. 25751421 10.1371/journal.pone.0119539 PMC4353714

[90] Marinkovich MP. Laminin 332 in squamous-cell carcinoma. Nat Rev Cancer. 2007;7:370–80. 17457303 10.1038/nrc2089

[91] Chung H, Suh EK, Han IO, Oh ES. Keratinocyte-derived laminin-332 promotes adhesion and migration in melanocytes and melanoma. J Biol Chem. 2011;286:13438–47. 21349841 10.1074/jbc.M110.166751 PMC3075690

[92] Hamasaki H, Koga K, Aoki M, Hamasaki M, Koshikawa N, Seiki M, et al. Expression of laminin 5-γ2 chain in cutaneous squamous cell carcinoma and its role in tumour invasion. Br J Cancer. 2011;105:824–32. 21829200 10.1038/bjc.2011.283 PMC3171006

[93] Rousselle P, Scoazec JY. Laminin 332 in cancer: when the extracellular matrix turns signals from cell anchorage to cell movement. Semin Cancer Biol. 2020;62:149–65. 31639412 10.1016/j.semcancer.2019.09.026

[94] Natarajan E, Saeb M, Crum CP, Woo SB, McKee PH, Rheinwald JG. Co-expression of p16(INK4A) and laminin 5 gamma2 by microinvasive and superficial squamous cell carcinomas in vivo and by migrating wound and senescent keratinocytes in culture. Am J Pathol. 2003;163:477–91. 12875969 10.1016/s0002-9440(10)63677-2 PMC1868206

[95] Seftor RE, Seftor EA, Koshikawa N, Meltzer PS, Gardner LM, Bilban M, et al. Cooperative interactions of laminin 5 gamma2 chain, matrix metalloproteinase-2, and membrane type-1-matrix/metalloproteinase are required for mimicry of embryonic vasculogenesis by aggressive melanoma. Cancer Res. 2001;61:6322–7. 11522618

[96] Hamasaki H, Koga K, Hamasaki M, Kiryu H, Nakayama J, Iwasaki H, et al. Immunohistochemical analysis of laminin 5-γ2 chain expression for differentiation of basal cell carcinoma from trichoblastoma. Histopathology. 2011;59:159–61. 21771037 10.1111/j.1365-2559.2011.03871.x

[97] Yamamoto K, Miyazaki K, Higashi S. Cholesterol sulfate alters substrate preference of matrix metalloproteinase-7 and promotes degradations of pericellular laminin-332 and fibronectin. J Biol Chem. 2010;285:28862–73. 20605794 10.1074/jbc.M110.136994 PMC2937913

[98] Kivisaari AK, Kallajoki M, Mirtti T, McGrath JA, Bauer JW, Weber F, et al. Transformation-specific matrix metalloproteinases (MMP)-7 and MMP-13 are expressed by tumour cells in epidermolysis bullosa-associated squamous cell carcinomas. Br J Dermatol. 2008;158:778–85. 18284387 10.1111/j.1365-2133.2008.08466.x

[99] Pirilä E, Sharabi A, Salo T, Quaranta V, Tu H, Heljasvaara R, et al. Matrix metalloproteinases process the laminin-5 gamma 2-chain and regulate epithelial cell migration. Biochem Biophys Res Commun. 2003;303:1012–7. 12684035 10.1016/s0006-291x(03)00452-2

[100] Sadowski T, Dietrich S, Koschinsky F, Ludwig A, Proksch E, Titz B, et al. Matrix metalloproteinase 19 processes the laminin 5 gamma 2 chain and induces epithelial cell migration. Cell Mol Life Sci. 2005;62:870–80. 15868410 10.1007/s00018-005-4478-8 PMC11924473

[101] Tran M, Rousselle P, Nokelainen P, Tallapragada S, Nguyen NT, Fincher EF, et al. Targeting a tumor-specific laminin domain critical for human carcinogenesis. Cancer Res. 2008;68:2885–94. 18413757 10.1158/0008-5472.CAN-07-6160

[102] Litjens SH, de Pereda JM, Sonnenberg A. Current insights into the formation and breakdown of hemidesmosomes. Trends Cell Biol. 2006;16:376–83. 16757171 10.1016/j.tcb.2006.05.004

[103] Bachy S, Letourneur F, Rousselle P. Syndecan-1 interaction with the LG4/5 domain in laminin-332 is essential for keratinocyte migration. J Cell Physiol. 2008;214:238–49. 17579341 10.1002/jcp.21184

[104] Utani A, Momota Y, Endo H, Kasuya Y, Beck K, Suzuki N, et al. Laminin alpha 3 LG4 module induces matrix metalloproteinase-1 through mitogen-activated protein kinase signaling. J Biol Chem. 2003;278:34483–90. 12826666 10.1074/jbc.M304827200

[105] Momota Y, Suzuki N, Kasuya Y, Kobayashi T, Mizoguchi M, Yokoyama F, et al. Lamininα3 LG4 module induces keratinocyte migration: involvement of matrix metalloproteinase-9. J Recept Signal Transduct Res. 2005;25:1–17. 15960391 10.1081/rrs-200047870

[106] Katayama H. Mechanism of anchorage-independency and tumor formation of cancer cells: possible involvement of cell membrane-bound laminin-332. Cell Tissue Res. 2020;379:255–9. 31705213 10.1007/s00441-019-03114-7

[107] Owens DM, Watt FM. Contribution of stem cells and differentiated cells to epidermal tumours. Nat Rev Cancer. 2003;3:444–51. 12778134 10.1038/nrc1096

[108] Stamp GW, Pignatelli M. Distribution of beta 1, alpha 1, alpha 2 and alpha 3 integrin chains in basal cell carcinomas. J Pathol. 1991;163:307–13. 2033490 10.1002/path.1711630407

[109] Tennenbaum T, Weiner AK, Belanger AJ, Glick AB, Hennings H, Yuspa SH. The suprabasal expression of alpha 6 beta 4 integrin is associated with a high risk for malignant progression in mouse skin carcinogenesis. Cancer Res. 1993;53:4803–10. 8402665

[110] Owens DM, Watt FM. Influence of of beta1 integrins on epidermal squamous cell carcinoma formation in a transgenic mouse model: alpha3beta1, but not alpha2beta1, suppresses malignant conversion. Cancer Res. 2001;61:5248–54. 11431366

[111] Maalouf SW, Theivakumar S, Owens DM. Epidermal α6β4 integrin stimulates the influx of immunosuppressive cells during skin tumor promotion. J Dermatol Sci. 2012;66:108–18. 22464766 10.1016/j.jdermsci.2012.02.009 PMC3328604

[112] Peltonen J, Larjava H, Jaakkola S, Gralnick H, Akiyama SK, Yamada SS, et al. Localization of integrin receptors for fibronectin, collagen, and laminin in human skin. Variable expression in basal and squamous cell carcinomas*.* J Clin Invest. 1989; 84:1916–1923. 10.1172/JCI114379 PMC304072 2556449

[113] De Luca M, Pellegrini G, Zambruno G, Marchisio PC. Role of integrins in cell adhesion and polarity in normal keratinocytes and human skin pathologies. J Dermatol. 1994;21:821–8. 7852642 10.1111/j.1346-8138.1994.tb03296.x

[114] Owens DM, Romero MR, Gardner C, Watt FM. Suprabasal alpha6beta4 integrin expression in epidermis results in enhanced tumourigenesis and disruption of TGFbeta signalling. J Cell Sci. 2003;116:3783–91. 12902406 10.1242/jcs.00725

[115] Hobbs RM, Silva-Vargas V, Groves R, Watt FM. Expression of activated MEK1 in differentiating epidermal cells is sufficient to generate hyperproliferative and inflammatory skin lesions. J Invest Dermatol. 2004;123:503–15. 15304090 10.1111/j.0022-202X.2004.23225.x

[116] Haase I, Hobbs RM, Romero MR, Broad S, Watt FM. A role for mitogen-activated protein kinase activation by integrins in the pathogenesis of psoriasis. J Clin Invest. 2001;108:527–36. 11518726 10.1172/JCI12153 PMC209397

[117] Ramovs V, Krotenberg Garcia A, Kreft M, Sonnenberg A. Integrin α3β1 is a key regulator of several protumorigenic pathways during skin carcinogenesis. J Invest Dermatol. 2021;141:732-741.e6. 32805217 10.1016/j.jid.2020.07.024

[118] Picco ME, Castro MV, Quezada MJ, Barbero G, Villanueva MB, Fernández NB, et al. STAT3 enhances the constitutive activity of AGC kinases in melanoma by transactivating PDK1. Cell Biosci. 2019;9:3. 30622697 10.1186/s13578-018-0265-8 PMC6317239

[119] Vogt PK, Hart JR. PI3K and STAT3: a new alliance. Cancer Discov. 2011;1:481–6. 22348200 10.1158/2159-8290.CD-11-0218 PMC3279943

[120] Chan KS, Sano S, Kiguchi K, Anders J, Komazawa N, Takeda J, et al. Disruption of Stat3 reveals a critical role in both the initiation and the promotion stages of epithelial carcinogenesis. J Clin Invest. 2004;114:720–8. 15343391 10.1172/JCI21032 PMC514583

[121] Segrelles C, Lu J, Hammann B, Santos M, Moral M, Cascallana JL, et al. Deregulated activity of Akt in epithelial basal cells induces spontaneous tumors and heightened sensitivity to skin carcinogenesis. Cancer Res. 2007;67:10879–88. 18006833 10.1158/0008-5472.CAN-07-2564

[122] Ramovs V, Krotenberg Garcia A, Song JY, de Rink I, Kreft M, Goldschmeding R, et al. Integrin α3β1 in hair bulge stem cells modulates CCN2 expression and promotes skin tumorigenesis. Life Sci Alliance. 2020;3: e202000645. 32423907 10.26508/lsa.202000645 PMC7240742

[123] Wang H, Leavitt L, Ramaswamy R, Rapraeger AC. Interaction of syndecan and alpha6beta4 integrin cytoplasmic domains: regulation of ErbB2-mediated integrin activation. J Biol Chem. 2010;285:13569–21357. 20181947 10.1074/jbc.M110.102137 PMC2859518

[124] Mercurio AM, Rabinovitz I, Shaw LM. The α6β4 integrin and epithelial cell migration. Curr Opin Cell Biol. 2001;13:541–5. 11544021 10.1016/s0955-0674(00)00249-0

[125] Fisher ML, Keillor JW, Xu W, Eckert RL, Kerr C. Transglutaminase is required for epidermal squamous cell carcinoma stem cell survival. Mol Cancer Res. 2015;13:1083–94. 25934691 10.1158/1541-7786.MCR-14-0685-T PMC4504806

[126] Fisher ML, Kerr C, Adhikary G, Grun D, Xu W, Keillor JW, et al. Transglutaminase interaction with α6/β4-integrin stimulates YAP1-dependent ΔNp63α stabilization and leads to enhanced cancer stem cell survival and tumor formation. Cancer Res. 2016;76:7265–76. 27780825 10.1158/0008-5472.CAN-16-2032 PMC5161627

[127] Kim NG, Gumbiner BM. Adhesion to fibronectin regulates Hippo signaling via the FAK-Src-PI3K pathway. J Cell Biol. 2015;210:503–15. 26216901 10.1083/jcb.201501025 PMC4523609

[128] Choi H-R, Nam K-M, Park S-J, Kim D-S, Huh C-H, Park W-Y, et al. Suppression of miR135b increases the proliferative potential of normal human keratinocytes. J Invest Dermatol. 2014;134:1161. 24129066 10.1038/jid.2013.427

[129] Pellegrini G, Dellambra E, Golisano O, Martinelli E, Fantozzi I, Bondanza S, et al. p63 identifies keratinocyte stem cells. Proc Natl Acad Sci U S A. 2001;98:3156–61. 11248048 10.1073/pnas.061032098 PMC30623

[130] Hasche D, Stephan S, Braspenning-Wesch I, Mikulec J, Niebler M, Gröne HJ, et al. The interplay of UV and cutaneous papillomavirus infection in skin cancer development. PLoS Pathog. 2017;13: e1006723. 29190285 10.1371/journal.ppat.1006723 PMC5708609

[131] Bassi DE, Lopez De Cicco R, Cenna J, Litwin S, Cukierman E, and Klein-Szanto AJP. PACE4 expression in mouse basal keratinocytes results in basement membrane disruption and acceleration of tumor progression. Cancer Res. 2005; 65:7310–7319. 10.1158/0008-5472.CAN-05-1213 16103082

[132] Schmoeckel C, Stolz W, Sakai LY, Burgeson RE, Timpl R, Krieg T. Structure of basement membranes in malignant melanoma and nevocytic nevi. J Invest Dermatol. 1989;92:663–8. 2497191 10.1111/1523-1747.ep12696845

[133] Hu Y, Wang Q, Zhu XH. MiR-135b is a novel oncogenic factor in cutaneous melanoma by targeting LATS2. Melanoma Res. 2019;29:119–25. 30480622 10.1097/CMR.0000000000000524

[134] Olasz EB, Seline LN, Schock AM, Duncan NE, Lopez A, Lazar J, et al. MicroRNA-135b regulates leucine zipper tumor suppressor 1 in cutaneous squamous cell carcinoma. PLoS ONE. 2015;10: e0125412. 25938461 10.1371/journal.pone.0125412 PMC4418692

[135] Xu J, Rodriguez D, Petitclerc E, Kim JJ, Hangai M, Moon YS, et al. Proteolytic exposure of a cryptic site within collagen type IV is required for angiogenesis and tumor growth in vivo. J Cell Biol. 2001;154:1069–79. 11535623 10.1083/jcb.200103111 PMC2196184

[136] Maeshima Y, Colorado PC, Kalluri R. Two RGD-independent alpha vbeta 3 integrin binding sites on tumstatin regulate distinct anti-tumor properties. J Biol Chem. 2000;275:23745–50. 10837460 10.1074/jbc.C000186200

[137] Maeshima Y, Colorado PC, Torre A, Holthaus KA, Grunkemeyer JA, Ericksen MB, et al. Distinct antitumor properties of a type IV collagen domain derived from basement membrane. J Biol Chem. 2000;275:21340–8. 10766752 10.1074/jbc.M001956200

[138] Han J, Ohno N, Pasco S, Monboisse JC, Borel JP, Kefalides NA. A cell binding domain from the alpha3 chain of type IV collagen inhibits proliferation of melanoma cells. J Biol Chem. 1997;272:20395–401. 9252346 10.1074/jbc.272.33.20395

[139] Rübsam M, Broussard JA, Wickström SA, Nekrasova O, Green KJ, Niessen CM. Adherens junctions and desmosomes coordinate mechanics and signaling to orchestrate tissue morphogenesis and function: an evolutionary perspective. Cold Spring Harb Perspect Biol. 2018;10: a029207. 28893859 10.1101/cshperspect.a029207 PMC6211388

[140] Lay K, Kume T, Fuchs E. FOXC1 maintains the hair follicle stem cell niche and governs stem cell quiescence to preserve long-term tissue-regenerating potential. Proc Natl Acad Sci U S A. 2016;113:E1506–15. 26912458 10.1073/pnas.1601569113 PMC4801248

[141] Yao M, Qiu W, Liu R, Efremov AK, Cong P, Seddiki R, et al. Force-dependent conformational switch of α-catenin controls vinculin binding. Nat Commun. 2014;5:4525. 25077739 10.1038/ncomms5525

[142] Heuberger J, Birchmeier W. Interplay of cadherin-mediated cell adhesion and canonical Wnt signaling. Cold Spring Harb Perspect Biol. 2010;2: a002915. 20182623 10.1101/cshperspect.a002915 PMC2828280

[143] Davis MA, Ireton RC, Reynolds AB. A core function for p120-catenin in cadherin turnover. J Cell Biol. 2003;163:525–34. 14610055 10.1083/jcb.200307111 PMC2173649

[144] Wildenberg GA, Dohn MR, Carnahan RH, Davis MA, Lobdell NA, Settleman J, et al. p120-catenin and p190RhoGAP regulate cell-cell adhesion by coordinating antagonism between Rac and Rho. Cell. 2006;127:1027–39. 17129786 10.1016/j.cell.2006.09.046

[145] Anastasiadis PZ, Moon SY, Thoreson MA, Mariner DJ, Crawford HC, Zheng Y, et al. Inhibition of RhoA by p120 catenin. Nat Cell Biol. 2000;2:637–44. 10980705 10.1038/35023588

[146] Miroshnikova YA, Le HQ, Schneider D, Thalheim T, Rübsam M, Bremicker N, et al. Adhesion forces and cortical tension couple cell proliferation and differentiation to drive epidermal stratification. Nat Cell Biol. 2018;20:69–80. 29230016 10.1038/s41556-017-0005-z

[147] Simpson CL, Patel DM, Green KJ. Deconstructing the skin: cytoarchitectural determinants of epidermal morphogenesis. Nat Rev Mol Cell Biol. 2011;12:565–80. 21860392 10.1038/nrm3175 PMC3280198

[148] Braga VM, Betson M, Li X, Lamarche-Vane N. Activation of the small GTPase Rac is sufficient to disrupt cadherin-dependent cell-cell adhesion in normal human keratinocytes. Mol Biol Cell. 2000;11:3703–21. 11071901 10.1091/mbc.11.11.3703 PMC15031

[149] Akhtar N, Hotchin NA. RAC1 regulates adherens junctions through endocytosis of E-cadherin. Mol Biol Cell. 2001;12:847–62. 11294891 10.1091/mbc.12.4.847 PMC32271

[150] Benham-Pyle BW, Pruitt BL, and Nelson WJ. Cell adhesion. Mechanical strain induces E-cadherin-dependent Yap1 and β-catenin activation to drive cell cycle entry*.* Science. 2015; 348:1024–1027. 10.1126/science.aaa4559 PMC4572847 26023140

[151] Hirata H, Samsonov M, Sokabe M. Actomyosin contractility provokes contact inhibition in E-cadherin-ligated keratinocytes. Sci Rep. 2017;7:46326. 28406163 10.1038/srep46326 PMC5390311

[152] Silvis MR, Kreger BT, Lien WH, Klezovitch O, Rudakova GM, Camargo FD, et al. α-catenin is a tumor suppressor that controls cell accumulation by regulating the localization and activity of the transcriptional coactivator Yap1*.* Sci Signal. 2011; 4:ra33. 10.1126/scisignal.2001823 PMC3366274 21610251

[153] Schlegelmilch K, Mohseni M, Kirak O, Pruszak J, Rodriguez JR, Zhou D, et al. Yap1 acts downstream of α-catenin to control epidermal proliferation. Cell. 2011;144:782–95. 21376238 10.1016/j.cell.2011.02.031 PMC3237196

[154] Li P, Silvis MR, Honaker Y, Lien WH, Arron ST, Vasioukhin V. αE-catenin inhibits a Src-YAP1 oncogenic module that couples tyrosine kinases and the effector of Hippo signaling pathway. Genes Dev. 2016;30:798–811. 27013234 10.1101/gad.274951.115 PMC4826396

[155] Kobielak A, Fuchs E. Links between alpha-catenin, NF-kappaB, and squamous cell carcinoma in skin. Proc Natl Acad Sci U S A. 2006;103:2322–7. 16452166 10.1073/pnas.0510422103 PMC1413714

[156] Papanikolaou S, Bravou V, Gyftopoulos K, Nakas D, Repanti M, Papadaki H. ILK expression in human basal cell carcinoma correlates with epithelial-mesenchymal transition markers and tumour invasion. Histopathology. 2010;56:799–809. 20546345 10.1111/j.1365-2559.2010.03556.x

[157] Tucci MG, Lucarini G, Brancorsini D, Zizzi A, Pugnaloni A, Giacchetti A, et al. Involvement of E-cadherin, beta-catenin, Cdc42 and CXCR4 in the progression and prognosis of cutaneous melanoma. Br J Dermatol. 2007;157:1212–6. 17970806 10.1111/j.1365-2133.2007.08246.x

[158] Kreizenbeck GM, Berger AJ, Subtil A, Rimm DL, Gould Rothberg BE. Prognostic significance of cadherin-based adhesion molecules in cutaneous malignant melanoma. Cancer Epidemiol Biomarkers Prev. 2008;17:949–58. 18398036 10.1158/1055-9965.EPI-07-2729 PMC3312613

[159] Bosch FX, Andl C, Abel U, Kartenbeck J. E-cadherin is a selective and strongly dominant prognostic factor in squamous cell carcinoma: a comparison of E-cadherin with desmosomal components. Int J Cancer. 2005;114:779–90. 15609307 10.1002/ijc.20782

[160] Livshits G, Kobielak A, Fuchs E. Governing epidermal homeostasis by coupling cell–cell adhesion to integrin and growth factor signaling, proliferation, and apoptosis. Proc Natl Acad Sci U S A. 2012;109:4886–91. 22411810 10.1073/pnas.1202120109 PMC3324018

[161] Qi J, Wang J, Romanyuk O, Siu CH. Involvement of Src family kinases in N-cadherin phosphorylation and beta-catenin dissociation during transendothelial migration of melanoma cells. Mol Biol Cell. 2006;17:1261–72. 16371504 10.1091/mbc.E05-10-0927 PMC1382315

[162] Kovacs D, Migliano E, Muscardin L, Silipo V, Catricalà C, Picardo M, et al. The role of Wnt/β-catenin signaling pathway in melanoma epithelial-to-mesenchymal-like switching: evidences from patients-derived cell lines. Oncotarget. 2016;7:43295–314. 27175588 10.18632/oncotarget.9232 PMC5190024

[163] Delgado-Bellido D, Zamudio-Martínez E, Fernández-Cortés M, Herrera-Campos AB, Olmedo-Pelayo J, Perez CJ, et al. VE-cadherin modulates β-catenin/TCF-4 to enhance vasculogenic mimicry. Cell Death Dis. 2023;14:135. 36797281 10.1038/s41419-023-05666-7 PMC9935922

[164] Perez-Moreno M, Song W, Pasolli HA, Williams SE, Fuchs E. Loss of p120 catenin and links to mitotic alterations, inflammation, and skin cancer. Proc Natl Acad Sci U S A. 2008;105:15399–404. 18809907 10.1073/pnas.0807301105 PMC2547465

[165] Méant A, Gao B, Lavoie G, Nourreddine S, Jung F, Aubert L, et al. Proteomic analysis reveals a role for RSK in p120-catenin phosphorylation and melanoma cell-cell adhesion. Mol Cell Proteomics. 2020;19:50–64. 31678930 10.1074/mcp.RA119.001811 PMC6944238

[166] Yanagisawa M, Anastasiadis PZ. p120 catenin is essential for mesenchymal cadherin-mediated regulation of cell motility and invasiveness. J Cell Biol. 2006;174:1087–96. 16982802 10.1083/jcb.200605022 PMC2064398

[167] Bozdogan O, Vargel I, Cavusoglu T, Karabulut AA, Karahan G, Sayar N, et al. Metastasis suppressor proteins in cutaneous squamous cell carcinoma. Pathol Res Pract. 2016;212:608–15. 27215390 10.1016/j.prp.2015.12.018

[168] Majima Y, Hirakawa S, Kito Y, Suzuki H, Koide M, Fukamizu H, et al. Twist1 as a possible biomarker for metastatic basal cell carcinoma. Acta Derm Venereol. 2012;92:621–2. 22965800 10.2340/00015555-1422

[169] Kuphal S, Bosserhoff AK. E-cadherin cell-cell communication in melanogenesis and during development of malignant melanoma. Arch Biochem Biophys. 2012;524:43–7. 22085498 10.1016/j.abb.2011.10.020

[170] Spangler B, Kappelmann M, Schittek B, Meierjohann S, Vardimon L, Bosserhoff AK, et al. ETS-1/RhoC signaling regulates the transcription factor c-Jun in melanoma. Int J Cancer. 2012;130:2801–11. 21732343 10.1002/ijc.26277

[171] Hong IK, Jin YJ, Byun HJ, Jeoung DI, Kim YM, Lee H. Homophilic interactions of Tetraspanin CD151 up-regulate motility and matrix metalloproteinase-9 expression of human melanoma cells through adhesion-dependent c-Jun activation signaling pathways. J Biol Chem. 2006;281:24279–92. 16798740 10.1074/jbc.M601209200

[172] Zhang Y, Li X, Li J, Hu H, Miao X, Song X, et al. Human hemokinin-1 promotes migration of melanoma cells and increases MMP-2 and MT1-MMP expression by activating tumor cell NK1 receptors. Peptides. 2016;83:8–15. 27458061 10.1016/j.peptides.2016.07.004

[173] Hsu MY, Meier FE, Nesbit M, Hsu JY, Van Belle P, Elder DE, et al. E-cadherin expression in melanoma cells restores keratinocyte-mediated growth control and down-regulates expression of invasion-related adhesion receptors. Am J Pathol. 2000;156:1515–25. 10793063 10.1016/S0002-9440(10)65023-7 PMC1876923

[174] Nguyen T, Mège RM. N-cadherin and fibroblast growth factor receptors crosstalk in the control of developmental and cancer cell migrations. Eur J Cell Biol. 2016;95:415–26. 27320194 10.1016/j.ejcb.2016.05.002

[175] Li G, Satyamoorthy K, Herlyn M. N-cadherin-mediated intercellular interactions promote survival and migration of melanoma cells. Cancer Res. 2001;61:3819–25. 11325858

[176] Hao L, Ha JR, Kuzel P, Garcia E, Persad S. Cadherin switch from E- to N-cadherin in melanoma progression is regulated by the PI3K/PTEN pathway through Twist and Snail. Br J Dermatol. 2012;166:1184–97. 22332917 10.1111/j.1365-2133.2012.10824.x

[177] Takahara M, Chen S, Kido M, Takeuchi S, Uchi H, Tu Y, et al. Stromal CD10 expression, as well as increased dermal macrophages and decreased Langerhans cells, are associated with malignant transformation of keratinocytes. J Cutan Pathol. 2009;36:668–74. 19515046 10.1111/j.1600-0560.2008.01139.x

[178] Sasaki K, Sugai T, Ishida K, Osakabe M, Amano H, Kimura H, et al. Analysis of cancer-associated fibroblasts and the epithelial-mesenchymal transition in cutaneous basal cell carcinoma, squamous cell carcinoma, and malignant melanoma. Hum Pathol. 2018;79:1–8. 29555579 10.1016/j.humpath.2018.03.006

[179] Murtas D, Maxia C, Diana A, Pilloni L, Corda C, Minerba L, et al. Role of epithelial-mesenchymal transition involved molecules in the progression of cutaneous melanoma. Histochem Cell Biol. 2017;148:639–49. 28828681 10.1007/s00418-017-1606-0

[180] D’Arcy C, Kiel C. Cell adhesion molecules in normal skin and melanoma. Biomolecules. 2021;11:1213. 34439879 10.3390/biom11081213 PMC8391223

[181] Barrette K, Van Kelst S, Wouters J, Marasigan V, Fieuws S, Agostinis P, et al. Epithelial-mesenchymal transition during invasion of cutaneous squamous cell carcinoma is paralleled by AKT activation. Br J Dermatol. 2014;171:1014–21. 24628329 10.1111/bjd.12967

[182] Cao H-H, Chu J-H, Kwan H-Y, Su T, Yu H, Cheng C-Y, et al. Inhibition of the STAT3 signaling pathway contributes to apigenin-mediated anti-metastatic effect in melanoma. Sci Rep. 2016;6:21731. 26911838 10.1038/srep21731 PMC4766576

[183] Tsai JH, Donaher JL, Murphy DA, Chau S, Yang J. Spatiotemporal regulation of epithelial-mesenchymal transition is essential for squamous cell carcinoma metastasis. Cancer Cell. 2012;22:725–36. 23201165 10.1016/j.ccr.2012.09.022 PMC3522773

[184] Yan S, Holderness BM, Li Z, Seidel GD, Gui J, Fisher JL, et al. Epithelial-mesenchymal expression phenotype of primary melanoma and matched metastases and relationship with overall survival. Anticancer Res. 2016;36:6449–56. 27919967 10.21873/anticanres.11243 PMC5576452

[185] Wels C, Joshi S, Koefinger P, Bergler H, Schaider H. Transcriptional activation of ZEB1 by Slug leads to cooperative regulation of the epithelial-mesenchymal transition-like phenotype in melanoma. J Invest Dermatol. 2011;131:1877–85. 21593765 10.1038/jid.2011.142 PMC3182526

[186] Moreno-Bueno G, Cubillo E, Sarrió D, Peinado Hc, Rodríguez-Pinilla SMa, Villa S, et al. Genetic profiling of epithelial cells expressing E-cadherin repressors reveals a distinct role for Snail, Slug, and E47 factors in epithelial-mesenchymal transition. Cancer Res. 2006; 66:9543–9556. 10.1158/0008-5472.CAN-06-0479 17018611

[187] Massoumi R, Kuphal S, Hellerbrand C, Haas B, Wild P, Spruss T, et al. Down-regulation of CYLD expression by Snail promotes tumor progression in malignant melanoma. J Exp Med. 2009;206:221–32. 19124656 10.1084/jem.20082044 PMC2626666

[188] Yang J, Mani SA, Donaher JL, Ramaswamy S, Itzykson RA, Come C, et al. Twist, a master regulator of morphogenesis, plays an essential role in tumor metastasis. Cell. 2004;117:927–39. 15210113 10.1016/j.cell.2004.06.006

[189] Liu Z-J, Xiao M, Balint K, Smalley KSM, Brafford P, Qiu R, et al. Notch1 signaling promotes primary melanoma progression by activating mitogen-activated protein kinase/phosphatidylinositol 3-kinase-Akt pathways and up-regulating N-cadherin expression. Cancer Res. 2006;66:4182–90. 16618740 10.1158/0008-5472.CAN-05-3589

[190] Alexaki VI, Javelaud D, Van Kempen LC, Mohammad KS, Dennler S, Luciani F, et al. GLI2-mediated melanoma invasion and metastasis. J Natl Cancer Inst. 2010;102:1148–59. 20660365 10.1093/jnci/djq257 PMC2914763

[191] Kuphal S, Poser I, Jobin C, Hellerbrand C, Bosserhoff AK. Loss of E-cadherin leads to upregulation of NFκB activity in malignant melanoma. Oncogene. 2004;23:8509–19. 15378016 10.1038/sj.onc.1207831

[192] Canel M, Serrels A, Miller D, Timpson P, Serrels B, Frame MC, et al. Quantitative in vivo imaging of the effects of inhibiting integrin signaling via Src and FAK on cancer cell movement: effects on E-cadherin dynamics. Cancer Res. 2010;70:9413–22. 21045155 10.1158/0008-5472.CAN-10-1454 PMC3079905

[193] Serrels A, Canel M, Brunton VG, Frame MC. Src/FAK-mediated regulation of E-cadherin as a mechanism for controlling collective cell movement: insights from in vivo imaging. Cell Adh Migr. 2011;5:360–5. 21836391 10.4161/cam.5.4.17290 PMC3210304

[194] Higgins CA, Roger MF, Hill RP, Ali-Khan AS, Garlick JA, Christiano AM, et al. Multifaceted role of hair follicle dermal cells in bioengineered skins. Br J Dermatol. 2017;176:1259–69. 27679975 10.1111/bjd.15087

[195] Hiraoka C, Toki F, Shiraishi K, Sayama K, Nishimura EK, Miura H, et al. Two clonal types of human skin fibroblasts with different potentials for proliferation and tissue remodeling ability. J Dermatol Sci. 2016;82:84–94. 26867959 10.1016/j.jdermsci.2016.01.009

[196] Driskell RR, Lichtenberger BM, Hoste E, Kretzschmar K, Simons BD, Charalambous M, et al. Distinct fibroblast lineages determine dermal architecture in skin development and repair. Nature. 2013;504:277–81. 24336287 10.1038/nature12783 PMC3868929

[197] Lee DY, Cho KH. The effects of epidermal keratinocytes and dermal fibroblasts on the formation of cutaneous basement membrane in three-dimensional culture systems. Arch Dermatol Res. 2005;296:296–302. 15650892 10.1007/s00403-004-0529-5

[198] Lichtenberger BM, Mastrogiannaki M, Watt FM. Epidermal β-catenin activation remodels the dermis via paracrine signalling to distinct fibroblast lineages. Nat Commun. 2016;7:10537. 26837596 10.1038/ncomms10537 PMC4742837

[199] Mastrogiannaki M, Lichtenberger BM, Reimer A, Collins CA, Driskell RR, Watt FM. β-Catenin stabilization in skin fibroblasts causes fibrotic lesions by preventing adipocyte differentiation of the reticular dermis. J Invest Dermatol. 2016;136:1130–42. 26902921 10.1016/j.jid.2016.01.036 PMC4874948

[200] Ito M, Yang Z, Andl T, Cui C, Kim N, Millar SE, et al. Wnt-dependent de novo hair follicle regeneration in adult mouse skin after wounding. Nature. 2007;447:316–20. 17507982 10.1038/nature05766

[201] Collins CA, Kretzschmar K, Watt FM. Reprogramming adult dermis to a neonatal state through epidermal activation of β-catenin. Development. 2011;138:5189–99. 22031549 10.1242/dev.064592 PMC3210498

[202] Daszczuk P, Mazurek P, Pieczonka TD, Olczak A, Boryń ŁM, Kobielak K. An intrinsic oscillation of gene networks inside hair follicle stem cells: an additional layer that can modulate hair stem cell activities. Front Cell Dev Biol. 2020;8: 595178. 33363148 10.3389/fcell.2020.595178 PMC7758224

[203] Mazurkiewicz J, Simiczyjew A, Dratkiewicz E, Pietraszek-Gremplewicz K, Majkowski M, Kot M, et al. Melanoma cells with diverse invasive potential differentially induce the activation of normal human fibroblasts. Cell Commun Signal. 2022;20:63. 35538545 10.1186/s12964-022-00871-x PMC9092709

[204] Schütz S, Solé-Boldo L, Lucena-Porcel C, Hoffmann J, Brobeil A, Lonsdorf AS, et al. Functionally distinct cancer-associated fibroblast subpopulations establish a tumor promoting environment in squamous cell carcinoma. Nat Commun. 2023;14:5413. 37669956 10.1038/s41467-023-41141-9 PMC10480447

[205] Omland SH, Wettergren EE, Mollerup S, Asplund M, Mourier T, Hansen AJ, et al. Cancer associated fibroblasts (CAFs) are activated in cutaneous basal cell carcinoma and in the peritumoural skin. BMC Cancer. 2017;17:675. 28987144 10.1186/s12885-017-3663-0 PMC5806272

[206] Erdogan B, Webb DJ. Cancer-associated fibroblasts modulate growth factor signaling and extracellular matrix remodeling to regulate tumor metastasis. Biochem Soc Trans. 2017;45:229–36. 28202677 10.1042/BST20160387 PMC5371349

[207] Xiao Y, Zhou L, Andl T, Zhang Y. YAP1 controls the N-cadherin-mediated tumor-stroma interaction in melanoma progression. Oncogene. 2024;43:884–98. 38308096 10.1038/s41388-024-02953-1 PMC10942861

[208] Lee JT, Herlyn M. Microenvironmental influences in melanoma progression. J Cell Biochem. 2007;101:862–72. 17171636 10.1002/jcb.21204

[209] Zhou Q, Jin X, Zhao Y, Wang Y, Tao M, Cao Y, et al. Melanoma-associated fibroblasts in tumor-promotion flammation and antitumor immunity: novel mechanisms and potential immunotherapeutic strategies. Hum Mol Genet. 2024;33:1186–93. 38538564 10.1093/hmg/ddae056 PMC11190611

[210] Ohshio Y, Teramoto K, Hanaoka J, Tezuka N, Itoh Y, Asai T, et al. Cancer-associated fibroblast-targeted strategy enhances antitumor immune responses in dendritic cell-based vaccine. Cancer Sci. 2015;106:134–42. 25483888 10.1111/cas.12584 PMC4399032

[211] Khalili JS, Liu S, Rodríguez-Cruz TG, Whittington M, Wardell S, Liu C, et al. Oncogenic BRAF(V600E) promotes stromal cell-mediated immunosuppression via induction of interleukin-1 in melanoma. Clin Cancer Res. 2012;18:5329–40. 22850568 10.1158/1078-0432.CCR-12-1632 PMC3463754

[212] Ziani L, Buart S, Chouaib S, Thiery J. Hypoxia increases melanoma-associated fibroblasts immunosuppressive potential and inhibitory effect on T cell-mediated cytotoxicity. Oncoimmunology. 2021;10:1950953. 34367731 10.1080/2162402X.2021.1950953 PMC8312612

[213] Ziani L, Safta-Saadoun TB, Gourbeix J, Cavalcanti A, Robert C, Favre G, et al. Melanoma-associated fibroblasts decrease tumor cell susceptibility to NK cell-mediated killing through matrix-metalloproteinases secretion. Oncotarget. 2017;8:19780–94. 28423623 10.18632/oncotarget.15540 PMC5386721

[214] Balsamo M, Scordamaglia F, Pietra G, Manzini C, Cantoni C, Boitano M, et al. Melanoma-associated fibroblasts modulate NK cell phenotype and antitumor cytotoxicity. Proc Natl Acad Sci U S A. 2009;106:20847–52. 19934056 10.1073/pnas.0906481106 PMC2791633

[215] Lakins MA, Ghorani E, Munir H, Martins CP, Shields JD. Cancer-associated fibroblasts induce antigen-specific deletion of CD8+T Cells to protect tumour cells. Nat Commun. 2018;9:948. 29507342 10.1038/s41467-018-03347-0 PMC5838096

[216] Lee JH, Shklovskaya E, Lim SY, Carlino MS, Menzies AM, Stewart A, et al. Transcriptional downregulation of MHC class I and melanoma de-differentiation in resistance to PD-1 inhibition. Nat Commun. 2020;11:1897. 32312968 10.1038/s41467-020-15726-7 PMC7171183

[217] Zhou L, Yang K, Andl T, Wickett RR, Zhang Y. Perspective of targeting cancer-associated fibroblasts in melanoma. J Cancer. 2015;6:717–26. 26185533 10.7150/jca.10865 PMC4504107

[218] Érsek B, Silló P, Cakir U, Molnár V, Bencsik A, Mayer B, et al. Melanoma-associated fibroblasts impair CD8+ T cell function and modify expression of immune checkpoint regulators via increased arginase activity. Cell Mol Life Sci. 2021;78:661–73. 32328671 10.1007/s00018-020-03517-8 PMC7581550

[219] Wong PF, Wei W, Gupta S, Smithy JW, Zelterman D, Kluger HM, et al. Multiplex quantitative analysis of cancer-associated fibroblasts and immunotherapy outcome in metastatic melanoma. J Immunother Cancer. 2019;7:194. 31337426 10.1186/s40425-019-0675-0 PMC6651990

[220] Zhao F, Evans K, Xiao C, DeVito N, Theivanthiran B, Holtzhausen A, et al. Stromal fibroblasts mediate anti-PD-1 resistance via MMP-9 and dictate TGFβ inhibitor sequencing in melanoma. Cancer Immunol Res. 2018;6:1459–71. 30209062 10.1158/2326-6066.CIR-18-0086 PMC6279598

[221] Li X, Zhao S, Bian X, Zhang L, Lu L, Pei S, et al. Signatures of EMT, immunosuppression, and inflammation in primary and recurrent human cutaneous squamous cell carcinoma at single-cell resolution. Theranostics. 2022;12:7532–49. 36438481 10.7150/thno.77528 PMC9691356

[222] Tsang M, Quesnel K, Vincent K, Hutchenreuther J, Postovit LM, Leask A. Insights into fibroblast plasticity: cellular communication network 2 is required for activation of cancer-associated fibroblasts in a murine model of melanoma. Am J Pathol. 2020;190:206–21. 31610176 10.1016/j.ajpath.2019.09.006

[223] Novotný J, Strnadová K, Dvořánková B, Kocourková Š, Jakša R, Dundr P, et al. Single-cell RNA sequencing unravels heterogeneity of the stromal niche in cutaneous melanoma heterogeneous spheroids. Cancers (Basel). 2020;12:3324. 33182777 10.3390/cancers12113324 PMC7697260

[224] Lederle W, Hartenstein B, Meides A, Kunzelmann H, Werb Z, Angel P, et al. MMP13 as a stromal mediator in controlling persistent angiogenesis in skin carcinoma. Carcinogenesis. 2010;31:1175–84. 19892798 10.1093/carcin/bgp248 PMC2893794

[225] Demkova L, Kucerova L. Role of the HGF/c-MET tyrosine kinase inhibitors in metastasic melanoma. Mol Cancer. 2018;17:26. 29455657 10.1186/s12943-018-0795-z PMC5817811

[226] Kinugasa Y, Matsui T, Takakura N. CD44 expressed on cancer-associated fibroblasts is a functional molecule supporting the stemness and drug resistance of malignant cancer cells in the tumor microenvironment. Stem Cells. 2014;32:145–56. 24395741 10.1002/stem.1556

[227] Kumamoto T, Shalhevet D, Matsue H, Mummert ME, Ward BR, Jester JV, et al. Hair follicles serve as local reservoirs of skin mast cell precursors. Blood. 2003;102:1654–60. 12738661 10.1182/blood-2003-02-0449

[228] Merad M, Sathe P, Helft J, Miller J, Mortha A. The dendritic cell lineage: ontogeny and function of dendritic cells and their subsets in the steady state and the inflamed setting. Annu Rev Immunol. 2013;31:563–604. 23516985 10.1146/annurev-immunol-020711-074950 PMC3853342

[229] Lee P, Gund R, Dutta A, Pincha N, Rana I, Ghosh S, et al. Stimulation of hair follicle stem cell proliferation through an IL-1 dependent activation of γδT-cells. Elife. 2017;6: e28875. 29199946 10.7554/eLife.28875 PMC5714500

[230] Castellana D, Paus R, Perez-Moreno M. Macrophages contribute to the cyclic activation of adult hair follicle stem cells. PLoS Biol. 2014;12: e1002002. 25536657 10.1371/journal.pbio.1002002 PMC4275176

[231] Ali N, Zirak B, Rodriguez RS, Pauli ML, Truong HA, Lai K, et al. Regulatory T cells in skin facilitate epithelial stem cell differentiation. Cell. 2017;169:1119-1129.e11. 28552347 10.1016/j.cell.2017.05.002 PMC5504703

[232] Rahmani W, Sinha S, Biernaskie J. Immune modulation of hair follicle regeneration. NPJ Regen Med. 2020;5:9. 32411394 10.1038/s41536-020-0095-2 PMC7214459

[233] Sanchez Rodriguez R, Pauli ML, Neuhaus IM, Yu SS, Arron ST, Harris HW, et al. Memory regulatory T cells reside in human skin. J Clin Invest. 2014;124:1027–36. 24509084 10.1172/JCI72932 PMC3934172

[234] Lui PP, Xu JZ, Aziz H, Sen M, Ali N. Jagged-1+ skin Tregs modulate cutaneous wound healing. Sci Rep. 2024;14:20999. 39251686 10.1038/s41598-024-71512-1 PMC11385218

[235] Nosbaum A, Prevel N, Truong HA, Mehta P, Ettinger M, Scharschmidt TC, et al. Cutting edge: regulatory T cells facilitate cutaneous wound healing. J Immunol. 2016;196:2010–4. 26826250 10.4049/jimmunol.1502139 PMC4761457

[236] Agudo J, Park ES, Rose SA, Alibo E, Sweeney R, Dhainaut M, et al. Quiescent tissue stem cells evade immune surveillance. Immunity. 2018;48:271-285.e5. 29466757 10.1016/j.immuni.2018.02.001 PMC5824652

[237] Mani V, Bromley SK, Äijö T, Mora-Buch R, Carrizosa E, Warner RD, et al. Migratory DCs activate TGF-β to precondition naïve CD8(+) T cells for tissue-resident memory fate*.* Science. 2019; 366:eaav5728. 10.1126/science.aav5728 PMC6939608 31601741

[238] Park SL, Buzzai A, Rautela J, Hor JL, Hochheiser K, Effern M, et al. Tissue-resident memory CD8+ T cells promote melanoma–immune equilibrium in skin. Nature. 2019;565:366–71. 30598548 10.1038/s41586-018-0812-9

[239] Rosenblum MD, Olasz EB, Yancey KB, Woodliff JE, Lazarova Z, Gerber KA, et al. Expression of CD200 on epithelial cells of the murine hair follicle: a role in tissue-specific immune tolerance? J Invest Dermatol. 2004;123:880–7. 15482475 10.1111/j.0022-202X.2004.23461.x

[240] Wang ECE, Dai Z, Ferrante AW, Drake CG, Christiano AM. A subset of TREM2(+) dermal macrophages secretes Oncostatin M to maintain hair follicle stem cell quiescence and inhibit hair growth. Cell Stem Cell. 2019;24:654-669.e6. 30930146 10.1016/j.stem.2019.01.011

[241] Passarelli A, Mannavola F, Stucci LS, Tucci M, Silvestris F. Immune system and melanoma biology: a balance between immunosurveillance and immune escape. Oncotarget. 2017;8:106132–42. 29285320 10.18632/oncotarget.22190 PMC5739707

[242] Benboubker V, Boivin F, Dalle S, Caramel J. Cancer cell phenotype plasticity as a driver of immune escape in melanoma. Front Immunol. 2022;13: 873116. 35432344 10.3389/fimmu.2022.873116 PMC9012258

[243] Habib S, Osborn G, Willsmore Z, Chew MW, Jakubow S, Fitzpatrick A, et al. Tumor associated macrophages as key contributors and targets in current and future therapies for melanoma. Expert Rev Clin Immunol. 2024;20:895–911. 38533720 10.1080/1744666X.2024.2326626 PMC11286214

[244] Saeidi V, Doudican N, Carucci JA. Understanding the squamous cell carcinoma immune microenvironment. Front Immunol. 2023;14:1084873. 36793738 10.3389/fimmu.2023.1084873 PMC9922717

[245] Wang X, Wan Q, Jin L, Liu C, Liu C, Cheng Y, et al. The integrative analysis identifies three cancer subtypes and stemness features in cutaneous melanoma. Front Mol Biosci. 2020;7: 598725. 33665205 10.3389/fmolb.2020.598725 PMC7921163

[246] Moussai D, Mitsui H, Pettersen JS, Pierson KC, Shah KR, Suárez-Farinas M, et al. The human cutaneous squamous cell carcinoma microenvironment is characterized by increased lymphatic density and enhanced expression of macrophage-derived VEGF-C. J Invest Dermatol. 2011;131:229–36. 20827282 10.1038/jid.2010.266

[247] Migden MR, Rischin D, Schmults CD, Guminski A, Hauschild A, Lewis KD, et al. PD-1 blockade with cemiplimab in advanced cutaneous squamous-cell carcinoma. N Engl J Med. 2018;379:341–51. 29863979 10.1056/NEJMoa1805131

[248] Chen DS, Mellman I. Oncology meets immunology: the cancer-immunity cycle. Immunity. 2013;39:1–10. 23890059 10.1016/j.immuni.2013.07.012

[249] Schatton T, Schütte U, Frank NY, Zhan Q, Hoerning A, Robles SC, et al. Modulation of T-cell activation by malignant melanoma initiating cells. Cancer Res. 2010;70:697–708. 20068175 10.1158/0008-5472.CAN-09-1592 PMC2883769

[250] Reynolds SR, Celis E, Sette A, Oratz R, Shapiro RL, Johnston D, et al. HLA-independent heterogeneity of CD8+ T cell responses to MAGE-3, Melan-A/MART-1, gp100, tyrosinase, MC1R, and TRP-2 in vaccine-treated melanoma patients. J Immunol. 1998;161:6970–6. 9862732

[251] Gedye C, Quirk J, Browning J, Svobodová S, John T, Sluka P, et al. Cancer/testis antigens can be immunological targets in clonogenic CD133+ melanoma cells. Cancer Immunol Immunother. 2009;58:1635–46. 19221743 10.1007/s00262-009-0672-0 PMC11029848

[252] Boiko AD, Razorenova OV, van de Rijn M, Swetter SM, Johnson DL, Ly DP, et al. Human melanoma-initiating cells express neural crest nerve growth factor receptor CD271. Nature. 2010;466:133–7. 20596026 10.1038/nature09161 PMC2898751

[253] Schatton T, Frank MH. Antitumor immunity and cancer stem cells. Ann N Y Acad Sci. 2009;1176:154–69. 19796244 10.1111/j.1749-6632.2009.04568.x PMC2893543

[254] Landsberg J, Kohlmeyer J, Renn M, Bald T, Rogava M, Cron M, et al. Melanomas resist T-cell therapy through inflammation-induced reversible dedifferentiation. Nature. 2012;490:412–6. 23051752 10.1038/nature11538

[255] Mehta A, Kim YJ, Robert L, Tsoi J, Comin-Anduix B, Berent-Maoz B, et al. Immunotherapy resistance by inflammation-induced dedifferentiation. Cancer Discov. 2018;8:935–43. 29899062 10.1158/2159-8290.CD-17-1178 PMC6076867

[256] Boshuizen J, Vredevoogd DW, Krijgsman O, Ligtenberg MA, Blankenstein S, de Bruijn B, et al. Reversal of pre-existing NGFR-driven tumor and immune therapy resistance. Nat Commun. 2020;11:3946. 32770055 10.1038/s41467-020-17739-8 PMC7414147

[257] Greenwald RJ, Freeman GJ, Sharpe AH. The B7 family revisited. Annu Rev Immunol. 2005;23:515–48. 15771580 10.1146/annurev.immunol.23.021704.115611

[258] Clarkson MR, Sayegh MH. T-cell costimulatory pathways in allograft rejection and tolerance. Transplantation. 2005;80:555–63. 16177624 10.1097/01.tp.0000168432.60022.99

[259] Lai C, August S, Albibas A, Behar R, Cho SY, Polak ME, et al. OX40+ regulatory T cells in cutaneous squamous cell carcinoma suppress effector T cell responses and associate with metastatic potential. Clin Cancer Res. 2016;22:4236–48. 27034329 10.1158/1078-0432.CCR-15-2614 PMC4987192

[260] Kosmidis M, Dziunycz P, Suárez-Fariñas M, Mühleisen B, Schärer L, Läuchli S, et al. Immunosuppression affects CD4+ mRNA expression and induces Th2 dominance in the microenvironment of cutaneous squamous cell carcinoma in organ transplant recipients. J Immunother. 2010;33:538–46. 20463594 10.1097/CJI.0b013e3181cc2615

[261] Miao Y, Yang H, Levorse J, Yuan S, Polak L, Sribour M, et al. Adaptive immune resistance emerges from tumor-initiating stem cells. Cell. 2019;177:1172-1186.e14. 31031009 10.1016/j.cell.2019.03.025 PMC6525024

[262] Zhang S, Fujita H, Mitsui H, Yanofsky VR, Fuentes-Duculan J, Pettersen JS, et al. Increased Tc22 and Treg/CD8 ratio contribute to aggressive growth of transplant associated squamous cell carcinoma. PLoS ONE. 2013;8: e62154. 23667456 10.1371/journal.pone.0062154 PMC3646982

[263] Frazzette N, Khodadadi-Jamayran A, Doudican N, Santana A, Felsen D, Pavlick AC, et al. Decreased cytotoxic T cells and TCR clonality in organ transplant recipients with squamous cell carcinoma. NPJ Precis Onc. 2020;4:13. 10.1038/s41698-020-0119-9 PMC7270180 32550269

[264] Clark RA, Huang SJ, Murphy GF, Mollet IG, Hijnen D, Muthukuru M, et al. Human squamous cell carcinomas evade the immune response by down-regulation of vascular E-selectin and recruitment of regulatory T cells. J Exp Med. 2008;205:2221–34. 18794336 10.1084/jem.20071190 PMC2556796

[265] Kakizaki A, Fujimura T, Furudate S, Kambayashi Y, Yamauchi T, Yagita H, et al. Immunomodulatory effect of peritumorally administered interferon-beta on melanoma through tumor-associated macrophages. Oncoimmunology. 2015;4: e1047584. 26451326 10.1080/2162402X.2015.1047584 PMC4589056

[266] Pettersen JS, Fuentes-Duculan J, Suárez-Fariñas M, Pierson KC, Pitts-Kiefer A, Fan L, et al. Tumor-associated macrophages in the cutaneous SCC microenvironment are heterogeneously activated. J Invest Dermatol. 2011;131:1322–30. 21307877 10.103/jid.2011.9 PMC3334331

[267] Kambayashi Y, Fujimura T, Aiba S. Comparison of immunosuppressive and immunomodulatory cells in keratoacanthoma and cutaneous squamous cell carcinoma. Acta Derm Venereol. 2013;93:663–8. 23572151 10.2340/00015555-1597

[268] Tjiu J-W, Chen J-S, Shun C-T, Lin S-J, Liao Y-H, Chu C-Y, et al. Tumor-associated macrophage-induced invasion and angiogenesis of human basal cell carcinoma cells by cyclooxygenase-2 induction. J Invest Dermatol. 2009;129:1016–25. 18843292 10.1038/jid.2008.310

[269] Fujimura T, Kakizaki A, Furudate S, Kambayashi Y, Aiba S. Tumor-associated macrophages in skin: How to treat their heterogeneity and plasticity. J Dermatol Sci. 2016;83:167–73. 27291068 10.1016/j.jdermsci.2016.05.015

[270] Yamada K, Uchiyama A, Uehara A, Perera B, Ogino S, Yokoyama Y, et al. MFG-E8 drives melanoma growth by stimulating mesenchymal stromal cell-induced angiogenesis and M2 polarization of tumor-associated macrophages. Cancer Res. 2016;76:4283–92. 27197197 10.1158/0008-5472.CAN-15-2812 PMC5033700

[271] Beksaç B, İlter N, Erdem Ö, Çakmak P, Çenetoğlu S, Yapar D. Sparsity of dendritic cells and cytotoxic T cells in tumor microenvironment may lead to recurrence in basal cell carcinoma. Int J Dermatol. 2020;59:1258–63. 32686125 10.1111/ijd.15065

[272] Linde N, Lederle W, Depner S, van Rooijen N, Gutschalk CM, Mueller MM. Vascular endothelial growth factor-induced skin carcinogenesis depends on recruitment and alternative activation of macrophages. J Pathol. 2012;227:17–28. 22262122 10.1002/path.3989

[273] Falleni M, Savi F, Tosi D, Agape E, Cerri A, Moneghini L, et al. M1 and M2 macrophages’ clinicopathological significance in cutaneous melanoma. Melanoma Res. 2017;27:200–10. 28272106 10.1097/CMR.0000000000000352

[274] Liu N, Zhang J, Yin M, Liu H, Zhang X, Li J, et al. Inhibition of xCT suppresses the efficacy of anti-PD-1/L1 melanoma treatment through exosomal PD-L1-induced macrophage M2 polarization. Mol Ther. 2021;29:2321–34. 33744468 10.1016/j.ymthe.2021.03.013 PMC8261162

[275] Kale S, Raja R, Thorat D, Soundararajan G, Patil TV, Kundu GC. Osteopontin signaling upregulates cyclooxygenase-2 expression in tumor-associated macrophages leading to enhanced angiogenesis and melanoma growth via α9β1 integrin. Oncogene. 2014;33:2295–306. 23728342 10.1038/onc.2013.184

[276] Gehrke S, Otsuka A, Huber R, Meier B, Kistowska M, Fenini G, et al. Metastatic melanoma cell lines do not secrete IL-1β but promote IL-1β production from macrophages. J Dermatol Sci. 2014;74:167–9. 24581590 10.1016/j.jdermsci.2014.01.006

[277] Sevko A, Michels T, Vrohlings M, Umansky L, Beckhove P, Kato M, et al. Antitumor effect of paclitaxel is mediated by inhibition of myeloid-derived suppressor cells and chronic inflammation in the spontaneous melanoma model. J Immunol. 2013;190:2464–71. 23359505 10.4049/jimmunol.1202781 PMC3578135

[278] Wang H, Yang L, Wang D, Zhang Q, Zhang L. Pro-tumor activities of macrophages in the progression of melanoma. Hum Vaccin Immunother. 2017;13:1556–62. 28441072 10.1080/21645515.2017.1312043 PMC5512774

[279] Furudate S, Fujimura T, Kambayashi Y, Kakizaki A, Hidaka T, Aiba S. Immunomodulatory effect of imiquimod through CCL22 produced by tumor-associated macrophages in B16F10 melanomas. Anticancer Res. 2017;37:3461–71. 28668835 10.21873/anticanres.11714

[280] Gutiérrez-Seijo A, García-Martínez E, Barrio-Alonso C, Pareja-Malagón M, Acosta-Ocampo A, Fernández-Santos ME, et al. CCL20/TNF/VEGFA cytokine secretory phenotype of tumor-associated macrophages is a negative prognostic factor in cutaneous melanoma. Cancers. 2021;13:3943. 34439098 10.3390/cancers13163943 PMC8392234

[281] Young HL, Rowling EJ, Bugatti M, Giurisato E, Luheshi N, Arozarena I, et al. An adaptive signaling network in melanoma inflammatory niches confers tolerance to MAPK signaling inhibition. J Exp Med. 2017;214:1691–710. 28450382 10.1084/jem.20160855 PMC5460994

[282] Kumar V, Donthireddy L, Marvel D, Condamine T, Wang F, Lavilla-Alonso S, et al. Cancer-associated fibroblasts neutralize the anti-tumor effect of CSF1 receptor blockade by inducing PMN-MDSC infiltration of tumors. Cancer Cell. 2017;32:654-668.e5. 29136508 10.1016/j.ccell.2017.10.005 PMC5827952

[283] Hu-Lieskovan S, Mok S, Homet Moreno B, Tsoi J, Robert L, Goedert L, et al. Improved antitumor activity of immunotherapy with BRAF and MEK inhibitors in BRAF(V600E) melanoma*.* Sci Transl Med. 2015; 7:279ra41. 10.1126/scitranslmed.aaa4691 PMC4765379 25787767

[284] Fujimura T, Kambayashi Y, Furudate S, Kakizaki A, Haga T, Hashimoto A, et al. Immunomodulatory effects of peplomycin on immunosuppressive and cytotoxic cells in the lesional skin of cutaneous squamous cell carcinoma. Dermatology. 2015;230:250–5. 25678188 10.1159/000369166

[285] Tarhini AA, Zahoor H, Yearley JH, Gibson C, Rahman Z, Dubner R, et al. Tumor associated PD-L1 expression pattern in microscopically tumor positive sentinel lymph nodes in patients with melanoma. J Transl Med. 2015;13:319. 26419843 10.1186/s12967-015-0678-7 PMC4589168

[286] Gordon SR, Maute RL, Dulken BW, Hutter G, George BM, McCracken MN, et al. PD-1 expression by tumour-associated macrophages inhibits phagocytosis and tumour immunity. Nature. 2017;545:495–9. 28514441 10.1038/nature22396 PMC5931375

[287] Fujimura T, Kakizaki A, Kambayashi Y, Sato Y, Tanita K, Lyu C, et al. Cytotoxic antimelanoma drugs suppress the activation of M2 macrophages. Exp Dermatol. 2018;27:64–70. 28833504 10.1111/exd.13417

[288] Tsuruta A, Shiiba Y, Matsunaga N, Fujimoto M, Yoshida Y, Koyanagi S, et al. Diurnal expression of PD-1 on tumor-associated macrophages underlies the dosing time-dependent antitumor effects of the PD-1/PD-L1 Inhibitor BMS-1 in B16/BL6 melanoma-bearing mice. Mol Cancer Res. 2022;20:972–82. 35190830 10.1158/1541-7786.MCR-21-0786 PMC9381128

[289] Tham M, Tan KW, Keeble J, Wang X, Hubert S, Barron L, et al. Melanoma-initiating cells exploit M2 macrophage TGFβ and arginase pathway for survival and proliferation. Oncotarget. 2014;5:12027. 25294815 10.18632/oncotarget.2482 PMC4322977

[290] Ghosh S, Juin SK, Bhattacharyya Majumdar S, Majumdar S. Crucial role of glucosylceramide synthase in the regulation of stem cell-like cancer cells in B16F10 murine melanoma. Mol Carcinog. 2021;60:840–58. 34516706 10.1002/mc.23347

[291] Ohanna M, Giuliano S, Bonet C, Imbert V, Hofman V, Zangari J, et al. Senescent cells develop a PARP-1 and nuclear factor-{kappa}B-associated secretome (PNAS). Genes Dev. 2011;25:1245–61. 21646373 10.1101/gad.625811 PMC3127427

[292] Modestino L, Cristinziano L, Trocchia M, Ventrici A, Capone M, Madonna G, et al. Melanoma-derived soluble mediators modulate neutrophil biological properties and the release of neutrophil extracellular traps. Cancer Immunol Immunother. 2023;72:3363–76. 37525065 10.1007/s00262-023-03493-5 PMC10491523

[293] Ferrucci PF, Ascierto PA, Pigozzo J, Del Vecchio M, Maio M, Cappellini GA, et al. Baseline neutrophils and derived neutrophil-to-lymphocyte ratio: prognostic relevance in metastatic melanoma patients receiving ipilimumab. Ann Oncol. 2016;27:732–8. 26802161 10.1093/annonc/mdw016

[294] Seddon A, Hock B, Miller A, Frei L, Pearson J, McKenzie J, et al. Cutaneous squamous cell carcinomas with markers of increased metastatic risk are associated with elevated numbers of neutrophils and/or granulocytic myeloid derived suppressor cells. J Dermatol Sci. 2016;83:124–30. 27160951 10.1016/j.jdermsci.2016.04.013

[295] Anselmi M, Fontana F, Marzagalli M, Gagliano N, Sommariva M, Limonta P. Melanoma stem cells educate neutrophils to support cancer progression. Cancers. 2022;14:3391. 35884452 10.3390/cancers14143391 PMC9317939

[296] Festa E, Fretz J, Berry R, Schmidt B, Rodeheffer M, Horowitz M, et al. Adipocyte lineage cells contribute to the skin stem cell niche to drive hair cycling. Cell. 2011;146:761–71. 21884937 10.1016/j.cell.2011.07.019 PMC3298746

[297] Guerrero-Juarez CF, Plikus MV. Emerging nonmetabolic functions of skin fat. Nat Rev Endocrinol. 2018;14:163–73. 29327704 10.1038/nrendo.2017.162 PMC6042872

[298] Plikus MV, Mayer JA, de la Cruz D, Baker RE, Maini PK, Maxson R, et al. Cyclic dermal BMP signalling regulates stem cell activation during hair regeneration. Nature. 2008;451:340–4. 18202659 10.1038/nature06457 PMC2696201

[299] Kandyba E, Leung Y, Chen YB, Widelitz R, Chuong CM, Kobielak K. Competitive balance of intrabulge BMP/Wnt signaling reveals a robust gene network ruling stem cell homeostasis and cyclic activation. Proc Natl Acad Sci U S A. 2013;110:1351–6. 23292934 10.1073/pnas.1121312110 PMC3557042

[300] Donati G, Proserpio V, Lichtenberger BM, Natsuga K, Sinclair R, Fujiwara H, et al. Epidermal Wnt/β-catenin signaling regulates adipocyte differentiation via secretion of adipogenic factors. Proc Natl Acad Sci U S A. 2014;111:E1501–9. 24706781 10.1073/pnas.1312880111 PMC3992657

[301] Zhang B, Tsai PC, Gonzalez-Celeiro M, Chung O, Boumard B, Perdigoto CN, et al. Hair follicles’ transit-amplifying cells govern concurrent dermal adipocyte production through Sonic Hedgehog. Genes Dev. 2016;30:2325–38. 27807033 10.1101/gad.285429.116 PMC5110998

[302] Wagner M, Bjerkvig R, Wiig H, Melero-Martin JM, Lin RZ, Klagsbrun M, et al. Inflamed tumor-associated adipose tissue is a depot for macrophages that stimulate tumor growth and angiogenesis. Angiogenesis. 2012;15:481–95. 22614697 10.1007/s10456-012-9276-y PMC3619408

[303] Jung JI, Cho HJ, Jung YJ, Kwon SH, Her S, Choi SS, et al. High-fat diet-induced obesity increases lymphangiogenesis and lymph node metastasis in the B16F10 melanoma allograft model: roles of adipocytes and M2-macrophages. Int J Cancer. 2015;136:258–70. 24844408 10.1002/ijc.28983

[304] Buruiană A, Gheban BA, Gheban-Roșca IA, Georgiu C, Crișan D, Crișan M. The tumor stroma of squamous cell carcinoma: a complex environment that fuels cancer progression. Cancers (Basel). 2024;16:1727. 38730679 10.3390/cancers16091727 PMC11083853

[305] Iwata T, Kuwajima M, Sukeno A, Ishimaru N, Hayashi Y, Wabitsch M, et al. YKL-40 secreted from adipose tissue inhibits degradation of type I collagen. Biochem Biophys Res Commun. 2009;388:511–6. 19666003 10.1016/j.bbrc.2009.08.024

[306] Clement E, Lazar I, Muller C, Nieto L. Obesity and melanoma: could fat be fueling malignancy? Pigment Cell Melanoma Res. 2017;30:294–306. 28222242 10.1111/pcmr.12584

[307] Ma B, Herzog EL, Moore M, Lee C-M, Na SH, Lee CG, et al. RIG-like helicase regulation of chitinase 3-like 1 axis and pulmonary metastasis. Sci Rep. 2016;6:26299. 27198666 10.1038/srep26299 PMC4873814

[308] Li Z, Zhang C, Du JX, Zhao J, Shi MT, Jin MW, et al. Adipocytes promote tumor progression and induce PD-L1 expression via TNF-α/IL-6 signaling. Cancer Cell Int. 2020;20:179. 32477009 10.1186/s12935-020-01269-w PMC7240984

[309] Sun Y, Lodish HF. Adiponectin deficiency promotes tumor growth in mice by reducing macrophage infiltration. PLoS ONE. 2010;5: e11987. 20700533 10.1371/journal.pone.0011987 PMC2916827

[310] Bouche C, Quail DF. Fueling the tumor microenvironment with cancer-associated adipocytes. Cancer Res. 2023;83:1170–2. 37057599 10.1158/0008-5472.CAN-23-0505

[311] Li KN, Jain P, He CH, Eun FC, Kang S, Tumbar T. Skin vasculature and hair follicle cross-talking associated with stem cell activation and tissue homeostasis. Elife. 2019;8: e45977. 31343406 10.7554/eLife.45977 PMC6684267

[312] Xiao Y, Woo WM, Nagao K, Li W, Terunuma A, Mukouyama YS, et al. Perivascular hair follicle stem cells associate with a venule annulus. J Invest Dermatol. 2013;133:2324–31. 23558405 10.1038/jid.2013.167 PMC3742722

[313] Mecklenburg L, Tobin DJ, Müller-Röver S, Handjiski B, Wendt G, Peters EM, et al. Active hair growth (anagen) is associated with angiogenesis. J Invest Dermatol. 2000;114:909–16. 10771470 10.1046/j.1523-1747.2000.00954.x

[314] Botchkarev VA, Sharov AA. BMP signaling in the control of skin development and hair follicle growth. Differentiation. 2004;72:512–26. 15617562 10.1111/j.1432-0436.2004.07209005.x

[315] Wu Z, Bian Y, Chu T, Wang Y, Man S, Song Y, et al. The role of angiogenesis in melanoma: Clinical treatments and future expectations. Front Pharmacol. 2022;13:1028647. 36588679 10.3389/fphar.2022.1028647 PMC9797529

[316] Fania L, Didona D, Di Pietro FR, Verkhovskaia S, Morese R, Paolino G, et al. Cutaneous squamous cell carcinoma: from pathophysiology to novel therapeutic approaches. Biomedicines. 2021;9:171. 33572373 10.3390/biomedicines9020171 PMC7916193

[317] Bowden J, Brennan PA, Umar T, Cronin A. Expression of vascular endothelial growth factor in basal cell carcinoma and cutaneous squamous cell carcinoma of the head and neck. J Cutan Pathol. 2002;29:585–9. 12453295 10.1034/j.1600-0560.2002.291003.x

[318] Malekan M, Haass NK, Rokni GR, Gholizadeh N, Ebrahimzadeh MA, Kazeminejad A. VEGF/VEGFR axis and its signaling in melanoma: Current knowledge toward therapeutic targeting agents and future perspectives. Life Sci. 2024;345: 122563. 38508233 10.1016/j.lfs.2024.122563

[319] López de Andrés J, Ruiz-Toranzo M, Antich C, Chocarro-Wrona C, López-Ruíz E, Jiménez G, et al. Biofabrication of a tri-layered 3D-bioprinted CSC-based malignant melanoma model for personalized cancer treatment*.* Biofabrication. 2023; 15:035016. 10.1088/1758-5090/ac8dc6 36041423

[320] Monzani E, Facchetti F, Galmozzi E, Corsini E, Benetti A, Cavazzin C, et al. Melanoma contains CD133 and ABCG2 positive cells with enhanced tumourigenic potential. Eur J Cancer. 2007;43:935–46. 17320377 10.1016/j.ejca.2007.01.017

[321] Kumar D, Kumar S, Gorain M, Tomar D, Patil HS, Radharani NN, et al. Notch1-MAPK signaling axis regulates CD133+ cancer stem cell-mediated melanoma growth and angiogenesis. J Invest Dermatol. 2016;136:2462–74. 27476721 10.1016/j.jid.2016.07.024

[322] Frank NY, Schatton T, Kim S, Zhan Q, Wilson BJ, Ma J, et al. VEGFR-1 expressed by malignant melanoma-initiating cells is required for tumor growth. Cancer Res. 2011;71:1474–85. 21212411 10.1158/0008-5472.CAN-10-1660 PMC3083845

[323] Zimmerer RM, Matthiesen P, Kreher F, Kampmann A, Spalthoff S, Jehn P, et al. Putative CD133+ melanoma cancer stem cells induce initial angiogenesis in vivo. Microvasc Res. 2016;104:46–54. 26656667 10.1016/j.mvr.2015.12.001

[324] Lee J, Abdeen AA, Hedhli J, Wycislo KL, Dobrucka IT, Fan TM, et al. Melanoma topology reveals a stem-like phenotype that promotes angiogenesis. Sci Adv. 2017;3: e1701350. 29075670 10.1126/sciadv.1701350 PMC5656422

[325] Ciccone V, Terzuoli E, Ristori E, Filippelli A, Ziche M, Morbidelli L, et al. ALDH1A1 overexpression in melanoma cells promotes tumor angiogenesis by activating the IL-8/Notch signaling cascade. Int J Mol Med. 2022;50:99. 35656893 10.3892/ijmm.2022.5155 PMC9186295

[326] Hendrix MJC, Seftor EA, Hess AR, Seftor REB. Vasculogenic mimicry and tumour-cell plasticity: lessons from melanoma. Nat Rev Cancer. 2003;3:411–21. 12778131 10.1038/nrc1092

[327] Lai CY, Schwartz BE, Hsu MY. CD133+ melanoma subpopulations contribute to perivascular niche morphogenesis and tumorigenicity through vasculogenic mimicry. Cancer Res. 2012;72:5111–8. 22865455 10.1158/0008-5472.CAN-12-0624 PMC3463654

[328] Spinella F, Caprara V, Cianfrocca R, Rosanò L, Di Castro V, Garrafa E, et al. The interplay between hypoxia, endothelial and melanoma cells regulates vascularization and cell motility through endothelin-1 and vascular endothelial growth factor. Carcinogenesis. 2014;35:840–8. 24473118 10.1093/carcin/bgu018 PMC3988429

[329] Lee N, Barthel SR, Schatton T. Melanoma stem cells and metastasis: mimicking hematopoietic cell trafficking? Lab Invest. 2014;94:13–30. 24126889 10.1038/labinvest.2013.116 PMC3941309

[330] Beck B, Driessens G, Goossens S, Youssef KK, Kuchnio A, Caauwe A, et al. A vascular niche and a VEGF-Nrp1 loop regulate the initiation and stemness of skin tumours. Nature. 2011;478:399–403. 22012397 10.1038/nature10525

[331] Tzoutzos K, Batistatou A, Kitsos G, Liasko R, Stefanou D. Study of microvascular density and expression of vascular endothelial growth factor and its receptors in cancerous and precancerous lesions of the eyelids. Anticancer Res. 2014;34:4977–83. 25202080

[332] Benwell CJ, Johnson RT, Taylor J, Price CA, Robinson SD. Endothelial VEGFR coreceptors neuropilin-1 and neuropilin-2 are essential for tumor angiogenesis. Cancer Res Commun. 2022;2:1626–40. 36970722 10.1158/2767-9764.CRC-22-0250 PMC10036134

[333] Wang X, Liu Y, He J, Wang J, Chen X, and Yang R. Regulation of signaling pathways in hair follicle stem cells. Burns Trauma. 2022; 10:tkac022. 10.1093/burnst/tkac022 PMC9250793 35795256

[334] Park HL, Bai C, Platt KA, Matise MP, Beeghly A, Hui Cc, et al. Mouse Gli1 mutants are viable but have defects in SHH signaling in combination with a Gli2 mutation*.* Development. 2000; 127:1593–1605. 10.1242/dev.127.8.1593 10725236

[335] Mill P, Mo R, Hu MC, Dagnino L, Rosenblum ND, Hui CC. Shh controls epithelial proliferation via independent pathways that converge on N-Myc. Dev Cell. 2005;9:293–303. 16054035 10.1016/j.devcel.2005.05.009

[336] Driskell RR, Giangreco A, Jensen KB, Mulder KW, Watt FM. Sox2-positive dermal papilla cells specify hair follicle type in mammalian epidermis. Development. 2009;136:2815–23. 19605494 10.1242/dev.038620 PMC2730408

[337] Duman-Scheel M, Weng L, Xin S, Du W. Hedgehog regulates cell growth and proliferation by inducing Cyclin D and Cyclin E. Nature. 2002;417:299–304. 12015606 10.1038/417299a

[338] Adolphe C, Nieuwenhuis E, Villani R, Li ZJ, Kaur P, Hui C-c, et al. Patched 1 and patched 2 redundancy has a key role in regulating epidermal differentiation*.* J Invest Dermatol. 2014; 134:1981–1990. 10.1038/jid.2014.63 24492243

[339] Jeong J, McMahon AP. Growth and pattern of the mammalian neural tube are governed by partially overlapping feedback activities of the hedgehog antagonists patched 1 and Hhip1. Development. 2005;132:143–54. 15576403 10.1242/dev.01566

[340] Adolphe C, Junker JP, Lyubimova A, van Oudenaarden A, Wainwright B. Patched receptors sense, interpret, and establish an epidermal hedgehog signaling gradient. J Invest Dermatol. 2017;137:179–86. 27498049 10.1016/j.jid.2016.06.632

[341] Celso CL, Prowse DM, Watt FM. Transient activation of β-catenin signalling in adult mouse epidermis is sufficient to induce new hair follicles but continuous activation is required to maintain hair follicle tumours. Development. 2004;131:1787–99. 15084463 10.1242/dev.01052

[342] Suen WJ, Li ST, Yang LT. Hes1 regulates anagen initiation and hair follicle regeneration through modulation of hedgehog signaling. Stem Cells. 2020;38:301–14. 31721388 10.1002/stem.3117 PMC7027765

[343] Reddy S, Andl T, Bagasra A, Lu MM, Epstein DJ, Morrisey EE, et al. Characterization of Wnt gene expression in developing and postnatal hair follicles and identification of Wnt5a as a target of Sonic hedgehog in hair follicle morphogenesis. Mech Dev. 2001;107:69–82. 11520664 10.1016/s0925-4773(01)00452-x

[344] Kwack MH, Kim MK, Kim JC, Sung YK. Wnt5a attenuates Wnt/β-catenin signalling in human dermal papilla cells. Exp Dermatol. 2013;22:229–31. 23489428 10.1111/exd.12101

[345] Clevers H, Loh KM, Nusse R. An integral program for tissue renewal and regeneration: Wnt signaling and stem cell control. Science. 2014;346:1248012. 25278615 10.1126/science.1248012

[346] Lim X, Tan SH, Koh WLC, Chau RMW, Yan KS, Kuo CJ, et al. Interfollicular epidermal stem cells self-renew via autocrine Wnt signaling. Science. 2013;342:1226–30. 24311688 10.1126/science.1239730 PMC4081860

[347] Leng L, Ma J, Lv L, Wang W, Gao D, Zhu Y, et al. Both Wnt signaling and epidermal stem cell-derived extracellular vesicles are involved in epidermal cell growth. Stem Cell Res Ther. 2020;11:1–11. 32967725 10.1186/s13287-020-01933-y PMC7510321

[348] Lim X, Tan SH, Yu KL, Lim SB, Nusse R. Axin2 marks quiescent hair follicle bulge stem cells that are maintained by autocrine Wnt/β-catenin signaling. Proc Natl Acad Sci U S A. 2016;113:E1498–1505. 26903625 10.1073/pnas.1601599113 PMC4801317

[349] Vance KW, Goding CR. The transcription network regulating melanocyte development and melanoma. Pigment Cell Res. 2004;17:318–25. 15250933 10.1111/j.1600-0749.2004.00164.x

[350] Totaro A, Castellan M, Battilana G, Zanconato F, Azzolin L, Giulitti S, et al. YAP/TAZ link cell mechanics to Notch signalling to control epidermal stem cell fate. Nat Commun. 2017;8:1–13. 28513598 10.1038/ncomms15206 PMC5442321

[351] Zhang H, Pasolli HA, Fuchs E. Yes-associated protein (YAP) transcriptional coactivator functions in balancing growth and differentiation in skin. Proc Natl Acad Sci U S A. 2011;108:2270–5. 21262812 10.1073/pnas.1019603108 PMC3038759

[352] De Rosa L, Seconetti AS, De Santis G, Pellacani G, Hirsch T, Rothoeft T, et al. Laminin 332-dependent YAP dysregulation depletes epidermal stem cells in junctional epidermolysis bullosa. Cell Rep. 2019;27:2036-2049.e6. 31091444 10.1016/j.celrep.2019.04.055

[353] Kenny FN, Drymoussi Z, Delaine-Smith R, Kao AP, Laly AC, Knight MM, et al. Tissue stiffening promotes keratinocyte proliferation through activation of epidermal growth factor signaling*.* J Cell Sci. 2018; 131:jcs215780. 10.1242/jcs.215780 29669739

[354] Benham-Pyle BW, Pruitt BL, Nelson WJ. Mechanical strain induces E-cadherin–dependent Yap1 and β-catenin activation to drive cell cycle entry. Science. 2015;348:1024–7. 26023140 10.1126/science.aaa4559 PMC4572847

[355] Ya C, Carrancá M, Sigaudo-Roussel D, Faure P, Fromy B, Debret R. Substrate softness promotes terminal differentiation of human keratinocytes without altering their ability to proliferate back into a rigid environment. Arch Dermatol Res. 2019;311:741–51. 31392392 10.1007/s00403-019-01962-5

[356] Elbediwy A, Vincent-Mistiaen ZI, Spencer-Dene B, Stone RK, Boeing S, Wculek SK, et al. Integrin signalling regulates YAP and TAZ to control skin homeostasis. Development. 2016;143:1674–87. 26989177 10.1242/dev.133728 PMC4874484

[357] Sambandam SA, Kasetti RB, Xue L, Dean DC, Lu Q, Li Q. 14-3-3σ regulates keratinocyte proliferation and differentiation by modulating Yap1 cellular localization. J Invest Dermatol. 2015;135:1621–8. 25668240 10.1038/jid.2015.42 PMC4430425

[358] Akladios B, Mendoza Reinoso V, Cain JE, Wang T, Lambie DL, Watkins DN, et al. Positive regulatory interactions between YAP and Hedgehog signalling in skin homeostasis and BCC development in mouse skin in vivo. PLoS ONE. 2017;12: e0183178. 28820907 10.1371/journal.pone.0183178 PMC5562304

[359] Mendoza-Reinoso V, Beverdam A. Epidermal YAP activity drives canonical WNT16/β-catenin signaling to promote keratinocyte proliferation in vitro and in the murine skin. Stem Cell Res. 2018;29:15–23. 29562208 10.1016/j.scr.2018.03.005

[360] Akladios B, Mendoza-Reinoso V, Samuel MS, Hardeman EC, Khosrotehrani K, Key B, et al. Epidermal YAP2-5SA-ΔC drives β-catenin activation to promote keratinocyte proliferation in mouse skin in vivo. J Invest Dermatol. 2017;137:716–26. 27816394 10.1016/j.jid.2016.10.029

[361] Rangarajan A, Talora C, Okuyama R, Nicolas M, Mammucari C, Oh H, et al. Notch signaling is a direct determinant of keratinocyte growth arrest and entry into differentiation. EMBO J. 2001;20:3427–36. 11432830 10.1093/emboj/20.13.3427 PMC125257

[362] Devgan V, Mammucari C, Millar SE, Brisken C, Dotto GP. p21WAF1/Cip1 is a negative transcriptional regulator of Wnt4 expression downstream of Notch1 activation. Genes Dev. 2005;19:1485–95. 15964998 10.1101/gad.341405 PMC1151665

[363] Negri VA, Logtenberg MEW, Renz LM, Oules B, Walko G, Watt FM. Delta-like 1-mediated cis-inhibition of Jagged1/2 signalling inhibits differentiation of human epidermal cells in culture. Sci Rep. 2019;9:10825. 31346203 10.1038/s41598-019-47232-2 PMC6658703

[364] Negri VA, Louis B, Zijl S, Ganier C, Philippeos C, Ali S, et al. Single-cell RNA sequencing of human epidermis identifies Lunatic fringe as a novel regulator of the stem cell compartment. Stem Cell Reports. 2023;18:2047–55. 37832539 10.1016/j.stemcr.2023.09.007 PMC10679657

[365] Watt FM, Estrach S, Ambler CA. Epidermal Notch signalling: differentiation, cancer and adhesion. Curr Opin Cell Biol. 2008;20:171–9. 18342499 10.1016/j.ceb.2008.01.010 PMC2324124

[366] Lefort K, Mandinova A, Ostano P, Kolev V, Calpini V, Kolfschoten I, et al. Notch1 is a p53 target gene involved in human keratinocyte tumor suppression through negative regulation of ROCK1/2 and MRCKα kinases. Genes Dev. 2007;21:562–77. 17344417 10.1101/gad.1484707 PMC1820898

[367] Nguyen B-C, Lefort K, Mandinova A, Antonini D, Devgan V, Della Gatta G, et al. Cross-regulation between Notch and p63 in keratinocyte commitment to differentiation. Genes Dev. 2006;20:1028–42. 16618808 10.1101/gad.1406006 PMC1472299

[368] Restivo G, Nguyen BC, Dziunycz P, Ristorcelli E, Ryan RJ, Özuysal ÖY, et al. IRF6 is a mediator of Notch pro-differentiation and tumour suppressive function in keratinocytes. EMBO J. 2011;30:4571–85. 21909072 10.1038/emboj.2011.325 PMC3243593

[369] Moriyama M, Osawa M, Mak S-S, Ohtsuka T, Yamamoto N, Han H, et al. Notch signaling via Hes1 transcription factor maintains survival of melanoblasts and melanocyte stem cells. J Cell Biol. 2006;173:333–9. 16651378 10.1083/jcb.200509084 PMC2063834

[370] Kakuda S, Haltiwanger RS. Deciphering the fringe-mediated Notch code: identification of activating and inhibiting sites allowing discrimination between ligands. Dev Cell. 2017;40:193–201. 28089369 10.1016/j.devcel.2016.12.013 PMC5263050

[371] Lowell S, Jones P, Le Roux I, Dunne J, Watt FM. Stimulation of human epidermal differentiation by Delta-Notch signalling at the boundaries of stem-cell clusters. Curr Biol. 2000;10:491–500. 10801437 10.1016/s0960-9822(00)00451-6

[372] Calautti E, Li J, Saoncella S, Brissette JL, Goetinck PF. Phosphoinositide 3-kinase signaling to Akt promotes keratinocyte differentiation versus death. J Biol Chem. 2005;280:32856–65. 16036919 10.1074/jbc.M506119200

[373] Wang X, Chen H, Tian R, Zhang Y, Drutskaya MS, Wang C, et al. Macrophages induce AKT/β-catenin-dependent Lgr5(+) stem cell activation and hair follicle regeneration through TNF. Nat Commun. 2017;8:14091. 28345588 10.1038/ncomms14091 PMC5378973

[374] Murayama K, Kimura T, Tarutani M, Tomooka M, Hayashi R, Okabe M, et al. Akt activation induces epidermal hyperplasia and proliferation of epidermal progenitors. Oncogene. 2007;26:4882–8. 17297448 10.1038/sj.onc.1210274

[375] Sano S, Itami S, Takeda K, Tarutani M, Yamaguchi Y, Miura H, et al. Keratinocyte-specific ablation of Stat3 exhibits impaired skin remodeling, but does not affect skin morphogenesis. EMBO J. 1999;18:4657–68. 10469645 10.1093/emboj/18.17.4657 PMC1171539

[376] Sano S, Chan KS, DiGiovanni J. Impact of Stat3 activation upon skin biology: a dichotomy of its role between homeostasis and diseases. J Dermatol Sci. 2008;50:1–14. 17601706 10.1016/j.jdermsci.2007.05.016

[377] Miettinen PJ, Berger JE, Meneses J, Phung Y, Pedersen RA, Werb Z, et al. Epithelial immaturity and multiorgan failure in mice lacking epidermal growth factor receptor. Nature. 1995;376:337–41. 7630400 10.1038/376337a0

[378] Davies MA. The role of the PI3K-AKT pathway in melanoma. Cancer J. 2012;18:142–7. 22453015 10.1097/PPO.0b013e31824d448c

[379] Chamcheu JC, Roy T, Uddin MB, Banang-Mbeumi S, Chamcheu RN, Walker AL, et al. Role and therapeutic targeting of the PI3K/Akt/mTOR signaling pathway in skin cancer: a review of current status and future trends on natural and synthetic agents therapy. Cells. 2019;8:803. 31370278 10.3390/cells8080803 PMC6721560

[380] Lin KK, Chudova D, Hatfield GW, Smyth P, Andersen B. Identification of hair cycle-associated genes from time-course gene expression profile data by using replicate variance. Proc Natl Acad Sci U S A. 2004;101:15955–60. 15520371 10.1073/pnas.0407114101 PMC524696

[381] Doles J, Storer M, Cozzuto L, Roma G, Keyes WM. Age-associated inflammation inhibits epidermal stem cell function. Genes Dev. 2012;26:2144–53. 22972935 10.1101/gad.192294.112 PMC3465736

[382] Morris R, Kershaw NJ, Babon JJ. The molecular details of cytokine signaling via the JAK/STAT pathway. Protein Sci. 2018;27:1984–2009. 30267440 10.1002/pro.3519 PMC6237706

[383] Rébé C, Végran F, Berger H, Ghiringhelli F. STAT3 activation: A key factor in tumor immunoescape. Jakstat. 2013;2: e23010. 24058791 10.4161/jkst.23010 PMC3670267

[384] Gu D, Fan Q, Zhang X, Xie J. A role for transcription factor STAT3 signaling in oncogene smoothened-driven carcinogenesis. J Biol Chem. 2012;287:38356–66. 22992748 10.1074/jbc.M112.377382 PMC3488104

[385] Hauser PJ, Agrawal D, Hackney J, Pledger WJ. STAT3 activation accompanies keratinocyte differentiation. Cell Growth Differ. 1998;9:847–55. 9790496

[386] Kira M, Sano S, Takagi S, Yoshikawa K, Takeda J, Itami S. STAT3 deficiency in keratinocytes leads to compromised cell migration through hyperphosphorylation of p130(cas). J Biol Chem. 2002;277:12931–6. 11812786 10.1074/jbc.M110795200

[387] Rao D, Macias E, Carbajal S, Kiguchi K, DiGiovanni J. Constitutive Stat3 activation alters behavior of hair follicle stem and progenitor cell populations. Mol Carcinog. 2015;54:121–33. 24038534 10.1002/mc.22080 PMC4139480

[388] Santini R, Vinci MC, Pandolfi S, Penachioni JY, Montagnani V, Olivito B, et al. Hedgehog-GLI signaling drives self-renewal and tumorigenicity of human melanoma-initiating cells. Stem Cells. 2012;30:1808–18. 22730244 10.1002/stem.1160

[389] Pandolfi S, Montagnani V, Penachioni J, Vinci M, Olivito B, Borgognoni L, et al. WIP1 phosphatase modulates the Hedgehog signaling by enhancing GLI1 function. Oncogene. 2013;32:4737–47. 23146903 10.1038/onc.2012.502

[390] Epstein EH. Basal cell carcinomas: attack of the hedgehog. Nat Rev Cancer. 2008;8:743–54. 18813320 10.1038/nrc2503 PMC4457317

[391] Kim HS, Kim YS, Lee C, Shin MS, Kim JW, Jang BG. Expression profile of sonic hedgehog signaling-related molecules in basal cell carcinoma. PLoS ONE. 2019;14: e0225511. 31756206 10.1371/journal.pone.0225511 PMC6874381

[392] Barker N, Van Es J, Jaks V, Kasper M, Snippert H, Toftgård R, et al. Very long-term self-renewal of small intestine, colon, and hair follicles from cycling Lgr5+ ve stem cells. Cold Spring Harb Symp Quant Biol. 2008;73:351–6. 19478326 10.1101/sqb.2008.72.003

[393] Malanchi I, Peinado H, Kassen D, Hussenet T, Metzger D, Chambon P, et al. Cutaneous cancer stem cell maintenance is dependent on β-catenin signalling. Nature. 2008;452:650–3. 18385740 10.1038/nature06835

[394] Yang GN, Strudwick XL, Bonder CS, Kopecki Z, and Cowin AJ. Increased expression of flightless I in cutaneous squamous cell carcinoma affects Wnt/β-catenin signaling pathway*.* Int J Mol Sci. 2021; 22. 10.3390/ijms222413203 PMC8703548 34948000

[395] Wei CY, Zhu MX, Yang YW, Zhang PF, Yang X, Peng R, et al. Downregulation of RNF128 activates Wnt/β-catenin signaling to induce cellular EMT and stemness via CD44 and CTTN ubiquitination in melanoma. J Hematol Oncol. 2019;12:21. 30832692 10.1186/s13045-019-0711-z PMC6399928

[396] Rappa G, Mercapide J, Anzanello F, Le TT, Johlfs MG, Fiscus RR, et al. Wnt interaction and extracellular release of prominin-1/CD133 in human malignant melanoma cells. Exp Cell Res. 2013;319:810–9. 23318676 10.1016/j.yexcr.2013.01.003 PMC3594006

[397] Li J, Fang R, Wu J, Si Y, Bai J, Wang Q. The NOP14 nucleolar protein suppresses the function and stemness of melanoma stem-like cells through Wnt/beta-catenin signaling inactivation. Bioengineered. 2022;13:7648–58. 35282769 10.1080/21655979.2022.2050491 PMC9208496

[398] Yang PT, Anastas JN, Toroni RA, Shinohara MM, Goodson JM, Bosserhoff AK, et al. WLS inhibits melanoma cell proliferation through the β-catenin signalling pathway and induces spontaneous metastasis. EMBO Mol Med. 2012;4:1294–307. 23129487 10.1002/emmm.201201486 PMC3531604

[399] Dissanayake SK, Wade M, Johnson CE, O’Connell MP, Leotlela PD, French AD, et al. The Wnt5A/protein kinase C pathway mediates motility in melanoma cells via the inhibition of metastasis suppressors and initiation of an epithelial to mesenchymal transition. J Biol Chem. 2007;282:17259–71. 17426020 10.1074/jbc.M700075200 PMC2263117

[400] Medici D, Hay ED, Olsen BR. Snail and Slug promote epithelial-mesenchymal transition through beta-catenin-T-cell factor-4-dependent expression of transforming growth factor-beta3. Mol Biol Cell. 2008;19:4875–87. 18799618 10.1091/mbc.E08-05-0506 PMC2575183

[401] Ryu HJ, Kim C, Jang H, Kim SI, Shin SJ, Chung KY, et al. Nuclear localization of yes-associated protein is associated with tumor progression in cutaneous melanoma. Lab Invest. 2024;104: 102048. 38490470 10.1016/j.labinv.2024.102048

[402] Jia J, Li C, Luo S, Liu-Smith F, Yang J, Wang X, et al. Yes-associated protein contributes to the development of human cutaneous squamous cell carcinoma via activation of RAS. J Invest Dermatol. 2016;136:1267–77. 26902922 10.1016/j.jid.2016.02.005

[403] Maglic D, Schlegelmilch K, Dost AF, Panero R, Dill MT, Calogero RA, et al. YAP-TEAD signaling promotes basal cell carcinoma development via ac-JUN/AP1 axis. EMBO J. 2018;37: e98642. 30037824 10.15252/embj.201798642 PMC6120663

[404] Quan T, Xu Y, Qin Z, Robichaud P, Betcher S, Calderone K, et al. Elevated YAP and its downstream targets CCN1 and CCN2 in basal cell carcinoma: impact on keratinocyte proliferation and stromal cell activation. Am J Pathol. 2014;184:937–43. 24485923 10.1016/j.ajpath.2013.12.017 PMC3969992

[405] Miskolczi Z, Smith MP, Rowling EJ, Ferguson J, Barriuso J, Wellbrock C. Collagen abundance controls melanoma phenotypes through lineage-specific microenvironment sensing. Oncogene. 2018;37:3166–82. 29545604 10.1038/s41388-018-0209-0 PMC5992128

[406] Manderfield LJ, Engleka KA, Aghajanian H, Gupta M, Yang S, Li L, et al. Pax3 and hippo signaling coordinate melanocyte gene expression in neural crest. Cell Rep. 2014;9:1885–95. 25466249 10.1016/j.celrep.2014.10.061 PMC4267159

[407] Yang G, Li Y, Nishimura EK, Xin H, Zhou A, Guo Y, et al. Inhibition of PAX3 by TGF-beta modulates melanocyte viability. Mol Cell. 2008;32:554–63. 19026785 10.1016/j.molcel.2008.11.002

[408] Braig S, Wallner S, Junglas B, Fuchshofer R, Bosserhoff AK. CTGF is overexpressed in malignant melanoma and promotes cell invasion and migration. Br J Cancer. 2011;105:231–8. 21673687 10.1038/bjc.2011.226 PMC3142806

[409] Al-Rohil RN, Tarasen AJ, Carlson JA, Wang K, Johnson A, Yelensky R, et al. Evaluation of 122 advanced-stage cutaneous squamous cell carcinomas by comprehensive genomic profiling opens the door for new routes to targeted therapies. Cancer. 2016;122:249–57. 26479420 10.1002/cncr.29738

[410] Chang D, Shain AH. The landscape of driver mutations in cutaneous squamous cell carcinoma. NPJ Genom Med. 2021;6:61. 34272401 10.1038/s41525-021-00226-4 PMC8285521

[411] Bonilla X, Parmentier L, King B, Bezrukov F, Kaya G, Zoete V, et al. Genomic analysis identifies new drivers and progression pathways in skin basal cell carcinoma. Nat Genet. 2016;48:398–406. 26950094 10.1038/ng.3525

[412] Nicolas M, Wolfer A, Raj K, Kummer JA, Mill P, van Noort M, et al. Notch1 functions as a tumor suppressor in mouse skin. Nat Genet. 2003;33:416–21. 12590261 10.1038/ng1099

[413] Pickering CR, Zhou JH, Lee JJ, Drummond JA, Peng SA, Saade RE, et al. Mutational landscape of aggressive cutaneous squamous cell carcinoma. Clin Cancer Res. 2014;20:6582–92. 25303977 10.1158/1078-0432.CCR-14-1768 PMC4367811

[414] Wang NJ, Sanborn Z, Arnett KL, Bayston LJ, Liao W, Proby CM, et al. Loss-of-function mutations in Notch receptors in cutaneous and lung squamous cell carcinoma. Proc Natl Acad Sci U S A. 2011;108:17761–6. 22006338 10.1073/pnas.1114669108 PMC3203814

[415] Cordle J, Redfieldz C, Stacey M, van der Merwe PA, Willis AC, Champion BR, et al. Localization of the delta-like-1-binding site in human Notch-1 and its modulation by calcium affinity. J Biol Chem. 2008;283:11785–93. 18296446 10.1074/jbc.M708424200

[416] Nam Y, Sliz P, Song L, Aster JC, Blacklow SC. Structural basis for cooperativity in recruitment of MAML coactivators to Notch transcription complexes. Cell. 2006;124:973–83. 16530044 10.1016/j.cell.2005.12.037

[417] Oswald F, Täuber B, Dobner T, Bourteele S, Kostezka U, Adler G, et al. p300 acts as a transcriptional coactivator for mammalian Notch-1. Mol Cell Biol. 2001;21:7761–74. 11604511 10.1128/MCB.21.22.7761-7774.2001 PMC99946

[418] Quan XX, Hawk NV, Chen W, Coupar J, Lee SK, Petersen DW, et al. Targeting notch1 and IKKα enhanced NF-κB activation in CD133+ skin cancer stem cells. Mol Cancer Ther. 2018;17:2034–48. 29959199 10.1158/1535-7163.MCT-17-0421 PMC6461743

[419] Bedogni B, Warneke JA, Nickoloff BJ, Giaccia AJ, Powell MB. Notch1 is an effector of Akt and hypoxia in melanoma development. J Clin Invest. 2008;118:3660–70. 18924608 10.1172/JCI36157 PMC2567838

[420] Kaushik G, Venugopal A, Ramamoorthy P, Standing D, Subramaniam D, Umar S, et al. Honokiol inhibits melanoma stem cells by targeting Notch signaling. Mol Carcinog. 2015;54:1710–21. 25491779 10.1002/mc.22242 PMC4776032

[421] Ronchini C, Capobianco AJ. Induction of cyclin D1 transcription and CDK2 activity by Notch(ic): implication for cell cycle disruption in transformation by Notch(ic). Mol Cell Biol. 2001;21:5925–34. 11486031 10.1128/MCB.21.17.5925-5934.2001 PMC87311

[422] Gustafsson MV, Zheng X, Pereira T, Gradin K, Jin S, Lundkvist J, et al. Hypoxia requires notch signaling to maintain the undifferentiated cell state. Dev Cell. 2005;9:617–28. 16256737 10.1016/j.devcel.2005.09.010

[423] Lin X, Sun B, Zhu D, Zhao X, Sun R, Zhang Y, et al. Notch4+ cancer stem-like cells promote the metastatic and invasive ability of melanoma. Cancer Sci. 2016;107:1079–91. 27234159 10.1111/cas.12978 PMC4982579

[424] Hsu M-Y, Yang MH, Schnegg CI, Hwang S, Ryu B, Alani RM. Notch3 signaling-mediated melanoma–endothelial crosstalk regulates melanoma stem-like cell homeostasis and niche morphogenesis. Lab Invest. 2017;97:725–36. 28165469 10.1038/labinvest.2017.1 PMC5446297

[425] Hardy KM, Kirschmann DA, Seftor EA, Margaryan NV, Postovit LM, Strizzi L, et al. Regulation of the embryonic morphogen Nodal by Notch4 facilitates manifestation of the aggressive melanoma phenotype. Cancer Res. 2010;70:10340–50. 21159651 10.1158/0008-5472.CAN-10-0705 PMC3057934

[426] Kakadia S, Yarlagadda N, Awad R, Kundranda M, Niu J, Naraev B, et al. Mechanisms of resistance to BRAF and MEK inhibitors and clinical update of US Food and Drug Administration-approved targeted therapy in advanced melanoma. Onco Targets Ther. 2018;11:7095–107. 30410366 10.2147/OTT.S182721 PMC6200076

[427] Segrelles C, García-Escudero R, Garín MI, Aranda JF, Hernández P, Ariza JM, et al. Akt signaling leads to stem cell activation and promotes tumor development in epidermis. Stem Cells. 2014;32:1917–28. 24504902 10.1002/stem.1669

[428] Chan KS, Carbajal S, Kiguchi K, Clifford J, Sano S, DiGiovanni J. Epidermal growth factor receptor-mediated activation of Stat3 during multistage skin carcinogenesis. Cancer Res. 2004;64:2382–9. 15059889 10.1158/0008-5472.can-03-3197

[429] Niu G, Bowman T, Huang M, Shivers S, Reintgen D, Daud A, et al. Roles of activated Src and Stat3 signaling in melanoma tumor cell growth. Oncogene. 2002;21:7001–10. 12370822 10.1038/sj.onc.1205859

[430] Waterman EA, Sakai N, Nguyen NT, Horst BA, Veitch DP, Dey CN, et al. A laminin-collagen complex drives human epidermal carcinogenesis through phosphoinositol-3-kinase activation. Cancer Res. 2007;67:4264–70. 17483338 10.1158/0008-5472.CAN-06-4141

[431] Yuan S, Stewart KS, Yang Y, Abdusselamoglu MD, Parigi SM, Feinberg TY, et al. Ras drives malignancy through stem cell crosstalk with the microenvironment. Nature. 2022;612:555–63. 36450983 10.1038/s41586-022-05475-6 PMC9750880

[432] Jamal SME, Alamodi A, Wahl RU, Grada Z, Shareef MA, Hassan S-Y, et al. Melanoma stem cell maintenance and chemo-resistance are mediated by CD133 signal to PI3K-dependent pathways. Oncogene. 2020;39:5468–78. 32616888 10.1038/s41388-020-1373-6

[433] Murai T, Matsuda S. Targeting the PI3K-Akt-mTOR signaling pathway involved in vasculogenic mimicry promoted by cancer stem cells. Am J Cancer Res. 2023;13:5039–46. 38058805 PMC10695779

[434] De Andrea M, Rittà M, Landini MM, Borgogna C, Mondini M, Kern F, et al. Keratinocyte-specific stat3 heterozygosity impairs development of skin tumors in human papillomavirus 8 transgenic mice. Cancer Res. 2010;70:7938–48. 20876801 10.1158/0008-5472.CAN-10-1128

[435] Kataoka K, Kim DJ, Carbajal S, Clifford JL, DiGiovanni J. Stage-specific disruption of Stat3 demonstrates a direct requirement during both the initiation and promotion stages of mouse skin tumorigenesis. Carcinogenesis. 2008;29:1108–14. 18453544 10.1093/carcin/bgn061 PMC2902397

[436] Kim DJ, Kataoka K, Rao D, Kiguchi K, Cotsarelis G, Digiovanni J. Targeted disruption of Stat3 reveals a major role for follicular stem cells in skin tumor initiation. Cancer Res. 2009;69:7587–94. 19738054 10.1158/0008-5472.CAN-09-1180 PMC4043290

[437] Suiqing C, Min Z, Lirong C. Overexpression of phosphorylated-STAT3 correlated with the invasion and metastasis of cutaneous squamous cell carcinoma. J Dermatol. 2005;32:354–60. 16043897 10.1111/j.1346-8138.2005.tb00906.x

[438] Clifford JL, Walch E, Yang X, Xu X, Alberts DS, Clayman GL, et al. Suppression of type I interferon signaling proteins is an early event in squamous skin carcinogenesis. Clin Cancer Res. 2002;8:2067–72. 12114405

[439] Syed ZA, Yin W, Hughes K, Gill JN, Shi R, Clifford JL. HGF/c-met/Stat3 signaling during skin tumor cell invasion: indications for a positive feedback loop. BMC Cancer. 2011;11:180. 21595927 10.1186/1471-2407-11-180 PMC3112164

[440] Chen SY, Takeuchi S, Moroi Y, Hayashida S, Kido M, Chen SJ, et al. Overexpression of phosphorylated-ATF2 and STAT3 in cutaneous squamous cell carcinoma, Bowen’s disease and basal cell carcinoma. J Dermatol Sci. 2008;51:210–5. 18547788 10.1016/j.jdermsci.2008.04.008

[441] Cai SQ, Chen LR, Wang HJ, Yao LF, Zheng M. Effect of STAT3 phosphorylation and p53 expression on human epidermal non melanoma cutaneous tumors. Zhejiang Da Xue Xue Bao Yi Xue Ban. 2004;33:331–4. 15269985 10.3785/j.issn.1008-9292.2004.04.012

[442] Xie TX, Wei D, Liu M, Gao AC, Ali-Osman F, Sawaya R, et al. Stat3 activation regulates the expression of matrix metalloproteinase-2 and tumor invasion and metastasis. Oncogene. 2004;23:3550–60. 15116091 10.1038/sj.onc.1207383

[443] Wu ZS, Cheng XW, Wang XN, Song NJ. Prognostic significance of phosphorylated signal transducer and activator of transcription 3 and suppressor of cytokine signaling 3 expression in human cutaneous melanoma. Melanoma Res. 2011;21:483–90. 21876460 10.1097/CMR.0b013e32834acc37

[444] Chan KS, Sano S, Kataoka K, Abel E, Carbajal S, Beltran L, et al. Forced expression of a constitutively active form of Stat3 in mouse epidermis enhances malignant progression of skin tumors induced by two-stage carcinogenesis. Oncogene. 2008;27:1087–94. 17700521 10.1038/sj.onc.1210726

[445] Yin W, Cheepala S, Roberts JN, Syson-Chan K, DiGiovanni J, Clifford JL. Active Stat3 is required for survival of human squamous cell carcinoma cells in serum-free conditions. Mol Cancer. 2006;5:15. 16603078 10.1186/1476-4598-5-15 PMC1502137

[446] Kang Y, Massagué J. Epithelial-mesenchymal transitions: Twist in development and metastasis. Cell. 2004;118:277–9. 15294153 10.1016/j.cell.2004.07.011

[447] Badarinath K, Dam B, Kataria S, Zirmire RK, Dey R, Kansagara G, et al. Snail maintains the stem/progenitor state of skin epithelial cells and carcinomas through the autocrine effect of matricellular protein Mindin. Cell Rep. 2022;40: 111390. 36130502 10.1016/j.celrep.2022.111390

[448] Du F, Nakamura Y, Tan TL, Lee P, Lee R, Yu B, et al. Expression of Snail in epidermal keratinocytes promotes cutaneous inflammation and hyperplasia conducive to tumor formation. Cancer Res. 2010;70:10080–9. 21159631 10.1158/0008-5472.CAN-10-0324 PMC5559295

[449] Rana I, Kataria S, Tan TL, Hajam EY, Kashyap DK, Saha D, et al. Mindin (SPON2) Is essential for cutaneous fibrogenesis in a mouse model of systemic sclerosis. J Invest Dermatol. 2023;143:699-710.e10. 36528128 10.1016/j.jid.2022.10.011

[450] Mullor JL, Dahmane N, Sun T, and Ruiz i Altaba A. Wnt signals are targets and mediators of Gli function. Curr Biol. 2001; 11:769–773. 10.1016/s0960-9822(01)00229-9 11378387

[451] Yang SH, Andl T, Grachtchouk V, Wang A, Liu J, Syu LJ, et al. Pathological responses to oncogenic Hedgehog signaling in skin are dependent on canonical Wnt/beta3-catenin signaling. Nat Genet. 2008;40:1130–5. 19165927 10.1038/ng.192 PMC2688690

[452] Kuphal S, Lodermeyer S, Bataille F, Schuierer M, Hoang BH, Bosserhoff AK. Expression of Dickkopf genes is strongly reduced in malignant melanoma. Oncogene. 2006;25:5027–36. 16568085 10.1038/sj.onc.1209508

[453] Zhang JP, Li N, Bai WZ, Qiu XC, Ma BA, Zhou Y, et al. Notch ligand Delta-like 1 promotes the metastasis of melanoma by enhancing tumor adhesion. Braz J Med Biol Res. 2014;47:299–306. 24714813 10.1590/1414-431X20143368 PMC4075293

[454] Nusse R. Patching up hedgehog. Nature. 1996;384:119–20. 8906783 10.1038/384119a0

[455] Webster MR, Kugel CH, Weeraratna AT. The Wnts of change: how Wnts regulate phenotype switching in melanoma. Biochim Biophys Acta. 2015;1856:244–51. 26546268 10.1016/j.bbcan.2015.10.002 PMC4668201

[456] Grossmann AH, Yoo JH, Clancy J, Sorensen LK, Sedgwick A, Tong Z, et al. The small GTPase ARF6 stimulates β-catenin transcriptional activity during WNT5A-mediated melanoma invasion and metastasis*.* Sci Signal. 2013; 6:ra14. 10.1126/scisignal.2003398 PMC3961043 23462101

[457] Jobe NP, Åsberg L, Andersson T. Reduced WNT5A signaling in melanoma cells favors an amoeboid mode of invasion. Mol Oncol. 2021;15:1835–48. 33969605 10.1002/1878-0261.12974 PMC8253101

[458] Zhang J, Li Y, Wu Y, Yang T, Yang K, Wang R, et al. Wnt5a inhibits the proliferation and melanogenesis of melanocytes. Int J Med Sci. 2013;10:699–706. 23569434 10.7150/ijms.5664 PMC3619119

[459] Nomachi A, Nishita M, Inaba D, Enomoto M, Hamasaki M, Minami Y. Receptor tyrosine kinase Ror2 mediates Wnt5a-induced polarized cell migration by activating c-Jun N-terminal kinase via actin-binding protein filamin A. J Biol Chem. 2008;283:27973–81. 18667433 10.1074/jbc.M802325200

[460] Kasper M, Jaks V, Are A, Bergström Å, Schwäger A, Svärd J, et al. Wounding enhances epidermal tumorigenesis by recruiting hair follicle keratinocytes. Proc Natl Acad Sci U S A. 2011;108:4099–104. 21321199 10.1073/pnas.1014489108 PMC3054016

[461] Li X, Deng W, Lobo-Ruppert S, Ruppert J. Gli1 acts through Snail and E-cadherin to promote nuclear signaling by β-catenin. Oncogene. 2007;26:4489–98. 17297467 10.1038/sj.onc.1210241 PMC2233601

[462] Samuel MS, Lopez JI, McGhee EJ, Croft DR, Strachan D, Timpson P, et al. Actomyosin-mediated cellular tension drives increased tissue stiffness and β-catenin activation to induce epidermal hyperplasia and tumor growth. Cancer Cell. 2011;19:776–91. 21665151 10.1016/j.ccr.2011.05.008 PMC3115541

[463] Ibbetson SJ, Pyne NT, Pollard AN, Olson MF, Samuel MS. Mechanotransduction pathways promoting tumor progression are activated in invasive human squamous cell carcinoma. Am J Pathol. 2013;183:930–7. 23830873 10.1016/j.ajpath.2013.05.014

[464] Chen L, Aria AB, Silapunt S, Lee HH, Migden MR. Treatment of advanced basal cell carcinoma with sonidegib: perspective from the 30-month update of the BOLT trial. Future Oncol. 2018;14:515–25. 29119833 10.2217/fon-2017-0457

[465] Sekulic A, Migden MR, Oro AE, Dirix L, Lewis KD, Hainsworth JD, et al. Efficacy and safety of vismodegib in advanced basal-cell carcinoma. N Engl J Med. 2012;366:2171–9. 22670903 10.1056/NEJMoa1113713 PMC5278761

[466] Von Hoff DD, LoRusso PM, Rudin CM, Reddy JC, Yauch RL, Tibes R, et al. Inhibition of the hedgehog pathway in advanced basal-cell carcinoma. N Engl J Med. 2009;361:1164–72. 19726763 10.1056/NEJMoa0905360

[467] Chang ALS, Solomon JA, Hainsworth JD, Goldberg L, McKenna E, Day B-m, et al. Expanded access study of patients with advanced basal cell carcinoma treated with the Hedgehog pathway inhibitor, vismodegib*.* J Am Acad Dermatol. 2014; 70:60–69. 10.1016/j.jaad.2013.09.012 24189279

[468] Axelson M, Liu K, Jiang X, He K, Wang J, Zhao H, et al. US Food and Drug Administration approval: vismodegib for recurrent, locally advanced, or metastatic basal cell carcinoma. Clin Cancer Res. 2013;19:2289–93. 23515405 10.1158/1078-0432.CCR-12-1956

[469] Tang JY, Mackay-Wiggan JM, Aszterbaum M, Yauch RL, Lindgren J, Chang K, et al. Inhibiting the hedgehog pathway in patients with the basal-cell nevus syndrome. N Engl J Med. 2012;366:2180–8. 22670904 10.1056/NEJMoa1113538 PMC4362529

[470] Dummer R, Guminksi A, Gutzmer R, Lear J, Lewis K, Chang A, et al. Long-term efficacy and safety of sonidegib in patients with advanced basal cell carcinoma: 42-month analysis of the phase II randomized, double-blind BOLT study. Br J Dermatol. 2020;182:1369–78. 31545507 10.1111/bjd.18552 PMC7318253

[471] Papastefanou VP, René C. Secondary resistance to vismodegib after initial successful treatment of extensive recurrent periocular basal cell carcinoma with orbital invasion. Ophthalmic Plast Reconstr Surg. 2017;33:S68–70. 26398246 10.1097/IOP.0000000000000565

[472] Meani RE, Lim SW, Chang ALS, Kelly JW. Emergence of chemoresistance in a metastatic basal cell carcinoma patient after complete response to hedgehog pathway inhibitor vismodegib (GDC-0449). Australas J Dermatol. 2014;55:218–21. 25117162 10.1111/ajd.12196

[473] Chmiel P, Kłosińska M, Forma A, Pelc Z, Gęca K, Skórzewska M. Novel approaches in non-melanoma skin cancers—a focus on hedgehog pathway in basal cell carcinoma (BCC). Cells. 2022;11:3210. 36291078 10.3390/cells11203210 PMC9601130

[474] Frampton JE, Basset-Séguin N. Vismodegib: a review in advanced basal cell carcinoma. Drugs. 2018;78:1145–56. 30030732 10.1007/s40265-018-0948-9

[475] Sharpe HJ, Pau G, Dijkgraaf GJ, Basset-Seguin N, Modrusan Z, Januario T, et al. Genomic analysis of smoothened inhibitor resistance in basal cell carcinoma. Cancer Cell. 2015;27:327–41. 25759019 10.1016/j.ccell.2015.02.001 PMC5675004

[476] Atwood SX, Sarin KY, Whitson RJ, Li JR, Kim G, Rezaee M, et al. Smoothened variants explain the majority of drug resistance in basal cell carcinoma. Cancer Cell. 2015;27:342–53. 25759020 10.1016/j.ccell.2015.02.002 PMC4357167

[477] Sekulic A, Migden MR, Basset-Seguin N, Garbe C, Gesierich A, Lao CD, et al. Long-term safety and efficacy of vismodegib in patients with advanced basal cell carcinoma: final update of the pivotal ERIVANCE BCC study. BMC Cancer. 2017;17:1–10. 28511673 10.1186/s12885-017-3286-5 PMC5433030

[478] Chang AL, Oro AE. Initial assessment of tumor regrowth after vismodegib in advanced basal cell carcinoma. Arch Dermatol. 2012;148:1324–5. 22910979 10.1001/archdermatol.2012.2354 PMC3777384

[479] Lear JT, Migden MR, Lewis KD, Chang ALS, Guminski A, Gutzmer R, et al. Long-term efficacy and safety of sonidegib in patients with locally advanced and metastatic basal cell carcinoma: 30-month analysis of the randomized phase 2 BOLT study. J Eur Acad Dermatol Venereol. 2018;32:372–81. 28846163 10.1111/jdv.14542 PMC5873455

[480] Danial C, Sarin KY, Oro AE, Chang AL. An investigator-initiated open-label trial of sonidegib in advanced basal cell carcinoma patients resistant to vismodegib. Clin Cancer Res. 2016;22:1325–9. 26546616 10.1158/1078-0432.CCR-15-1588 PMC4794361

[481] Mohan SV, Chang J, Li S, Henry AS, Wood DJ, Chang AL. Increased risk of cutaneous squamous cell carcinoma after vismodegib therapy for basal cell carcinoma. JAMA Dermatol. 2016;152:527–32. 26914338 10.1001/jamadermatol.2015.4330

[482] Rübben A, Hilgers R-D, Leverkus M. Hedgehog blockade for basal cell carcinoma: Coming at a (secondary neoplastic) price. JAMA Dermatol. 2016;152:521–3. 26913776 10.1001/jamadermatol.2015.5239

[483] Kim J, Tang JY, Gong R, Kim J, Lee JJ, Clemons KV, et al. Itraconazole, a commonly used antifungal that inhibits Hedgehog pathway activity and cancer growth. Cancer Cell. 2010;17:388–99. 20385363 10.1016/j.ccr.2010.02.027 PMC4039177

[484] Kim J, Aftab B, Tang J, Kim D, Lee A, Rezaee M, et al. Itraconazole and arsenic trioxide inhibit Hedgehog pathway activation and tumor growth associated with acquired resistance to smoothened antagonists. Cancer Cell. 2013;23:23–34. 23291299 10.1016/j.ccr.2012.11.017 PMC3548977

[485] Jimeno A, Weiss GJ, Miller WH Jr, Gettinger S, Eigl BJC, Chang ALS, et al. Phase I study of the hedgehog pathway inhibitor IPI-926 in adult patients with solid tumors. Clin Cancer Res. 2013;19:2766–74. 23575478 10.1158/1078-0432.CCR-12-3654 PMC3694426

[486] Cosio T, Di Prete M, Di Raimondo C, Garofalo V, Lozzi F, Lanna C, et al. Clinical trial of patidegib gel 2%, 4%, and vehicle applied once or twice daily to decrease the GLI1 biomarker in sporadic nodular basal cell carcinomas (BCC). Available from: https://clinicaltrials.gov/study/NCT02828111. Accessed 20 Feb 2025.

[487] Kim DJ, Kim J, Spaunhurst K, Montoya J, Khodosh R, Chandra K, et al. Open-label, exploratory phase II trial of oral itraconazole for the treatment of basal cell carcinoma. J Clin Oncol. 2014;32:745–51. 24493717 10.1200/JCO.2013.49.9525

[488] Chen AC, Martin AJ, Choy B, Fernández-Peñas P, Dalziell RA, McKenzie CA, et al. A phase 3 randomized trial of nicotinamide for skin-cancer chemoprevention. N Engl J Med. 2015;373:1618–26. 26488693 10.1056/NEJMoa1506197

[489] O’Callaghan C, Vassilopoulos A. Sirtuins at the crossroads of stemness, aging, and cancer. Aging Cell. 2017;16:1208–18. 28994177 10.1111/acel.12685 PMC5676072

[490] Bubna AK. Imiquimod - Its role in the treatment of cutaneous malignancies. Indian J Pharmacol. 2015;47:354–9. 26288465 10.4103/0253-7613.161249 PMC4527053

[491] Rodon J, Argilés G, Connolly RM, Vaishampayan U, de Jonge M, Garralda E, et al. Phase 1 study of single-agent WNT974, a first-in-class Porcupine inhibitor, in patients with advanced solid tumours. Br J Cancer. 2021;125:28–37. 33941878 10.1038/s41416-021-01389-8 PMC8257624

[492] Plummer R, Dua D, Cresti N, Drew Y, Stephens P, Foegh M, et al. First-in-human study of the PARP/tankyrase inhibitor E7449 in patients with advanced solid tumours and evaluation of a novel drug-response predictor. Br J Cancer. 2020;123:525–33. 32523090 10.1038/s41416-020-0916-5 PMC7434893

[493] Dihlmann S, von Knebel DM. Wnt/β-catenin-pathway as a molecular target for future anti-cancer therapeutics. Int J Cancer. 2005;113:515–24. 15472907 10.1002/ijc.20609

[494] Gala MK, Chan AT. Molecular pathways: aspirin and Wnt signaling—a molecularly targeted approach to cancer prevention and treatment. Clin Cancer Res. 2015;21:1543–8. 25501125 10.1158/1078-0432.CCR-14-0877 PMC4383688

[495] Algazi A, Weber J, Andrews S, Urbas P, Munster P, DeConti R, et al. Phase I clinical trial of the Src inhibitor dasatinib with dacarbazine in metastatic melanoma. Br J Cancer. 2012;106:85–91. 22127285 10.1038/bjc.2011.514 PMC3251861

[496] Kalinsky K, Lee S, Rubin KM, Lawrence DP, Iafrarte AJ, Borger DR, et al. A phase 2 trial of dasatinib in patients with locally advanced or stage IV mucosal, acral, or vulvovaginal melanoma: A trial of the ECOG-ACRIN Cancer Research Group (E2607). Cancer. 2017;123:2688–97. 28334439 10.1002/cncr.30663 PMC5498223

[497] Guo J, Carvajal RD, Dummer R, Hauschild A, Daud A, Bastian BC, et al. Efficacy and safety of nilotinib in patients with KIT-mutated metastatic or inoperable melanoma: final results from the global, single-arm, phase II TEAM trial. Ann Oncol. 2017;28:1380–7. 28327988 10.1093/annonc/mdx079 PMC5452069

[498] Kluger HM, Dudek AZ, McCann C, Ritacco J, Southard N, Jilaveanu LB, et al. A phase 2 trial of dasatinib in advanced melanoma. Cancer. 2011;117:2202–8. 21523734 10.1002/cncr.25766 PMC3116034

[499] Han H, Yang B, Nakaoka HJ, Yang J, Zhao Y, Le Nguyen K, et al. Hippo signaling dysfunction induces cancer cell addiction to YAP. Oncogene. 2018;37:6414–24. 30068939 10.1038/s41388-018-0419-5 PMC6294669

[500] Munster PN, Marchion D, Thomas S, Egorin M, Minton S, Springett G, et al. Phase I trial of vorinostat and doxorubicin in solid tumours: histone deacetylase 2 expression as a predictive marker. Br J Cancer. 2009;101:1044–50. 19738609 10.1038/sj.bjc.6605293 PMC2768109

[501] Fruehauf JP, El-Masry M, Osann K, Parmakhtiar B, Yamamoto M, Jakowatz JG. Phase II study of pazopanib in combination with paclitaxel in patients with metastatic melanoma. Cancer Chemother Pharmacol. 2018;82:353–60. 29943192 10.1007/s00280-018-3624-6 PMC6060847

[502] Tolcher AW, Messersmith WA, Mikulski SM, Papadopoulos KP, Kwak EL, Gibbon DG, et al. Phase I study of RO4929097, a gamma secretase inhibitor of Notch signaling, in patients with refractory metastatic or locally advanced solid tumors. J Clin Oncol. 2012;30:2348. 22529266 10.1200/JCO.2011.36.8282 PMC5950496

[503] Schram AM, Gandhi L, Mita MM, Damstrup L, Campana F, Hidalgo M, et al. A phase Ib dose-escalation and expansion study of the oral MEK inhibitor pimasertib and PI3K/MTOR inhibitor voxtalisib in patients with advanced solid tumours. Br J Cancer. 2018;119:1471–6. 30425349 10.1038/s41416-018-0322-4 PMC6288157

[504] Dummer R, Sandhu SK, Miller WH, Butler MO, Blank CU, Muñoz-Couselo E, et al. A phase II, multicenter study of encorafenib/binimetinib followed by a rational triple-combination after progression in patients with advanced BRAF V600-mutated melanoma (LOGIC2). J Clin Oncol. 2020;38:10022–10022.

[505] Algazi AP, Rotow J, Posch C, Ortiz-Urda S, Pelayo A, Munster PN, et al. A dual pathway inhibition strategy using BKM120 combined with vemurafenib is poorly tolerated in BRAF V600(E/K) mutant advanced melanoma. Pigment Cell Melanoma Res. 2019;32:603–6. 30801911 10.1111/pcmr.12777

[506] Tolcher AW, Kurzrock R, Valero V, Gonzalez R, Heist RS, Tan AR, et al. Phase I dose-escalation trial of the oral AKT inhibitor uprosertib in combination with the oral MEK1/MEK2 inhibitor trametinib in patients with solid tumors. Cancer Chemother Pharmacol. 2020;85:673–83. 32062691 10.1007/s00280-020-04038-8

[507] Algazi AP, Esteve-Puig R, Nosrati A, Hinds B, Hobbs-Muthukumar A, Nandoskar P, et al. Dual MEK/AKT inhibition with trametinib and GSK2141795 does not yield clinical benefit in metastatic NRAS-mutant and wild-type melanoma. Pigment Cell Melanoma Res. 2018;31:110–4. 28921907 10.1111/pcmr.12644 PMC8049535

[508] Algazi AP, Moon J, Chmielowski B, Lo R, Kendra KL, Lao CD, et al. SWOG S1221: A phase 1 dose escalation study co-targeting MAPK-dependent and MAPK-independent BRAF inhibitor resistance in BRAF mutant advanced solid tumors with dabrafenib, trametinib, and GSK2141795 (ClinicalTrials. gov NCT01902173). J Clin Oncol. 2017; 35:2578.

[509] Molife L, Yan L, Vitfell-Rasmussen J, Zernhelt AM, Sullivan DM, Cassier PA, et al. Phase 1 trial of the oral AKT inhibitor MK-2206 plus carboplatin/paclitaxel, docetaxel, or erlotinib in patients with advanced solid tumors. J Hematol Oncol. 2014;7:1–12. 24387695 10.1186/1756-8722-7-1 PMC3884022

[510] Tolcher AW, Patnaik A, Papadopoulos KP, Rasco DW, Becerra CR, Allred AJ, et al. Phase I study of the MEK inhibitor trametinib in combination with the AKT inhibitor afuresertib in patients with solid tumors and multiple myeloma. Cancer Chemother Pharmacol. 2015;75:183–9. 25417902 10.1007/s00280-014-2615-5

[511] Bhutani T, Abrouk M, Sima CS, Sadetsky N, Hou J, Caro I, et al. Risk of cutaneous squamous cell carcinoma after treatment of basal cell carcinoma with vismodegib. J Am Acad Dermatol. 2017;77:713–8. 28780365 10.1016/j.jaad.2017.03.038

[512] Sekulic A, Yoo S, Kudchadkar R, Guillen J, Rogers G, Chang ALS, et al. Real-world assessment and treatment of locally advanced basal cell carcinoma: Findings from the RegiSONIC disease registry. PLoS ONE. 2022;17: e0262151. 35030185 10.1371/journal.pone.0262151 PMC8759646

[513] Ransohoff KJ, Tang JY, Sarin KY. Squamous change in basal-cell carcinoma with drug resistance. N Engl J Med. 2015;373:1079–82. 26352826 10.1056/NEJMc1504261

[514] Ingrassia J, Maher J, Cline A. A review of the risk of cutaneous squamous cell carcinoma after Vismodegib therapy. J of Skin. 2022;6:453–7.

[515] Yilmaz AS, Ozer HG, Gillespie JL, Allain DC, Bernhardt MN, Furlan KC, et al. Differential mutation frequencies in metastatic cutaneous squamous cell carcinomas versus primary tumors. Cancer. 2017;123:1184–93. 27906449 10.1002/cncr.30459 PMC5360561

[516] Bancalari B, Llombart B, Serra-Guillén C, Bernia E, Requena C, Nagore E, et al. Histologic changes during treatment with vismodegib in locally advanced basal cell carcinoma: a series of 19 cases. Am J Dermatopathol. 2019;41:711–7. 31436575 10.1097/DAD.0000000000001384

[517] Zhao X, Ponomaryov T, Ornell KJ, Zhou P, Dabral SK, Pak E, et al. RAS/MAPK activation drives resistance to SMO inhibition, metastasis, and tumor evolution in shh pathway-dependent tumors. Cancer Res. 2015;75:3623–35. 26130651 10.1158/0008-5472.CAN-14-2999-T PMC4558230

[518] Kuonen F, Huskey NE, Shankar G, Jaju P, Whitson RJ, Rieger KE, et al. Loss of primary cilia drives switching from hedgehog to Ras/MAPK pathway in resistant basal cell carcinoma. J Invest Dermatol. 2019;139:1439–48. 30707899 10.1016/j.jid.2018.11.035 PMC6591089

[519] Piteša N, Kurtović M, Bartoniček N, Gkotsi DS, Čonkaš J, Petrić T, et al. Signaling switching from hedgehog-GLI to MAPK signaling potentially serves as a compensatory mechanism in melanoma cell lines resistant to GANT-61. Biomedicines. 2023;11:1353. 37239024 10.3390/biomedicines11051353 PMC10216463

[520] Patil K, Khan AQ, Ahmad F, Kuttikrishnan S, Anver R, Mateo JM, et al. Sanguinarine triggers apoptosis in cutaneous squamous cell carcinoma cells through reactive oxygen species-dependent c-Jun N-terminal kinase signaling pathway. Front Biosci (Landmark Ed). 2024;29:40. 38287817 10.31083/j.fbl2901040

[521] Neill GW, Harrison WJ, Ikram MS, Williams TDL, Bianchi LS, Nadendla SK, et al. GLI1 repression of ERK activity correlates with colony formation and impaired migration in human epidermal keratinocytes. Carcinogenesis. 2008;29:738–46. 18281251 10.1093/carcin/bgn037

[522] Lee SM, Moon J, Redman BG, Chidiac T, Flaherty LE, Zha Y, et al. Phase 2 study of RO 4929097, a gamma-secretase inhibitor, in metastatic melanoma: SWOG 0933. Cancer. 2015;121:432–40. 25250858 10.1002/cncr.29055 PMC4304973

[523] Bonyadi Rad E, Hammerlindl H, Wels C, Popper U, Ravindran Menon D, Breiteneder H, et al. Notch4 signaling induces a mesenchymal-epithelial-like transition in melanoma cells to suppress malignant behaviors. Cancer Res. 2016;76:1690–7. 26801977 10.1158/0008-5472.CAN-15-1722 PMC5167360

[524] Vasilaki E, Kanaki Z, Stravopodis DJ, Klinakis A. Dll1 marks cells of origin of Ras-induced cancer in mouse squamous epithelia. Transl Oncol. 2018;11:1213–9. 30081298 10.1016/j.tranon.2018.07.011 PMC6083432

[525] Golan T, Messer AR, Amitai-Lange A, Melamed Z, Ohana R, Bell RE, et al. Interactions of melanoma cells with distal keratinocytes trigger metastasis via Notch signaling inhibition of MITF. Mol Cell. 2015;59:664–76. 26236014 10.1016/j.molcel.2015.06.028

[526] Del Castillo V-H, van der Weyden L, Nsengimana J, Speak AO, Sjöberg MK, Bishop DT, et al. Comparative genomics reveals that loss of lunatic fringe (LFNG) promotes melanoma metastasis. Mol Oncol. 2018;12:239–55. 29193607 10.1002/1878-0261.12161 PMC5792739

[527] Hiratochi M, Nagase H, Kuramochi Y, Koh C-S, Ohkawara T, Nakayama K. The Delta intracellular domain mediates TGF-β/Activin signaling through binding to Smads and has an important bi-directional function in the Notch-Delta signaling pathway. Nucleic Acids Res. 2007;35:912–22. 17251195 10.1093/nar/gkl1128 PMC1807952

[528] Atkinson JM, Rank KB, Zeng Y, Capen A, Yadav V, Manro JR, et al. Activating the Wnt/β-catenin pathway for the treatment of melanoma–application of LY2090314, a novel selective inhibitor of glycogen synthase kinase-3. PLoS ONE. 2015;10: e0125028. 25915038 10.1371/journal.pone.0125028 PMC4411090

[529] Zimmerman ZF, Kulikauskas RM, Bomsztyk K, Moon RT, Chien AJ. Activation of Wnt/β-catenin signaling increases apoptosis in melanoma cells treated with trail. PLoS ONE. 2013;8: e69593. 23869245 10.1371/journal.pone.0069593 PMC3711908

[530] Sato A, Yamamoto H, Sakane H, Koyama H, Kikuchi A. Wnt5a regulates distinct signalling pathways by binding to Frizzled2. EMBO J. 2010;29:41–54. 19910923 10.1038/emboj.2009.322 PMC2808370

[531] van Amerongen R, Fuerer C, Mizutani M, Nusse R. Wnt5a can both activate and repress Wnt/β-catenin signaling during mouse embryonic development. Dev Biol. 2012;369:101–14. 22771246 10.1016/j.ydbio.2012.06.020 PMC3435145

[532] Wang K, Ma F, Arai S, Wang Y, Varkaris A, Poluben L, et al. WNT5a signaling through ROR2 activates the Hippo pathway to suppress YAP1 activity and tumor growth. Cancer Res. 2023;83:1016–30. 36622276 10.1158/0008-5472.CAN-22-3003 PMC10073315

[533] Ploper D, Taelman VF, Robert L, Perez BS, Titz B, Chen HW, et al. MITF drives endolysosomal biogenesis and potentiates Wnt signaling in melanoma cells. Proc Natl Acad Sci U S A. 2015;112:E420-429. 25605940 10.1073/pnas.1424576112 PMC4321275

[534] Arozarena I, Bischof H, Gilby D, Belloni B, Dummer R, Wellbrock C. In melanoma, beta-catenin is a suppressor of invasion. Oncogene. 2011;30:4531–43. 21577209 10.1038/onc.2011.162 PMC3160497

[535] Hess AR, Seftor EA, Seftor RE, Hendrix MJ. Phosphoinositide 3-kinase regulates membrane type 1-matrix metalloproteinase (MMP) and MMP-2 activity during melanoma cell vasculogenic mimicry. Cancer Res. 2003;63:4757–62. 12941789

[536] Hess AR, Margaryan NV, Seftor EA, Hendrix MJ. Deciphering the signaling events that promote melanoma tumor cell vasculogenic mimicry and their link to embryonic vasculogenesis: role of the Eph receptors. Dev Dyn. 2007;236:3283–96. 17557303 10.1002/dvdy.21190

[537] Lacaria L, Lange JR, Goldmann WH, Rico F, Alonso JL. αvβ3 integrin expression increases elasticity in human melanoma cells. Biochem Biophys Res Commun. 2020;525:836–40. 32164941 10.1016/j.bbrc.2020.02.156

[538] Minaei E, Mueller SA, Ashford B, Thind AS, Mitchell J, Perry JR, et al. Cancer progression gene expression profiling identifies the urokinase plasminogen activator receptor as a biomarker of metastasis in cutaneous squamous cell carcinoma. Front Oncol. 2022;12: 835929. 35480116 10.3389/fonc.2022.835929 PMC9035872

[539] Vizkeleti L, Kiss T, Koroknai V, Ecsedi S, Papp O, Szasz I, et al. Altered integrin expression patterns shown by microarray in human cutaneous melanoma. Melanoma Res. 2017;27:180–8. 28234767 10.1097/CMR.0000000000000322

[540] Baart VM, van Duijn C, van Egmond SL, Dijckmeester WA, Jansen JC, Vahrmeijer AL, et al. EGFR and αvβ6 as promising targets for molecular imaging of cutaneous and mucosal squamous cell carcinoma of the head and neck region. Cancers (Basel). 2020; 12. 10.3390/cancers12061474 PMC7352159 32516897

[541] Lee J, Abdeen AA, Wycislo KL, Fan TM, Kilian KA. Interfacial geometry dictates cancer cell tumorigenicity. Nat Mater. 2016;15:856–62. 27043781 10.1038/nmat4610

[542] Sil H, Sen T, Chatterjee A. Fibronectin-integrin (alpha5beta1) modulates migration and invasion of murine melanoma cell line B16F10 by involving MMP-9. Oncol Res. 2011;19:335–48. 21936403 10.3727/096504011x13079697132925

[543] Mitra A, Chakrabarti J, Chatterjee A. Binding of alpha5 monoclonal antibody to cell surface alpha5beta1 integrin modulates MMP-2 and MMP-7 activity in B16F10 melanoma cells. J Environ Pathol Toxicol Oncol. 2003;22:167–78. 14529092 10.1615/jenvpathtoxoncol.v22.i3.20

[544] Jiao Y, Feng X, Zhan Y, Wang R, Zheng S, Liu W, et al. Matrix metalloproteinase-2 promotes αvβ3 integrin-mediated adhesion and migration of human melanoma cells by cleaving fibronectin. PLoS ONE. 2012;7: e41591. 22848537 10.1371/journal.pone.0041591 PMC3407216

[545] Mahabeleshwar GH, Byzova TV. Angiogenesis in melanoma. Semin Oncol. 2007;34:555–65. 18083379 10.1053/j.seminoncol.2007.09.009 PMC2365306

[546] Tebcherani AJ, de Andrade HF, Jr., and Sotto MN. Diagnostic utility of immunohistochemistry in distinguishing trichoepithelioma and basal cell carcinoma: evaluation using tissue microarray samples. Mod Pathol. 2012;25:1345–53. 22684216 10.1038/modpathol.2012.96

[547] Ke H, Yang Y, Lin Y, Liu L, Sun J, Massoumi R. High expression of CD34 and α6-integrin contributes to the cancer-initiating cell behaviour in ultraviolet-induced mouse skin squamous cell carcinoma. J Cancer. 2020;11:6760–7. 33123267 10.7150/jca.45819 PMC7592010

[548] Breza TS, Magro CM. CD34 expression in primary cutaneous malignant melanoma: apropos of a case and review of the aberrant melanoma phenotype. J Cutan Pathol. 2005;32:685–9. 16293181 10.1111/j.0303-6987.2005.00367.x

[549] Liu Q, Nie R, Li M, Li L, Zhou H, Lu H, et al. Identification of subtypes correlated with tumor immunity and immunotherapy in cutaneous melanoma. Comput Struct Biotechnol J. 2021;19:4472–85. 34471493 10.1016/j.csbj.2021.08.005 PMC8379294

[550] Hoek KS, Goding CR. Cancer stem cells versus phenotype-switching in melanoma. Pigment Cell Melanoma Res. 2010;23:746–59. 20726948 10.1111/j.1755-148X.2010.00757.x

[551] Ennen M, Keime C, Gambi G, Kieny A, Coassolo S, Thibault-Carpentier C, et al. MITF-high and MITF-low cells and a novel subpopulation expressing genes of both cell states contribute to intra- and intertumoral heterogeneity of primary melanoma. Clin Cancer Res. 2017;23:7097–107. 28855355 10.1158/1078-0432.CCR-17-0010

[552] Pozniak J, Pedri D, Landeloos E, Van Herck Y, Antoranz A, Vanwynsberghe L, et al. A TCF4-dependent gene regulatory network confers resistance to immunotherapy in melanoma. Cell. 2024;187:166-183.e25. 38181739 10.1016/j.cell.2023.11.037

[553] Zeng Z, Veitch M, Kelly GA, Tuong ZK, Cruz JG, Frazer IH, et al. IFN-γ critically enables the intratumoural infiltration of CXCR3(+) CD8(+) T cells to drive squamous cell carcinoma regression*.* Cancers (Basel). 2021; 13. 10.3390/cancers13092131 PMC8124943 33925140

[554] Toyoda H, Ido M, Nakanishi K, Nakano T, Kamiya H, Matsumine A, et al. Multiple cutaneous squamous cell carcinomas in a patient with interferon gamma receptor 2 (IFN gamma R2) deficiency. J Med Genet. 2010;47:631–4. 20587411 10.1136/jmg.2009.072108

[555] Herwig-Carl MC, Loeffler KU. Regression of periocular basal cell carcinoma: a report of four cases with clinicopathologic correlation. Ocul Oncol Pathol. 2020;6:107–14. 32258018 10.1159/000501370 PMC7109437

[556] Propper DJ, Chao D, Braybrooke JP, Bahl P, Thavasu P, Balkwill F, et al. Low-dose IFN-gamma induces tumor MHC expression in metastatic malignant melanoma. Clin Cancer Res. 2003;9:84–92. 12538455

[557] Davar D, Wang H, Chauvin JM, Pagliano O, Fourcade JJ, Ka M, et al. Phase Ib/II study of pembrolizumab and pegylated-interferon alfa-2b in advanced melanoma*.* J Clin Oncol. 2018; 36:Jco1800632. 10.1200/JCO.18.00632 PMC6286160 30359157

[558] Donia M, Hansen M, Sendrup SL, Iversen TZ, Ellebæk E, Andersen MH, et al. Methods to improve adoptive T-cell therapy for melanoma: IFN-γ enhances anticancer responses of cell products for infusion. J Invest Dermatol. 2013;133:545–52. 23014345 10.1038/jid.2012.336

[559] Buferne M, Chasson L, Grange M, Mas A, Arnoux F, Bertuzzi M, et al. IFNγ producing CD8(+) T cells modified to resist major immune checkpoints induce regression of MHC class I-deficient melanomas. Oncoimmunology. 2015;4: e974959. 25949872 10.4161/2162402X.2014.974959 PMC4404920

[560] Subhadarshini S, Sahoo S, Debnath S, Somarelli JA, Jolly MK. Dynamical modeling of proliferative-invasive plasticity and IFNγ signaling in melanoma reveals mechanisms of PD-L1 expression heterogeneity. J Immunother Cancer. 2023;11: e006766. 37678920 10.1136/jitc-2023-006766 PMC10496669

[561] Sævarsson T, de Lomana ALG, Sánchez Ó, van Esch V, Ragnarsson GB, Brynjólfsson SF, et al. Differentiation status determines the effects of IFNγ on the expression of PD-L1 and immunomodulatory genes in melanoma. Cell Commun Signal. 2024;22:618. 39736644 10.1186/s12964-024-01963-6 PMC11687009

[562] Otsuka A, Dreier J, Cheng PF, Nägeli M, Lehmann H, Felderer L, et al. Hedgehog pathway inhibitors promote adaptive immune responses in basal cell carcinoma. Clin Cancer Res. 2015;21:1289–97. 25593302 10.1158/1078-0432.CCR-14-2110

[563] Dessinioti C, Stratigos AJ. Immunotherapy and its timing in advanced basal cell carcinoma treatment. Dermatol Pract Concept. 2023;13: e2023252. 37992360 10.5826/dpc.1304a252 PMC10656142

[564] Chang J, Zhu GA, Cheung C, Li S, Kim J, Chang AL. Association between programmed death ligand 1 expression in patients with basal cell carcinomas and the number of treatment modalities. JAMA Dermatol. 2017;153:285–90. 28259105 10.1001/jamadermatol.2016.5062

[565] FDA approves cemiplimab-rwlc for locally advanced and metastatic basal cell carcinoma. FDA. 2022. https://www.fda.gov/drugs/resources-information-approved-drugs/fda-approves-cemiplimab-rwlc-locally-advanced-and-metastatic-basal-cell-carcinoma. 10.1080/14737140.2022.2043748 35175882

[566] Stratigos AJ, Sekulic A, Peris K, Bechter O, Prey S, Kaatz M, et al. Cemiplimab in locally advanced basal cell carcinoma after hedgehog inhibitor therapy: an open-label, multi-centre, single-arm, phase 2 trial. Lancet Oncol. 2021;22:848–57. 34000246 10.1016/S1470-2045(21)00126-1

[567] Lewis KD, Peris K, Sekulic A, Stratigos AJ, Dunn L, Eroglu Z, et al. Final analysis of phase II results with cemiplimab in metastatic basal cell carcinoma after hedgehog pathway inhibitors. Ann Oncol. 2024;35:221–8. 38072158 10.1016/j.annonc.2023.10.123

[568] Chang ALS, Tran DC, Cannon JGD, Li S, Jeng M, Patel R, et al. Pembrolizumab for advanced basal cell carcinoma: An investigator-initiated, proof-of-concept study. J Am Acad Dermatol. 2019;80:564–6. 30145186 10.1016/j.jaad.2018.08.017 PMC6839543

[569] Quandt Z, Jacob S, Fadlullah MZH, Wu C, Wu C, Huppert L, et al. Phase II trial of pembrolizumab, ipilimumab, and aspirin in melanoma: clinical outcomes and translational predictors of response. BJC Reports. 2024;2:46. 39516257 10.1038/s44276-024-00057-7 PMC11524064

[570] Spranger S, Bao R, Gajewski TF. Melanoma-intrinsic β-catenin signalling prevents anti-tumour immunity. Nature. 2015;523:231–5. 25970248 10.1038/nature14404

[571] DeVito NC, Sturdivant M, Thievanthiran B, Xiao C, Plebanek MP, Salama AKS, et al. Pharmacological Wnt ligand inhibition overcomes key tumor-mediated resistance pathways to anti-PD-1 immunotherapy. Cell Rep. 2021;35: 109071. 33951424 10.1016/j.celrep.2021.109071 PMC8148423

[572] Fukuda Y, Kim SH, Bustos MA, Cho SN, Roszik J, Burks JK, et al. Inhibition of microsomal prostaglandin E2 synthase reduces collagen deposition in melanoma tumors and may improve immunotherapy efficacy by reducing T-cell exhaustion. Cancer Res Commun. 2023;3:1397–408. 37529399 10.1158/2767-9764.CRC-23-0210 PMC10389052

[573] Wang SJ, Khullar K, Kim S, Yegya-Raman N, Malhotra J, Groisberg R, et al. Effect of cyclo-oxygenase inhibitor use during checkpoint blockade immunotherapy in patients with metastatic melanoma and non-small cell lung cancer. J Immunother Cancer. 2020;8:e000889. 10.1136/jitc-2020-000889 PMC7537331 33020239

[574] Long GV, Robert C, Butler MO, Couture F, Carlino MS, O’Day S, et al. Standard-dose pembrolizumab plus alternate-dose ipilimumab in advanced melanoma: KEYNOTE-029 cohort 1C, a phase 2 randomized study of two dosing schedules. Clin Cancer Res. 2021;27:5280–8. 34210681 10.1158/1078-0432.CCR-21-0793 PMC9401495

[575] Kakavand H, Rawson RV, Pupo GM, Yang JY, Menzies AM, Carlino MS, et al. PD-L1 expression and immune escape in melanoma resistance to MAPK inhibitors. Clin Cancer Res. 2017;23:6054–61. 28724663 10.1158/1078-0432.CCR-16-1688

[576] Kim MH, Kim CG, Kim S-K, Shin SJ, Choe EA, Park S-H, et al. YAP-Induced PD-L1 expression drives immune evasion in BRAFi-resistant melanoma YAP promotes immune evasion by PD-L1 upregulation. Cancer Immunol Res. 2018;6:255–66. 29382670 10.1158/2326-6066.CIR-17-0320

[577] Kim KB, Prieto V, Joseph RW, Diwan AH, Gallick GE, Papadopoulos NE, et al. A randomized phase II study of cilengitide (EMD 121974) in patients with metastatic melanoma. Melanoma Res. 2012;22:294–301. 22668797 10.1097/CMR.0b013e32835312e4 PMC3880198

[578] Su X, Esser AK, Amend SR, Xiang J, Xu Y, Ross MH, et al. Antagonizing integrin β3 increases immunosuppression in cancer. Cancer Res. 2016;76:3484–95. 27216180 10.1158/0008-5472.CAN-15-2663 PMC4944657

[579] Du Y, Shao H, Moller M, Prokupets R, Tse YT, Liu ZJ. Intracellular Notch1 signaling in cancer-associated fibroblasts dictates the plasticity and stemness of melanoma stem/initiating cells. Stem Cells. 2019;37:865–75. 30941836 10.1002/stem.3013 PMC6986496

